# Topological analyses of the L-lysine exporter LysO reveal a critical role for a conserved pair of intramembrane solvent-exposed acidic residues

**DOI:** 10.1016/j.jbc.2021.101168

**Published:** 2021-09-04

**Authors:** Swati Dubey, Puja Majumder, Aravind Penmatsa, Abhijit A. Sardesai

**Affiliations:** 1 Laboratory of Bacterial Genetics, Centre for DNA Fingerprinting and Diagnostics, Hyderabad, India; 2 Graduate Studies, Manipal Academy of Higher Education, Manipal, India; 3 Molecular Biophysics Unit, Indian Institute of Science, Bangalore, India

**Keywords:** membrane protein, amino acid transport, *Escherichia coli* (*E. coli*), membrane transport, bacterial metabolism, lysine export, proton-coupled antiport, *A*_*600*_, absorbance at 600 nm, ACMA, 9-amino-6-chloro-2-methoxyacridine, Ara, L-arabinose, Arg, L-arginine, Cys, L-cysteine, DSS, disuccinimidyl suberate, ECL, enhanced chemiluminescence, HA, hemagglutinin, IPTG, isopropyl β-d-1-thiogalactopyranoside, Lys, L-lysine, Lys-Ala, lysylalaninie, Mal-PEG, methoxypolyethylene glycol maleimide, MTSES, sodium (2-sulfonatoethyl) methanethiosulfonate, NEM, N-ethylmaleimide, ORF, open reading frame, P_*ara*_, promoter of the *araBAD* operon, PBS, phosphate buffered saline, P_*trc*_, *trc* promoter, SCAM, substituted cysteine accessibility method, SD, standard deviation, Thl, L-thialysine, TMS, transmembrane segment

## Abstract

LysO, a prototypical member of the LysO family, mediates export of L-lysine (Lys) and resistance to the toxic Lys antimetabolite, L-thialysine (Thl) in *Escherichia coli*. Here, we have addressed unknown aspects of LysO function pertaining to its membrane topology and the mechanism by which it mediates Lys/Thl export. Using substituted cysteine (Cys) accessibility, here we delineated the membrane topology of LysO. Our studies support a model in which both the N- and C-termini of LysO are present at the periplasmic face of the membrane with a transmembrane (TM) domain comprising eight TM segments (TMSs) between them. In addition, a feature of intramembrane solvent exposure in LysO is inferred with the identification of membrane-located solvent-exposed Cys residues. Isosteric substitutions of a pair of conserved acidic residues, one E233, located in the solvent-exposed TMS7 and the other D261, in a solvent-exposed intramembrane segment located between TMS7 and TMS8, abolished LysO function *in vivo*. Thl, but not Lys, elicited proton release in inside-out membrane vesicles, a process requiring the presence of both E233 and D261. We postulate that Thl may be exported in antiport with H^+^ and that Lys may be a low-affinity export substrate. Our findings are compatible with a physiological scenario wherein *in vivo* LysO exports the naturally occurring antimetabolite Thl with higher affinity over the essential cellular metabolite Lys, thus affording protection from Thl toxicity and limiting wasteful export of Lys.

Many microbial genomes encode integral membrane transporters, capable of mediating amino acid export (1). It is generally believed that amino acid exporters may serve to mitigate stress arising due to conditions that lead to elevation in the cellular levels of amino acids. Physiological conditions of limited catabolism of an amino acid or instances when the level of an amino acid is elevated as a by-product of overflow metabolism are thought to represent situations where the need for amino acid export is realized (2). For example, export of L-lysine (Lys) that accumulates in threonine auxotrophs of *Mycobacterium tuberculosis*, *via* the Lys exporter LysE, is thought to represent one means of adaptation to elevated cytoplasmic Lys pool (3). However, it is not clear as to what extent the aforementioned perturbations in amino acid metabolism occur in bacteria in their natural habitats. Hence the requirement for amino exporters *prima facie* appears to be enigmatic. It is easier to rationalize the existence of amino acid uptake systems as their contribution to microbial fitness is obvious. Nonetheless, it is common to find organisms encoding exporters for multiple amino acids. Both *Escherichia coli* and *Corynebacterium glutamicum* encode mono or multi-amino acid exporters for a variety of L-amino acids (reviewed in (4)). Majority of amino acid exporters are thought to perform their export function as secondary active transporters, energized by H^+^ or Na^+^ gradients across the cytoplasmic membrane (reviewed in (4)).

Two proteins LysE and LysO, in *C. glutamicum* and *E. coli*, respectively, mediate export of Lys (5, 6, 7). LysE and LysO are members of two distinct protein families bearing their mnemonics. A Lys export function has recently been ascribed to another protein MglE, expressed from a plasmid of a metagenomic library, believed to be a member of the drug/metabolite transporter superfamily (8). In *C. glutamicum* LysE mediates export of both Lys and L-arginine (Arg) (5, 9), whereas in *E. coli* Lys and Arg are exported separately by LysO and the Arg exporter ArgO, respectively (7, 10). Mutants of *C*. *glutamicum* and *E. coli* lacking LysE and LysO, respectively, display reduced fitness in media with Lys containing dipeptides that is correlated with elevated cytoplasmic Lys content following dipeptide uptake (5, 7).

A requirement for amino acid exporters in mediating resistance to toxic analogues of amino acids has been recognized. In *E. coli* absence of ArgO and LysO renders the corresponding mutants hypersensitive to L-canavanine (CAN) and L-thialysine (Thl), respectively (7, 10). CAN and Thl are naturally occurring toxic analogues (antimetabolites) of Arg and Lys, respectively (11, 12). Misincorporation of CAN and Thl in proteins during translation is believed to be causal to their toxicity *in vivo*. Besides LysO and ArgO, other examples of amino acid exporters mediating antimetabolite resistance are known (13, 14). This raises the issue that perhaps the physiological need for amino acid exporters arises for effecting export of naturally occurring antimetabolites.

Ever since the discovery of Lys export in *C. glutamicum* (15) and identification of LysE as a Lys exporter (5), numerous amino acid exporters have been characterized, primarily at a physiological level (1, 4). There, however, is a paucity of structural information on this class of membrane proteins, a requirement needed to obtain mechanistic insights into the process of amino acid export. To our knowledge, the multi-amino acid exporter YddG from *Starkeya novella* represents the only exporter for which an X-ray crystal structure is available (16). Toward obtaining some insights into the structure of LysO and the mechanism by which LysO mediates export of Lys/Thl, we have used multiple complementary approaches to determine its membrane topology *in situ*. Our results indicate that LysO adopts an N_out_-C_out_ configuration in the cytoplasmic membrane of *E. coli*. An absolute requirement for a pair of conserved acidic residues in an intramembrane solvent exposed region comprising TMS7 and an adjacent segment located between TMS7 and TMS8 for LysO function is identified. Additional observations indicate that LysO may export Thl with higher affinity than Lys, the physiological implication of which is discussed.

## Results

### Membrane topology of LysO: methodology and overview

**Figure 1:**
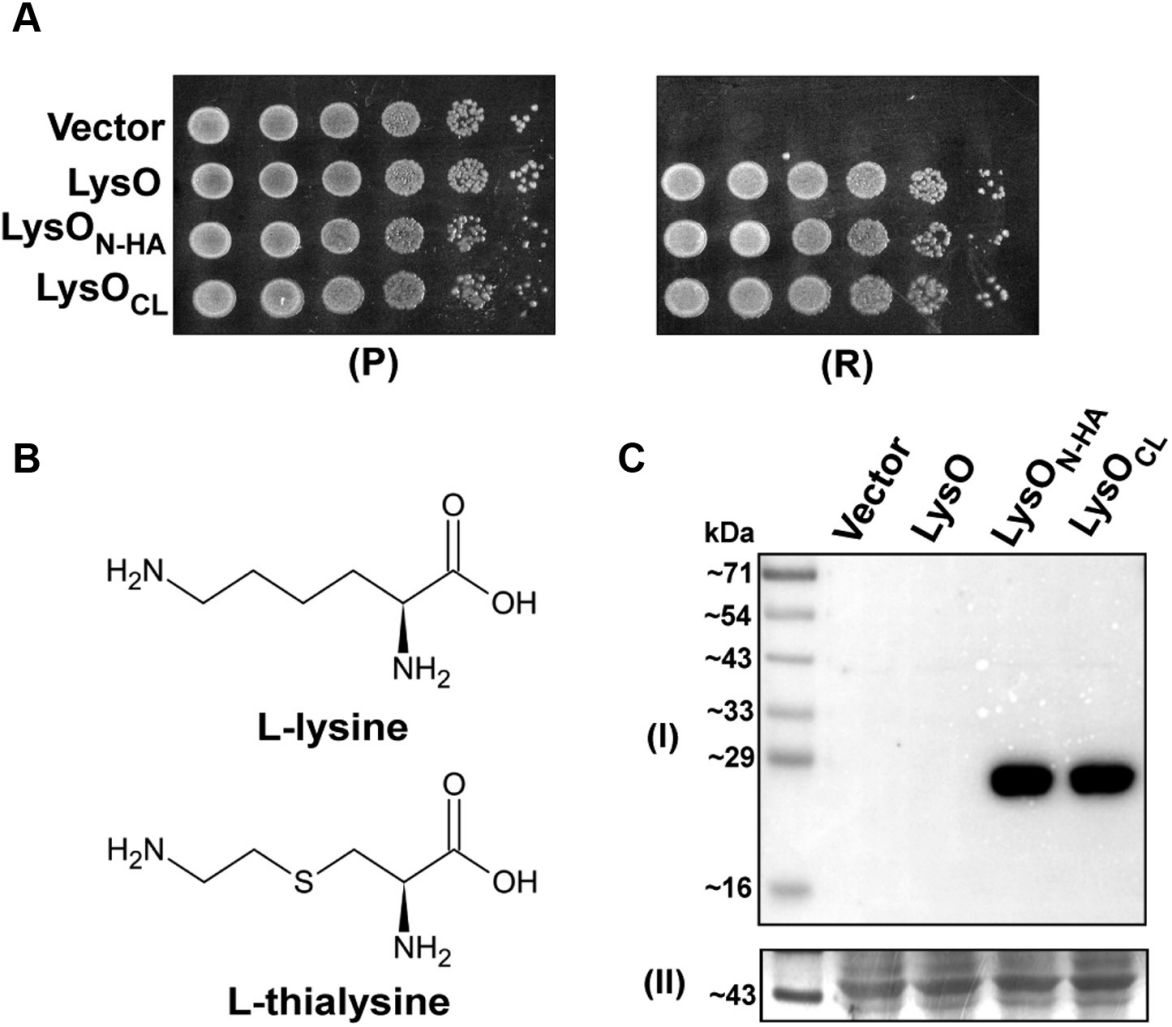
**Expression of an N-terminally HA epitope tagged LysO and its cysteine-less derivative alleviates the thialysine sensitive phenotype of the Δ*lysO* mutant.***A*, *A*_*600*_ normalized cultures of the strain GJ9026 (MC4100 Δ*lysO*::Kan) bearing the vector (pHYD5001) and its derivatives expressing LysO (LysO, plasmid pHYD2836), N-terminally HA tagged LysO (LysO_N-HA_, plasmid pHYD5579), and a cysteine-less version of LysO_N-HA_ (LysO_CL_, plasmid pHYD5580) were 10-fold serially diluted and spotted on the surface of permissive (P) and restrictive (R) growth media that are Minimal A glucose agar and Minimal A glucose agar containing thialysine at 1 μg/ml respectively. The indicated proteins are expressed from the plasmid-borne P_*trc*_ promoter. The plates also contained IPTG (at 1 mM). *B*, chemical structures of L-lysine and L-thialysine. *C*, immunodetection of LysO_N-HA_ and LysO_CL_ with anti-HA antibody. Cell extracts of GJ9026 bearing the indicated plasmids prepared from *A*_*600*_ normalized cultures were separated on SDS-PAGE, transferred to a PVDF membrane, and probed with anti-HA antibody (I). A portion of the membrane stained with Amido-Black (II) is displayed as an indicator of equal loading.

Toward determining the membrane topology of LysO, we employed two approaches, one involving determining accessibility of Cys residues introduced in LysO and the other involving use of a compartment specific reporter protein linked to N-terminal segments of LysO of varying lengths. Of the two approaches, the former is described in this section. Initially we constructed a plasmid encoding a LysO variant expressed from the P_*trc*_ promoter, bearing an appended N-terminal HA tag (LysO_N-HA_). Absence of LysO renders *E. coli* hypersensitive to the presence of the Lys antimetabolite Thl in the medium (7). As expected, expression of untagged LysO from the plasmid pHYD2836 (7) alleviated the Thl hypersensitive phenotype of the Δ*lysO* mutant, GJ9026 (Fig. 1*A*). Expression of LysO_N-HA_ and of a derivative of LysO_N-HA_ bearing the C75A, C112A and C255A substitutions also alleviated the Thl hypersensitivity of the Δ*lysO* mutant (Fig. 1*A*). The latter derivative of LysO is a cysteineless (Cysless) version of LysO_N-HA_ and is designated LysO_CL_. Immunoblotting with anti-HA antibody showed that both the aforementioned LysO variants were expressed at comparable levels (Fig. 1*C*). Although the theoretical molecular masses of LysO_N-HA_ and LysO_CL_ are 33.50 and 33.40 kDa, respectively, we noted both migrated on SDS gels as species with a molecular mass of approximately 25 kDa (Figs. 1*C*). These observations permitted the conclusion that *in vivo* LysO activity is neither perturbed by the attachment of an HA tag to its N-terminus nor is it perturbed by the replacement of its endogenous Cys residues with alanine.

**Figure 2:**
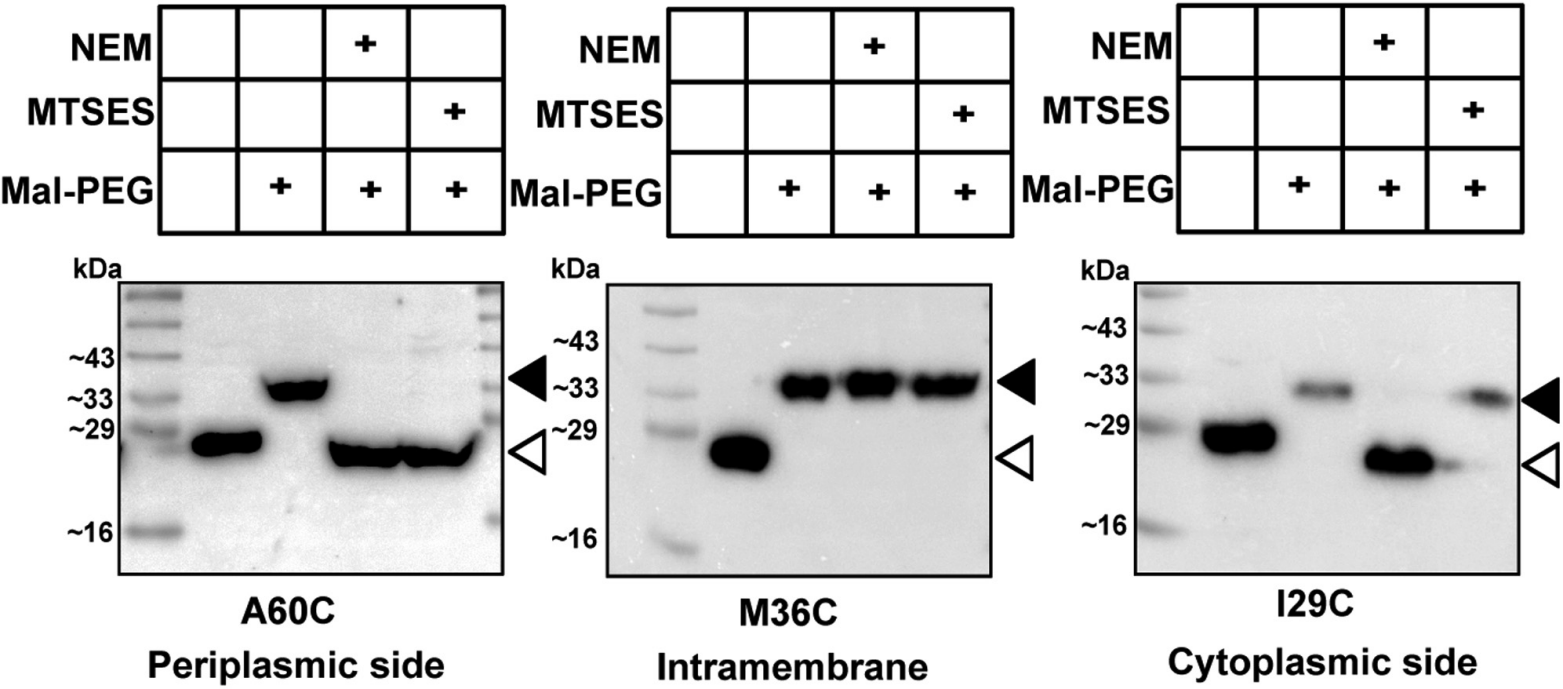
**Examples of inferred topological locations of certain substituted cysteines in LysO.** Anti-HA immunoblots depicting the patterns of modification of the indicated monocysteine substituted derivatives of the Cysless LysO_N-HA_ (LysO_CL_) following exposure to NEM and MTSES. Mid-log phase cultures of the strain GJ9026 bearing plasmids expressing the A60C, M36C, and I29C substitution derivatives (Table S2) were processed as described in the Experimental procedures. Treatments with NEM, MTSES and Mal-PEG are indicated as are the positions of free (*open triangles*) and Mal-PEG adducts of the indicated LysO derivatives (*filled triangles*). The location of the Cys residues with respect to the membrane in the A60C, I29C, and M36C Cys replacement derivatives of LysO_CL_ is indicated.

Given that LysO_CL_ was functional *in vivo*, we generated plasmids expressing its derivatives bearing Cys residues incorporated throughout the length of the protein. To large extent, amino acids in LysO displaying low conservations were replaced with Cys residues. In this way, we obtained 69 monocysteine and two dicysteine substituted derivatives of LysO_CL_. Expression of all the aforementioned derivatives alleviated the Thl hypersensitivity of the Δ*lysO* mutant (data not shown), indicating that Cys substitutions in multiple distinct positions in LysO were tolerated. Whereas all the Cys substituted derivatives retained LysO function *in vivo*, they were expressed at varying levels (data not shown). To infer the topological information for a given Cys residue, we employed the substituted cysteine accessibility method (17, 18, 19, 20). Cultures of the Δ*lysO*::Kan mutant expressing the Cys substituted LysO_CL_ variants were separately treated with NEM and MTSES. NEM being membrane permeable can modify Cys residues located in the periplasmic and the cytoplasmic sides of the membrane. MTSES being membrane impermeable can modify only the periplasmic cysteines. Both NEM and MTSES cannot react with Cys residues exposed to the hydrophobic interior of the membrane (19). Given their small sizes, both can diffuse into the periplasm from the medium presumably *via* the porin channels in the outer membrane. Mal-PEG reacts with a free Cys in a protein, which leads to increase in the molecular weight of the protein by approximately 5 kDa, that can be detected with immunoblotting (in this case) with anti-HA antibody following SDS PAGE of samples. The outcome of Cys modification with either NEM or MTSES is that the modified protein is rendered immune to labeling with Mal-PEG in the presence of SDS. Representative images of immunoblots yielding topological information on Cys residues at the 60th, 36th, and 29th positions of LysO_CL_ are depicted in Figure 2. These images permitted the inference that the 60th and the 29th amino acids of LysO are located at the periplasmic and the cytoplasmic sides of the inner membrane, respectively, whereas the 36th amino acid of LysO is located in the membrane and is lipid exposed. The reactivity to NEM and MTSES as well as the inferred locations of Cys residues incorporated in other positions in LysO is listed in Table 1. The magnitude of reactivity of a Cys substituted protein to NEM and MTSES was judged qualitatively based on the intensity of the free LysO species and the extent of formation of the LysO:Mal-PEG adducts in the immunoblots (Fig. S1 and Table 1). For example, the A60C substituted protein was considered to react strongly with NEM and MTSES, whereas the I29C substituted protein was considered to react strongly only with NEM but not with MTSES (Fig. 2). The reactivities of the Cys residues incorporated in LysO_CL_ to NEM and MTSES can be affected by the local environment of the Cys residues or by the extent of their exposure, which may lead to partial reactivity. For example, the Cys residue in the F43C substitution derivative (Cys 43) was considered to be moderately reactive to NEM, but not reactive to MTSES (Table 1). Nonetheless, Cys 43 could be placed in TMS2 between the lipid-exposed Cys 39 and the solvent-exposed Cys 49 placed near the periplasmic interface because it reacted strongly with both NEM and MTSES (Table1 and Fig. S1). Similar reasoning was employed for topological assignments of other Cys residues displaying partial reactivity.

**Table 1:** Reactivity of Cys substitutions incorporated in Cysless LysO to NEM and MTSES and their inferred locations

Substitution	Reactivity (R)/nonreactivity (NR) to NEM	Reactivity (R)/nonreactivity (NR) to MTSES	Inferred location
F2C	R^S^	R^S^	Periplasmic interface^c^^,^^d^, TMS1
I7C	R^S^	R^S^	Intramembrane, SE^c^^,^^f^, TMS1
V10C	R^W^	NR	Intramembrane^c^, TMS1
V14C	NR	NR	Intramembrane^c^, TMS1
G15C	R^S^	NR	Intramembrane, SE^c^^,^^f^, TMS1
I18C	R^S^	NR	Intramembrane, SE^c^^,^^d^, TMS1
Q22C	R^S^	NR	Cytoplasmic^c^^,^^d^, TMS1-2
I29C	R^S^	NR	Cytoplasmic interface, SE^c^^, d^, TMS2-3
L32C	R^W^	NR	Intramembrane^c^, TMS2
M36C	NR	NR	Intramembrane^c^, TMS2
L39C	NR	NR	Intramembrane^c^, TMS2
F43C	R^M^	NR	Intramembrane^c^, TMS2
A49C	R^S^	R^S^	Periplasmic interface, SE^c^, TMS2
A55C	R^S^	R^S^	Periplasmic^c^, TMS2-3
A60C	R^S^	R^S^	Periplasmic^c^^,^^d^, TMS2-3
H63C	R^S^	R^M^	Periplasmic interface, SE^c^, TMS3
V71C	NR	NR	Intramembrane^c^, TMS3
L74C	NR	NR	Intramembrane^c^, TMS3
I77C	NR	NR	Intramembrane^c^, TMS3
M81C	R^S^	NR	Cytoplasmic interface, SE^c^^,^^d^, TMS3
H92C	R^S^	NR	Cytoplasmic^c^^,^^d^, TMS3-4
A105C	R^S^	NR	Intramembrane, SE^c^^,^^d^, TMS4
K110C^a^	R^S^	NR	Intramembrane, SE^c^^,^^f^, TMS4
112C^a^	NR	NR	Intramembrane^c^^,^^f^, TMS4
V116C	NR	NR	Intramembrane^c^, TMS4
A120C	NR	NR	Intramembrane^c^, TMS4
L126C	R^S^	R^S^	Periplasmic^c^, TMS4-5
F128C	R^S^	R^S^	Periplasmic^c^, TMS4-5
A132C	R^S^	R^S^	Periplasmic interface^c^, TMS5
L142C	NR	NR	Intramembrane^c^, TMS5
V147C	NR	NR	Intramembrane^c^, TMS5
R152C	R^S^	NR	Cytoplasmic interface^c^^,^^f^, TMS5
T157C	R^S^	NR	Cytoplasmic^c^, TMS5-6
G167C	R^S^	NR	Cytoplasmic^c^, TMS5-6
V170C	R^M^	NR	Intramembrane^c^, TMS6
V172C	NR	NR	Intramembrane^c^, TMS6
V176C	NR	NR	Intramembrane^c^, TMS6
G182C	R^S^	NR	Intramembrane, SE^c^^,^^f^, TMS6
F187C	R^S^	R^S^	Intramembrane, SE^c^^,^^d^^,^^e^, TMS6
L189C	R^S^	R^W^	Intramembrane, SE^c^^,^^d^^,^^e^, TMS6
I193C	NR	NR	Intramembrane^c^^,^^e^, TMS6-7 (IR1)
A196C	NR	NR	Intramembrane^c^^,^^e^, TMS6-7 (IR1)
A198C	NR	NR	Intramembrane^c^^,^^e^, TMS6-7 (IR1)
A200C	NR	NR	Intramembrane^c^^,^^e^, TMS6-7 (IR1)
G202C	R^W^	NR	Intramembrane^c^^,^^e^, TMS6-7 (IR1)
W205C	R^S^	NR	Intramembrane, SE^c^^,^^d^^,^^e^, TMS6-7 (IR1)
S209C	R^S^	NR	Intramembrane, SE^c^^,^^d^^,^^e^, TMS6-7 (IR1)
I211C	R^S^	R^S^	Intramembrane, SE^c^^,^^d^^,^^e^, TMS6-7 (IR1)
T214C	R^S^	R^S^	Intramembrane, SE^c^^,^^d^^,^^e^, TMS6-7 (IR1)
S216C	R^S^	R^W^	Intramembrane, SE^c^^,^^d^^,^^e^, TMS6-7 (IR1)
P219C	R^S^	R^S^	Periplasmic^c^^,^^d^^,^^e^, TMS7
I221C	R^S^	R^S^	Intramembrane, SE^c^^,^^d^^,^^e^, TMS7
F226C	R^S^	NR	Intramembrane, SE^c^^,^^d^^,^^e^, TMS7
D229C	R^S^	NR	Intramembrane, SE^c^^,^^d^^,^^e^, TMS7
A231C	NR	NR	Intramembrane^c^, TMS7
I235C	NR	NR	Intramembrane^c^, TMS7
I237C	R^S^	NR	Intramembrane, SE^c^^,^^d^^,^^e^, TMS7
G242C	R^S^	NR	Intramembrane, SE^c^^,^^d^^,^^e^, TMS7
R246C	R^S^	NR	Intramembrane, SE^c^^,^^d^^,^^e^, TMS7
R248C	R^S^	NR	Intramembrane, SE^c^^,^^d^^,^^e^, TMS7-8 (IR2)
T250C	R^S^	NR	Intramembrane, SE^c^^,^^d^^,^^e^, TMS7-8 (IR2)
G253C	R^S^	NR	Intramembrane, SE^c^^,^^d^^,^^e^, TMS7-8 (IR2)
L254C^b^	R^S^	NR	Intramembrane, SE^c^^,^^e^, TMS7-8 (IR2)
255C^b^	R^S^	NR	Intramembrane, SE^c^^,^^e^, TMS7-8 (IR2)
T258C	R^S^	NR	Intramembrane, SE^c^^,^^d^^,^^e^, TMS7-8 (IR2)
V266C	R^S^	NR	Intramembrane, SE^c^^,^^d^^,^^e^, TMS7-8 (IR2)
D274C	R^S^	NR	Cytoplasmic^c^^,^^d^ TMS7-8
I285C	NR	NR	Intramembrane^c^, TMS8
L286C	NR	NR	Intramembrane^c^, TMS8
L288C	R^M^	NR	Intramembrane^c^, TMS8
V290C	NR	NR	Intramembrane^c^, TMS8
L293C	NR	NR	Intramembrane^c^, TMS8
A295C	R^S^	R^S^	Periplasmic interface^c^, TMS8

Superscript S, M, and W denote strong, moderate, and weak reactivity, respectively, to NEM and MTSES. IR1 and IR2 represent the two intramembrane regions in LysO, and SE and TMS represent solvent-exposed and transmembrane segment, respectively. For Cys residues located between two TMS, the TM numbers are separated with hyphens.

a, b Cys residues of the dicysteine substituted Cysless LysO.

c Location of Cys residue assigned using method 1.

d Location of Cys residue assigned using method 2.

e Location assigned using AlphaFold2 and RoseTTAFold predictions.

f Extrapolated location based on analyses of the locations of nearby Cys residues interrogated with methods 1 and 2.

For some Cys residues, for example, Cys 214, premodification with NEM and MTSES followed by PEGylation led to the appearance of a LysO species other than the free LysO, whose apparent molecular weight is higher than that of the LysO:Mal-PEG adduct (Fig. S1). This is seen to a lesser extent for I7C, F187C, I211C, P219C, and A295C LysO substitution derivatives (Fig. S1). This species of LysO is perhaps an aggregate, formed in some proportion following premodification. Since a LysO:Mal-PEG adduct was not detected for Cys 214 following NEM and MTSES treatment, Cys 214 was regarded as a Cys residue that reacts strongly with both the Cys modifying agents. As described later, Cys 214 was placed in an intramembrane solvent-exposed stretch of LysO located between TMS6 and TMS7, as were Cys 211 and Cys 219. For reasons outlined in the next section, we employed a variation of the Cys accessibility method to delineate the locations of some Cys residues; hence this method is referred to as method 1 in this study.

For majority of the Cys substituted variants, we noted that the free LysO species in all samples treated with Mal-PEG displayed faster mobility in comparison to the free LysO species in the sample not treated with Mal-PEG (see Fig. 2, A60C and Fig. S1, F2C, G15C, for examples). The exact reason for the altered mobility is not known. However, this does not appear to interfere in assigning the position of the LysO:Mal-PEG adduct, which in all cases examined migrated as an approximately 33.0 kDa species. In addition, we observed for some Cys substituted proteins diminished detection LysO:Mal-PEG adducts (see Fig. 2, I29C and Fig. S1, I7C, V10C, A55C, M81C, for examples), which may be attributed to inefficient transfer to the PVDF membrane of the LysO:Mal-PEG adducts. Lastly, we noted that for some Cys substituted derivatives the efficiency of formation of LysO:Mal-PEG adducts appeared to be severely diminished (see Fig. S1, Q22C and A55C). We infer that these may represent positions that are weakly modified by Mal-PEG even in the presence of SDS.

These analyses also permitted us to infer the locations of two closely spaced Cys residues in the dicysteine LysO_CL_ derivative bearing the K110C substitution along with the naturally occurring Cys residue at amino acid number 112 in LysO. While Cys at 110 is solvent-exposed (Fig. S1), subsequent analyses permitted the inference that both the Cys residues are located in TMS4 with Cys 112 being a lipid-exposed residue (Figs. S2 and S3). Similarly, we initially considered the two adjacent Cys residues of the dicysteine derivative of LysO_CL_ bearing the L254C substitution and the naturally occurring Cys 255, to be located in the cytoplasm. However, subsequent studies indicate that these represent positions in LysO that are solvent-exposed but have an intramembrane location (Figs. S1–S3). PEGylation of both Cys residues of the two dicysteine derivatives led to an increase in the molecular weights of their LysO:Mal-PEG adducts by approximately 10 kDa (Fig. S1).

**Figure 3:**
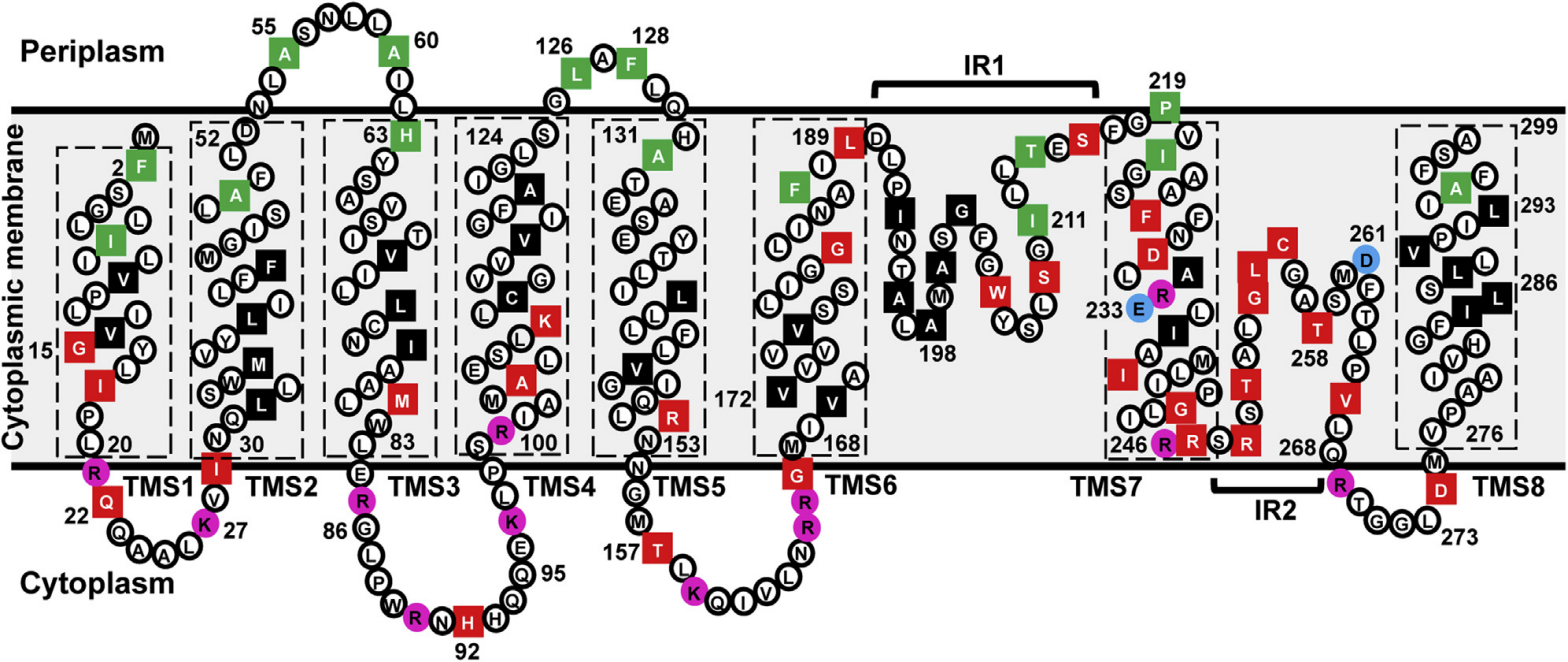
**The two-dimensional topological model of LysO.** Amino acids of LysO substituted with Cys (*filled squares*) and locations assigned to the corresponding Cys residues are indicated. *Red* and *green squares* represent Cys residues reactive to NEM and NEM and MTSES, respectively, whereas *black squares* represent Cys residues that are lipid exposed, not reacting with either NEM or MTSES. The locations of the conserved pair of acidic residues in LysO, E233, and D261 are marked with *blue circles*, and the Arg, Lys residues in LysO are indicated with *pink circles*. Arg of the R152C, R246C, R248C, and Lys of the K110C substitutions are not indicated as *pink circles*. The TMSs assigned for LysO are enclosed within *rectangles* with broken borders, and the two intramembrane regions of LysO IR1 and IR2 are marked.

With these studies and additional studies described in the next section, we were able to establish that the topology of LysO is consistent with a model wherein both its N and C-termini are present at the periplasmic face of the cytoplasmic membrane and that LysO comprises eight TM segments (TMSs, Fig. 3). TMS1, 2, 3, 4, 5, 6 are connected *via* short loops on the cytoplasmic and periplasmic sides of the membrane. Our analyses also support the existence of two intramembrane solvent-exposed regions (IRs) in LysO, one connecting TMS6 and the solvent exposed TMS7 (IR1), and the other (IR2) located between TMS7 and TMS8, at the periplasmic and cytoplasmic sides of the membrane, respectively.

Studies in support of the aforementioned feature of intramembrane solvent exposure in LysO are described in the next section. In assigning TMS lengths in the LysO model, we also took into account the previously ascribed propensities of occurrence of particular types of amino acids in the membrane interior and periphery (21, 22).

### Evidence for intramembrane solvent exposure in LysO

During the course of studies on Cys accessibility, we noted that multiple Cys residues in particular those incorporated in the region in LysO spanning the amino acids 182–266 (182–266 region) could be modified by NEM and MTSES (Fig. S1 and Table 1); however, assigning a cytoplasmic or periplasmic location to them was not straightforward. We therefore considered the possibility that these Cys residues may be located in the membrane but are solvent-exposed. To test this notion, we employed a second method (method 2) to assess the extent to which Cys residues incorporated in the aforementioned region of LysO could be directly PEGylated by Mal-PEG. In this analysis, we excluded Cys residues in the 182–266 region that were shown to be lipid-exposed by method 1 (Table 1 and Fig. S1). We used a derivative of the strain UTL2 that has a leaky outer membrane permitting the entry of Mal-PEG into the periplasm by diffusion (23, 24). Mal-PEG being membrane-impermeable reacts with a Cys residue in a periplasmic loop in intact cells, whereas it can react with one in a cytoplasmic loop only after disruption of cell membranes following sonication of the cell suspension. Cysteines buried in the membrane will not react with Mal-PEG, but will do so in the presence of SDS (Fig. S1). We expressed derivatives of LysO_CL_ bearing chosen Cys substitutions in the 187–266 region, in a derivative of UTL2, GJ16286. GJ16286 lacks endogenous LysO and expresses a cytoplasmic protein PtsN bearing a C-terminal 3× FLAG tag. We tested accessibility of the chosen substituted Cys residues to Mal-PEG (without pretreatment with NEM or MTSES), in this region both in intact and sonicated cells. LysO_CL_ derivatives bearing Cys substitutions in other positions were also included as controls. For all the chosen Cys substituted derivatives, following PEGylation, two immunoblots were generated (Figs. S2 and S3). One allowing for detection of LysO:Mal-PEG adducts and the other for the PtsN:Mal-PEG adducts. The extent of formation of the latter was taken as an indicator of cell integrity. As controls, extracts from cultures of GJ16286 expressing LysO_CL_ subjected to PEGylation in the intact and sonicated cells were also processed for immunoblotting.

Following exposure to Mal-PEG in intact cells, only Cys 60 (Cys residue at the 60th position in LysO), Cys 128, and Cys 219 could be PEGylated. This observation supports the inferred periplasmic locations of Cys 60, Cys 128 and indicates that Cys 219 is exposed to the periplasm (Figs. S1 and S2). The cell integrity under these conditions was largely maintained as judged by the absence of or very weak formation of PtsN:Mal-PEG adducts (Fig. S2).

During preparation of this manuscript, the predicted structures of LysO became available from the AlphaFold2 (AF) and the RoseTTAFold (RF) platforms ((25, 26) and Fig. S4). The AF and RF predictions show a strong overlap (Fig. S4). Multiple similarities between the two predictions and the topology of LysO inferred from this study can be discerned. The overall topology for LysO is N_out_-C_out_, with eight TMSs (Fig. 3 and Table S4). There is little variation in the assigned lengths of TMS1, 2, 3, 4, 5, and 8 (Table S4). We used the AF and RF structure predictions as templates to delineate the topology of the 182–266 region and to test the concordance of these predictions with our biochemical analyses. The AF and RF predictions allow for the delineation of the periplasmic boundary of TMS6, which is likely to be at residue number 189 (Fig. 3 and Table S4). Both Cys 187 and Cys 189 displayed strong reactivity to NEM but of the two, Cys 187 displayed strong reactivity to MTSES, whereas Cys 189 displayed weak reactivity; however, they were both not directly PEGylated with Mal-PEG (Figs. S2 and S3). These observations support the inference that these two solvent-exposed Cys residues are located near the periplasmic interface of the membrane, but they are not exposed to the periplasm. Cys 182 located in TMS6 can also be considered as solvent-exposed since it displayed strong reactivity to NEM (Fig. S1). Studies with LysO-PhoA hybrids described in the next section are supportive of the orientation of TMS6 as indicated in Figure 3.

The AF and RF models of LysO show that the 193–217 region is likely to be located in the membrane at the periplasmic side, linking TMS6 with TMS7 (Table S4 and Fig. S5). Of the tested Cys residues incorporated in this region, none was directly PEGylated in intact cells (Fig. S2), indicating that this stretch in LysO is not exposed to the periplasm. In sonicated cells, two Cys residues in the 193–217 region, Cys 211, and Cys 214 displayed moderate extent of PEGylation, whereas all others were either weakly or not PEGylated (Fig. S3). Presumably PEGylation occurs more efficiently in sonicated cells, since in intact cells its efficiency is dependent on the rate of diffusion of Mal-PEG into the periplasm. The outcome being that Cys residues with reduced exposure would be more efficiently PEGylated in sonicated cells. Moreover, apart from displaying strong reactivity to NEM, two Cys residues, Cys 211 and Cys 214, in this stretch also displayed strong reactivity to MTSES (Fig. S1). These observations indicate that the 193–217 region in LysO (IR1) is located in the membrane at the periplasmic side.

TMS7 in the AF and the RF predictions comprises a region of LysO from amino acids 219–246 (Table S4 and Fig. S4). Cys 219 was the only residue in the 182–266 region that was PEGylated in intact cells (Fig. S2); therefore its positioning as a periplasmic exposed residue at the apex of TMS7 coincides with the AF and RF predictions. Multiple Cys substitutions in TMS7 displayed reactivity to NEM. TMS7 is thus solvent-exposed (Fig. S1). Since multiple Cys residues incorporated in the region after residue number 242 displayed negligible to weak PEGylation in sonicated cells (Fig. S3), it indicates that TMS7 does not exit into the cytoplasm. We presume that Cys 246 is located at the cytoplasmic boundary of TMS7. We noted that TMS7 is capable of forming an amphipathic helix to retain regions of solvent accessibility and lipid exposure. Prediction of amphipathic helix was obtained *via* JPred ((27), data not shown) and analysis of amphipathic behavior was performed through 3D-HM ((28), data not shown). Studies with LysO-PhoA hybrids, described in the next section, are supportive of the indicated orientation of TMS7.

Existence of a second intramembrane region in LysO, between TMS7 and TMS8 at the presumed cytoplasmic side of the membrane, is predicted by AF and RF (Table S4 and Fig. S5). Our studies support the existence of this intramembrane region (IR2), its predicted location, and also show that this stretch in LysO is solvent-exposed. Of the tested Cys substitutions in the 247–268 stretch in LysO, some displayed either weak or no reactivity to Mal-PEG in sonicated cells that is indicative of their intramembrane location (Fig. S3), Moreover, all Cys substitutions in this region were reactive only to NEM but not to MTSES, indicating that the 247–268 region (IR2) lacks exposure either to the periplasm or the periplasmic interface, is located in the membrane at the cytoplasmic side and is solvent-exposed. Since glutamine is believed to have a propensity to occupy an interfacial position in TMSs (22), we presume glutamine at position 268 in IR2, to be located at the cytoplasmic interface. Given that a robust LysO:Mal-PEG adduct was detected for Cys 274, in sonicated cells (Fig. S3), this residue is certainly cytoplasmic and well exposed. Thus, a short cytoplasmic segment of LysO connects IR2 with TMS8.

Cys 22, Cys 92, and Cys 157 yielded strong LysO:Mal-PEG adducts only in sonicated cells (Fig. S3). Given that they were modified only by NEM (Fig. S1 and Table 1), their interrogation by method 2 ascertains their cytoplasmic location. Of the two Cys residues, Cys 29 and Cys 105, the former was weakly PEGylated, and the latter did not yield any PEGylated product in sonicated cells (Fig. S3), presumably because they may lie close to the interface between the cytoplasm and the membrane, leading to efficient and inefficient modification by NEM and the bulkier Mal-PEG, respectively (Figs. 3, S1 and S3). The aforementioned analyses also permitted us to infer that Cys 18 is solvent-exposed, but located in the membrane (Figs. S1 and S3) as is Cys 15, given that Cys 14 is lipid-exposed (Figs. 3 and S1). Lastly, with regard to the two closely spaced Cys residues in the LysO_CL_ derivative bearing the K110C substitution along with the naturally occurring Cys 112, both can be assigned to be located in TMS4. It is certain that of the two, one, Cys 112, is lipid-exposed and the other Cys 110 is solvent-exposed (Fig. S1).

### Correlates of LysO topology with LysO-PhoA hybrids

**Figure 4:**
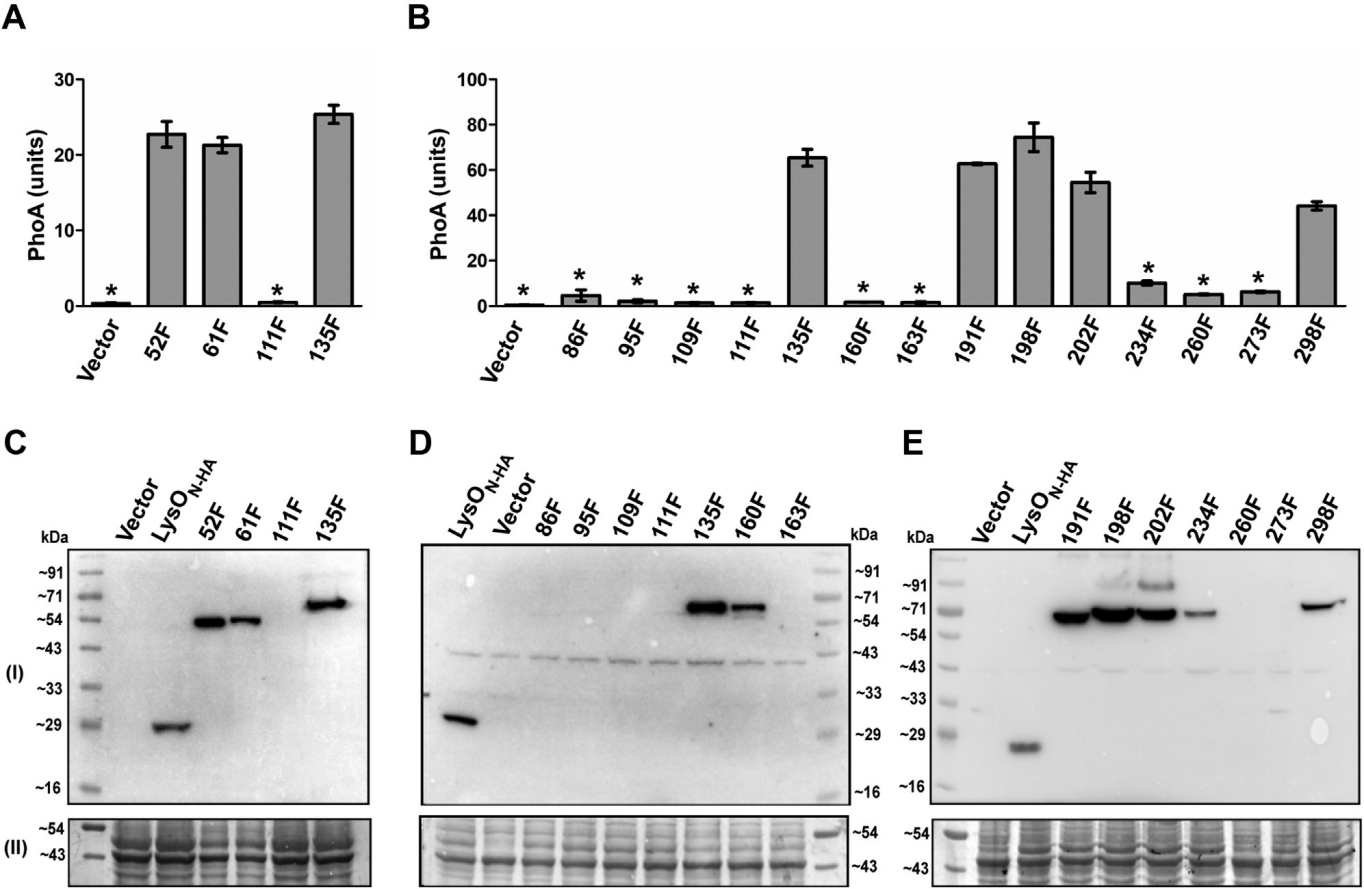
**PhoA activities of cells expressing plasmid encoded LysO**_**N-HA**_**-PhoA hybrids**. PhoA activities (units) of the indicated hybrids were assayed in a strain carrying the Δ*pcnB* mutation (GJ16373, *A*) or in its *pcnB*^+^ counterpart (GJ16281, *B*). The amino acids of LysO_N-HA_ preceding the PhoA moiety are indicated “F”. LysO_N-HA_-PhoA hybrids yielding PhoA units less than 10 in *pcnB*^+^ host and 5 in the Δ*pcnB* host are marked with *asterisks*. Exponential phase cultures of cells expressing the indicated hybrids were obtained and processed for PhoA assays. Immunodetection of LysO_N-HA_-PhoA hybrids in GJ16373 (*C*) and GJ16281 (*D* and *E*) with anti-HA antibody, panels marked (I). Indicators of equal sample loading (II) are as described for Figure 1. Plasmids expressing the LysO_N-HA_-PhoA hybrids and LysO_N-HA_ from the P_*trc*_ promoter are listed in Table S2.

We used fusions of PhoA to LysO_N-HA_, to obtain an independent assessment of topological locations of certain amino acids in LysO (29). PhoA is active in the periplasm but inactive in the cytoplasm, hence the magnitude of PhoA activity of a LysO_N-HA_-PhoA hybrid protein serves as an indicator of the location of the PhoA moiety (or of its fusion joint) of the protein. We constructed plasmids expressing LysO_N-HA_-PhoA hybrids with PhoA present after amino acids indicated in Figure 4. Since hybrids wherein PhoA was present after amino acids 52 and 61, were toxic, plasmids encoding these hybrids 52F and 61F, respectively, were recovered in a DH5α derivative lacking PcnB, wherein the copy number of ColE1 replicons is low (30, 31) and their PhoA activities were measured in a suitable strain bearing the Δ*pcnB*::Cm mutation. In the Δ*pcnB*::Cm background, PhoA activities of plasmids expressing the 111F and 135F hybrids were also determined. These served as control hybrids yielding low and high PhoA activities, respectively (Fig. 4*A*). LysO_N-HA_-PhoA hybrids 52F, 61F, 135F, 191F, 198F, 202F, and 298F yielded high PhoA activities indicating that the PhoA moiety in these hybrids is located in the periplasm with the fusion joints located either in the periplasm or near the periplasmic interface (Fig. 4, *A* and *B*). This assignment correlates with the periplasmic locations attributed to the Cys 55, Cys 63, Cys 132, and Cys 295 substitutions in LysO (Fig. S1). On the other hand, the fusion joints in the 191F, 198F, and 202F hybrids serve as markers for the indicated orientation of TMS6 and IR1 (Fig. 3). PhoA fusions to amino acids 86, 95, 109, 111, 160, 163, 234, 260, and 273 yielded very low to negligible PhoA activities. (Fig. 4*B*). Earlier studies with LacY-PhoA and TetA-PhoA hybrids have noted that multiple hybrids with fusion joints in the cytoplasm tended to be unstable (32, 33). Barring two exceptions of the 160F and the 234F hybrids, all low PhoA activity yielding hybrids did not give rise to detectable expression of the hybrid proteins, accounting for their negligible PhoA activities (Fig. 4, *C–E*). Assigning a cytoplasmic location to the PhoA moiety in these hybrids is therefore tentative, supported only by observations drawn from studies on the LacY-PhoA and TetA-PhoA hybrids described above. On the other hand, hybrids with assigned periplasmic location of the PhoA moiety were expressed at much higher levels, higher in many instances than LysO_N-HA_ (Fig. 4, *C–E*). The 160F and the 234F hybrids were expressed at lower level in comparison to other hybrids and yielded very low PhoA activities. It is likely that the PhoA moiety in these hybrids is cytoplasmic. Of all the LysO_N-HA_-PhoA hybrids, expression of only one hybrid the 298F hybrid complemented the Thl hypersensitivity of the Δ*lysO* mutant (data not shown). Overall, the limited analyses of LysO topology using the PhoA reporter technique correlated well with the Cys accessibility data, for fusions with an inferred periplasmic location, of the PhoA moiety.

### A conserved pair of negatively charged residues is required for LysO function

**Figure 5:**
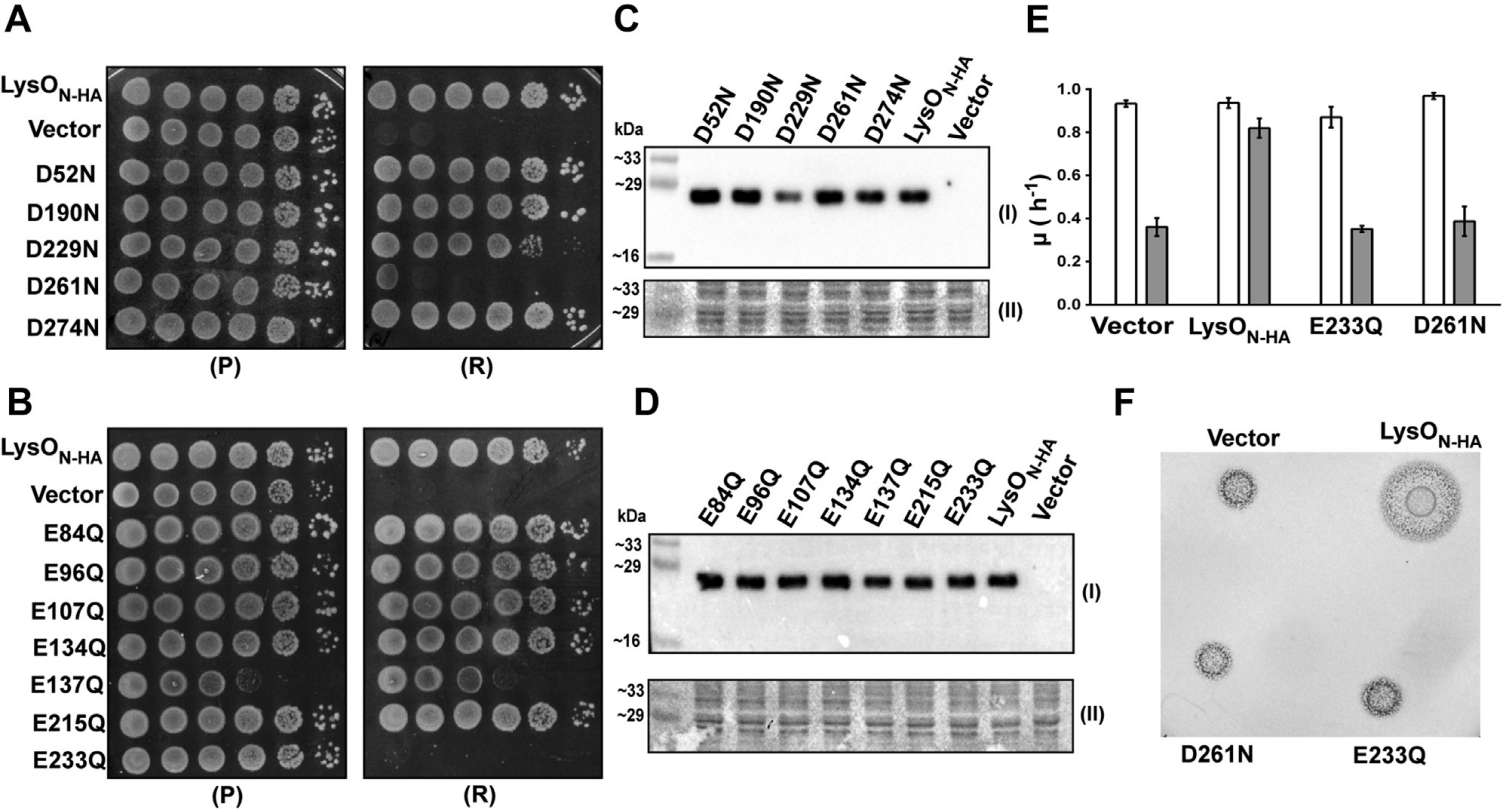
**Requirement of E233 and D261 for LysO function.** Tenfold serial dilutions of cultures of GJ9026 bearing the vector (pHYD5001) and plasmids expressing LysO_N-HA_ and its derivatives with the indicated aspartate to asparagine (*A*) and glutamate to glutamine (*B*) substitutions were spotted on the surface of IPTG (1 mM) supplemented permissive (P) and restrictive (R) growth media, namely Minimal A glucose agar and Minimal A glucose agar containing Thl at 1 μg/ml respectively. *C* and *D*, immunodetection of the expression of LysO_N-HA_ and its indicated substitution bearing derivatives with anti-HA antibody (I) and indication of equal sample loading (II) performed as described in Figure 1. *E*, effect of the Lys-Ala dipeptide on the growth of the Δ*lysO*::Kan mutant GJ9026 expressing LysO_N-HA_ and its E233Q and D261N substitution derivatives. Growth rates of GJ9026 bearing the vector and plasmids expressing LysO_N-HA_ and its E233Q and D261N substitution derivatives, following growth in glucose Minimal A medium with (*gray bars*) and without (*white bars*) the Lys-Ala dipeptide (1 mM). The growth medium also contained IPTG (1 mM). *F*, impairment of Lys export caused by the E233Q and D261N substitutions in LysO. Five microliter of *A*_*600*_ normalized cultures of GJ9026 bearing the vector and plasmids expressing LysO_N-HA_ and its E233Q and D261N substitution derivatives were spotted on the surface of a Minimal A glucose agar plate containing IPTG (1 mM) and Lys-Ala (1 mM) and tetrazolium chloride (2 μg/ml) that was seeded with 10^5^ cells of the strain GJ9060 bearing the vector pHYD5001. The plate was photographed after 30 h of growth. A grayscale version of the photograph is shown. All LysO proteins are expressed from the plasmid-borne P_*trc*_ promoter and the corresponding plasmids are listed in Table S2. Cultures bearing these plasmids were washed thrice with glucose Minimal A medium prior to *A*_*600*_ normalization and spotting.

Since both Lys and Thl are cationic in nature and negatively charged amino acids in exporters have been shown to play important roles in mediating translocation of cationic substrate and proton coupling (24, 34, 35, 36), we tested the requirement of these amino acids for LysO function. Perusal of the alignment of the amino acid sequences of LysO orthologues revealed an absolute conservation of two negatively charged amino acids of LysO, namely E233 and D261 (Fig. S6). To test the importance of these residues in LysO function, we constructed LysO_N-HA_ derivatives bearing isosteric substitutions of glutamate to glutamine (E233Q) and aspartate to asparagine (D261N) and tested the ability of the substituted LysO mutants to complement the Thl hypersensitive phenotype of the Δ*lysO* mutant. In addition, LysO_N-HA_ derivatives bearing E to Q and D to N substitutions of all other glutamates and aspartates numbering 6 and 4, respectively, were constructed and their ability to complement the Thl hypersensitivity of the Δ*lysO* mutant was also evaluated. Barring the E233Q and D261N substitution derivatives, expression of all other E to Q and D to N substitution derivatives complemented the Thl hypersensitive of the Δ*lysO* mutant (Fig. 5, *A* and *B*). Expression of all LysO derivatives employed in this study was comparable with the exception of the D229N derivative that was expressed at a low level. (Fig. 5, *C* and *D*). Previously we have shown that absence of LysO compromises the fitness of the corresponding strain when challenged with the Lys-Ala dipeptide in the medium, a phenotype that is associated with elevated intracellular Lys level (7). Lys-Ala in the medium caused a modest reduction in the growth rate of the Δ*lysO* mutant bearing the plasmid expressing LysO_N-HA_. However, it caused a marked reduction in the growth rates of the Δ*lysO* mutant containing the vector and plasmids expressing the E233Q and D261N substitution derivatives of LysO_N-HA_ (Fig. 5*E*). Growth rates of the Δ*lysO* mutant expressing the aforementioned proteins were comparable in a medium lacking Lys-Ala (Fig. 5*E*). To test whether the ability to export Lys was also compromised in the aforementioned substitution bearing derivatives, we employed a cross-feeding assay that has been described earlier (7). In this assay, the donor strain in this case the Δ*lysO* mutant bearing a given LysO expressing plasmid (or the vector) is capable of Lys-Ala uptake, whereas the recipient strain, GJ9060, lacks all peptide uptake systems and also lacks LysA. GJ9060 is therefore incapable of Lys-Ala uptake and is rendered auxotrophic for Lys. The recipient cannot utilize Lys-Ala to fulfill its Lys auxotrophy but can grow if Lys is provided (7). Cultures of the donor strain are spotted on a plate supplemented with Lys-Ala that has been seeded with the recipient, bearing the vector. The Lys exported by the donor following catabolism of Lys-Ala within the donor cross-feeds the recipient cells in its vicinity leading to zone of growth of the recipient. Because the recipient lacks LysA, the enzyme performing the last step of Lys biosynthesis (37), it can grow in minimal medium only if Lys is available. Expression of LysO_N-HA_ but not its E233Q or D261N substitution bearing derivatives in the donor GJ9026 led to cross-feeding of the recipient GJ9060, (Fig. 5*F*). Taken together the aforementioned observations show that the E233 and the D261 residues in LysO are required both for mediating resistance to Thl and for Lys export. In the topology model depicted in Figure 3, E233 and D261 respectively are located in an intramembrane solvent-exposed region in LysO, comprising TMS7 and an adjacent segment IR2 between TMS7 and 8.

### Thialysine elicits LysO-dependent proton release in inside-out membrane vesicles

**Figure 6:**
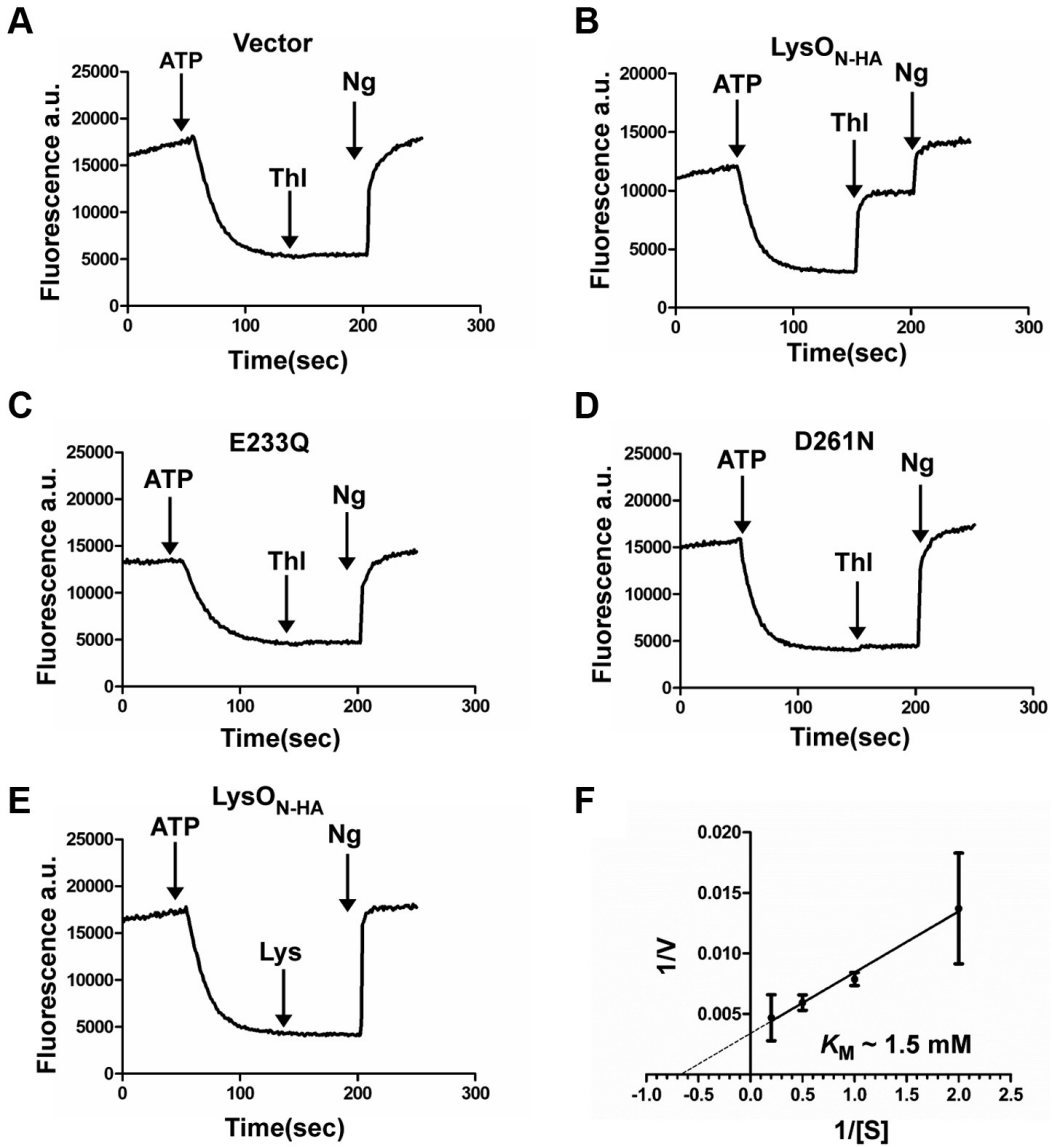
**Thialysine induced LysO mediated proton flux in inside-out vesicles and its dependence on E233 and D261 of LysO.** Inside-out vesicles were prepared from the strain GJ16375 bearing the plasmid pBAD18 (vector) and GJ16375 bearing plasmid overexpressed LysO_N-HA_ and its E233Q and D261N derivatives. Plasmids used for overexpression of LysO_N-HA_ and its E233Q and D261N substituted derivatives are pHYD6240, pHYD6402, and pHYD6401, respectively. Vesicle suspensions were incubated with the pH sensitive fluorescent dye ACMA. Quenching of ACMA fluorescence following addition of ATP, its recovery following Thl addition (*A–D*, 5 mM), and its eventual recovery after addition of the uncoupler nigericin (Ng) were recorded. ATP, Thl, and Ng were added to vesicle preparations at the 50th, 150th, and 200th second. Lys (5 mM) was added as a substrate to the vesicle preparation in (*E*), bearing overexpressed LysO_N-HA_. *F*, Lineweaver–Burk plot indicating the estimated *K*_*M*_ for Thl export. Experiments in panels (*A–E*) were performed twice with single batches of inside-out vesicle preparations of GJ16375 bearing the indicated plasmids and traces from one trial are shown.

To obtain insights into the mechanism of Thl/Lys export, mediated by LysO, we employed previously described assays for detecting substrate-induced proton release in inside-out vesicles (34, 38). Inside-out vesicles were prepared from the strain GJ16375 that lacks both LysO and ArgO, bearing overexpressed LysO_N-HA_ and its E233Q and D261N substitution derivatives (Fig. S7). Aliquots of vesicles were treated with the pH gradient-sensitive dye ACMA and loaded with protons *via* the activity of the F_0_F_1_ ATPase, by the addition of ATP. This led to quenching of ACMA fluorescence as the pH gradient was established. Following this, either Thl or Lys (each at 5 mM) was added and dequenching of ACMA fluorescence as a consequence of proton release was monitored. Thl addition to the external solution elicited proton release from the vesicles bearing overexpressed LysO_N-HA_ but not in those bearing the vector (Fig. 6, *A* and *B*). Furthermore, this proton release required the presence of the conserved E233 and D261 acidic pair (Fig. 6, *C* and *D*). Increasing concentrations of Thl led to increased magnitudes of proton release in LysO_N-HA_ bearing vesicles (Fig. S8), and from this we were able to arrive at an estimate of the apparent *K_M_* for Thl export by LysO_N-HA_, which was approximately 1.5 mM (Fig. 6*F* and see Experimental procedures) using the Lineweaver–Burk plot. Thl is a derivative of Cys; however, Cys (at 5 mM) addition did not elicit any detectable proton release in inside-out vesicles containing overexpressed LysO_N-HA_ (Fig. S9). Surprisingly, addition of Lys did not provoke detectable proton release (Fig. 6*E*), in LysO_N-HA_ bearing vesicles, the same was the case when Arg (at 5 mM) was added to the aforementioned vesicle preparation (Fig. S9). Altogether, observations in this section indicate that LysO functions as a secondary active transporter, mediating export of Thl *via* proton-coupled antiport. However, the inability of Lys to elicit proton release appears enigmatic and is discussed later.

### The *in vivo* functional state of LysO is likely to be monomeric

**Figure 7:**
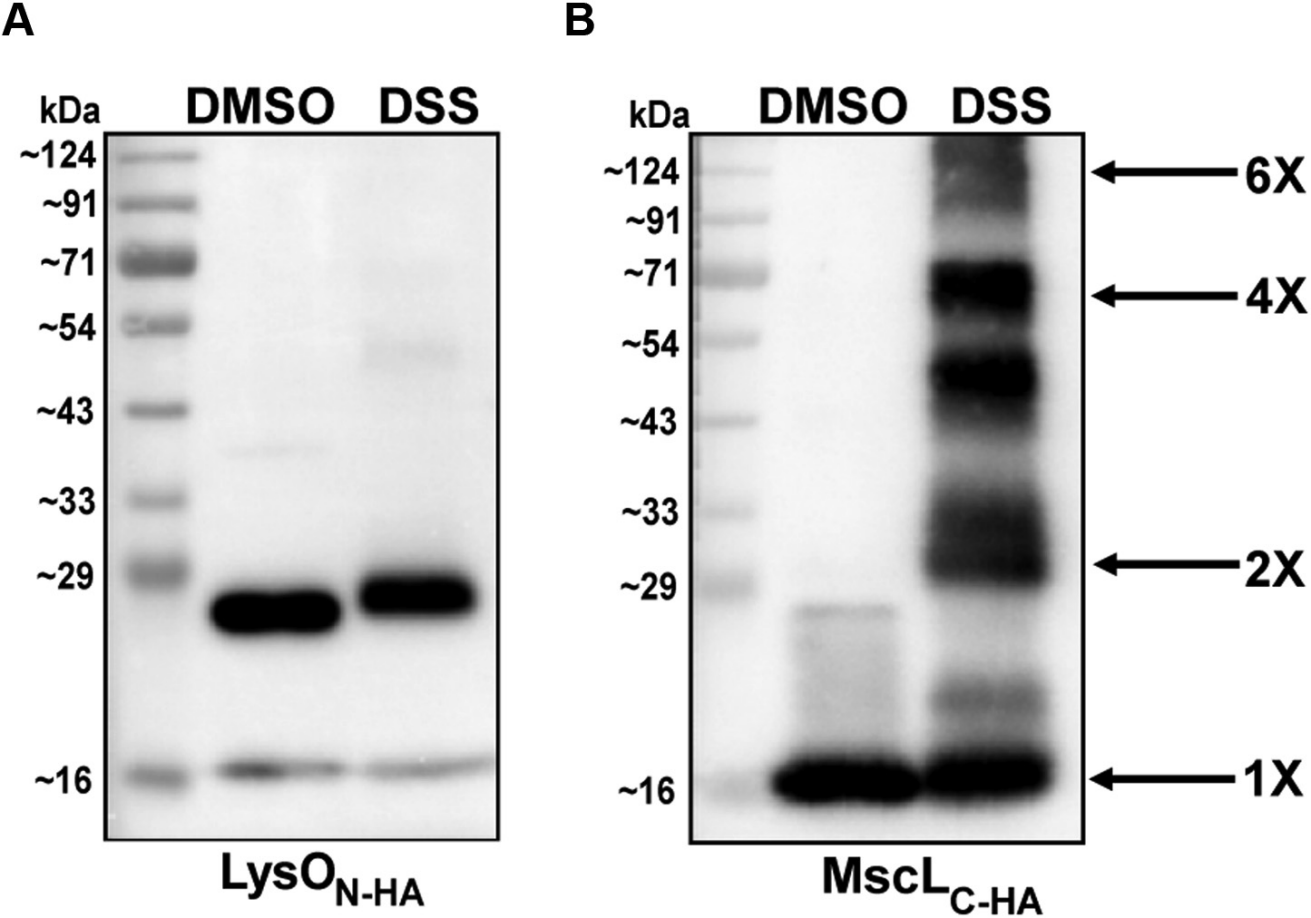
**A monomeric state of LysO *in vivo*.** Anti-HA immunoblots depicting the electrophoretic mobility of (*A*) LysO_N-HA_ and (*B*) MscL_C-HA_ following exposure of crude membranes preparations of strains overexpressing the two proteins to the primary amine crosslinker DSS or DMSO. Cultures of the strains GJ9026 and GJ16372 bearing plasmids expressing LysO_N-HA_ and MscL_C-HA_ respectively (Tables S1 and S2) were processed as described in Experimental procedures. Crude membrane preparations were treated with DSS (1 mM) or with its solvent DMSO. Following quenching of the cross-linking reaction aliquots of solubilized membrane preparations were loaded on SDS-PAGE gels and subjected to Western blotting with anti-HA antibody. The monomeric and oligomeric species of MscL are indicated.

Toward determining the oligomeric state of LysO *in vivo*, we treated crude membrane preparations from the Δ*lysO* mutant, GJ9026, overexpressing LysO_N-HA_ with the primary amine cross-linker DSS and with the DSS solvent DMSO. Crude membrane preparations from the Δ*mscL*::Kan mutant, GJ16372, overexpressing a C-terminally HA epitope tagged version of the mechanosensitive channel MscL (39) were also treated as above. Following cross-linking and immunoblotting, distinct oligomeric species of MscL_C-HA_ were detected, as reported earlier ((40) and Fig. 7*B*). However, for LysO_N-HA_, cross-linking with DSS yielded a single species corresponding to a monomer (Fig. 7*A*). The slightly retarded mobility of LysO_N-HA_ following DSS treatment may be due to formation of an intramolecular crosslink(s). The HA moiety in the aforementioned two proteins lacks primary amines. To further substantiate the notion that LysO exists as a monomer *in vivo*, the effects of overexpression of LysO_N-HA_ bearing the E233Q and D261N disabling substitutions on chromosomally encoded LysO function were tested. It was reasoned that if the *in vivo* functional state of LysO was an oligomer, at the least a dimer, then overexpression of a defective derivative of LysO would perturb LysO function and lead to a Thl sensitive phenotype. This rationale has earlier been employed to infer that the *in vivo* functional state of the multidrug exporter EmrE is an oligomer (41). Overexpression of the E233Q and D261N substitution derivatives of LysO_N-HA_ in the wild-type strain MC4100 did not lead to any discernible sensitivity to Thl, whereas MC4100 and its Δ*lysO*::Kan derivative, GJ9026, bearing the vector displayed resistance and hypersensitivity respectively to Thl (Fig. S10). These observations are additionally supportive of the notion that the *in vivo* functional state of LysO is a monomer.

## Discussion

Many tools for prediction of topology of ɑ-helical membrane proteins are available, and multiple approaches to improve their accuracy and reliability have been described (42, 43, 44, 45). Nonetheless, it is believed that the upper limit for accuracy of prediction is 70–80% (46, 47). Certain types of structural elements in membrane proteins such as reentrant loops, long tilted or interfacial helices are believed to be barriers to prediction accuracy (47). Despite these issues, prediction tools still serve as primary guides for detailed topology mapping. For the sake of this discussion, these prediction tools are referred to as traditional tools, which are the first eight tools listed in Table S4. Recently, artificial-intelligence-based machine learning methods are believed to lead to significantly enhanced improvements in predicting protein structures including those of membrane proteins. These are typified by the AlphaFold2 (AF) and the RoseTTAFold (RF) platforms (25, 26, 48). In this study, we have employed complementary approaches to arrive at a topological model for the Lys exporter LysO. Of the eight traditional tools listed in Table S4, three, namely TMHMM, SCAMPI, and MEMSAT-SVM, predict an overall topology of LysO that is in agreement with our study, which is N_out_-C_out_. Variation in the LysO topology prediction occurs after TMS6, which tentatively indicated that one is encountering a region of unusual topology. Our *in situ* biochemical analyses of LysO topology clearly indicate that region of LysO from amino acids 182–266 is intramembrane and likely to have an unusual topology. Our data best fit the 2D topology predictions derived from AF and RF, the concordance being very high (Table S4). This work validates the AF and RF predictions for a newly characterized integral membrane transporter with a heretofore unknown topology. The topology of LysO conforms to the positive-inside rule (49), and the biased distribution of Arg and Lys residues in LysO is apparent (Fig. 3).

The two acidic residues E233 and D261 located in the solvent-exposed TMS7-IR2 region (Figs. 3 and S5) were found to be required for all aspects of LysO function tested in this study (Figs. 5 and 6). However, the mechanism by which these residues coordinate Thl/Lys export remains to be investigated. It is possible that they may play a mutually interdependent role in substrate export mediated by LysO. Acidic residues in the transport vestibule are capable of undergoing protonation and deprotonation events to mediate transport as observed in numerous proton-coupled efflux transporters (50). Substrates can competitively interact with protonation sites to mediate transport and can be assisted by the presence of additional protonation sites that can facilitate electrogenic transport. A well-studied example of such behavior is MdfA that has two acidic residues E26 and D34 that mediate antibacterial efflux in this proton-coupled antiporter. While D34 is responsible for competitive proton–substrate interactions, E26 located toward the cytosolic half of the vestibule can facilitate proton transfer *via* sequential binding of substrate and protons at D34 (51). The topology of LysO is distinct from major facilitator superfamily antiporters; nonetheless, the proton–substrate coupling mechanisms could still retain similarities to MdfA within the transport vestibule of LysO.

Despite no sequence or functional similarities, the topology of LysO resembles an inversion of the topology of a monomer of the glutamate/aspartate:Na^+^ symporter Glt_Ph_, a member of the solute carrier 1 family (52, 53). GltPh is an obligate trimer with each monomer containing eight TMSs organized in an N_in_-C_in_ configuration (52, 53). The intramembrane hairpin loops (HP), HP1 and HP2 in the transport domain of a monomer, are located between TMS6-TMS7 and TMS7-TMS8, respectively. HP loops in Glt_Ph_ harbor the substrate and sodium-binding sites. The alternating access observed in Glt_Ph_ and related proteins has been proposed to occur through an “elevator-mechanism” wherein the hairpin loops move vertical to the plane of the membrane to mediate substrate transport (53, 54). The TMS6-HP1-TMS7-HP2-TMS8 arrangement of GltPh is analogous to the TMS6-IR1-TMS7-IR2-TMS8 arrangement in LysO, albeit inverted (Table S4 and Fig. S5). Owing to the similarities in topological organization of LysO with Glt_Ph_, we presume that proton-coupled antiport in LysO is likely to follow an elevator mechanism, occurring within a monomer of LysO. Based on our topology and functional studies, the transport domain of LysO appears to be the TMS6-IR1-TMS7-IR2-TMS8 segment with the remaining TMSs likely playing a scaffolding role in the transport process.

Thl but not Lys elicited proton release in inside-out vesicles (Fig. 6). While the Thl effect supports the notion that LysO functions as a secondary active transporter presumably mediating Thl export in antiport with protons, the absence of a similar effect by Lys is enigmatic. The notion that LysO is a Lys exporter is not anecdotal, the two acidic residues required for Thl resistance and Thl mediated proton release are also required for Lys export (Fig. 5, *E* and *F*). Moreover, LysO-mediated Lys excretion into the medium has been demonstrated (6, 7). This discrepancy can be rationalized if one postulates that in comparison to Thl, Lys may be a very low-affinity export substrate. Indeed, amino acid export in general is believed to be a low-affinity export process (55). If the aforementioned postulate is valid, then one outcome of the differing affinities of Lys and Thl could be that under steady-state conditions of microbial growth, wasteful export of Lys, an essential amino acid, would be minimal, whereas high-affinity export of its toxic analogue Thl would prove advantageous in an environment where Thl is present with LysO activity offering rapid protection from Thl toxicity. Export of an amino acid in principle can lead to its uptake by amino acid uptake systems, setting up an energy-consuming futile cycle of export and uptake (2), which can be attenuated considerably if export is rendered low affinity. Futile cycling of Thl is also expected when it is present in the environment, its uptake occurring *via* transporters that mediate Lys uptake (56, 57) and export *via* LysO. However, Thl futile cycling would be expected to be short term, lasting till the environment changes.

In summary, in this report we have delineated the topology of LysO, obtained insights into its export mechanism and have identified a pair of acidic residues critical for LysO function. Future studies in this regard will involve testing the AF and RF predictions on helix coalescence, export vestibule formation in LysO, and identification of determinants in LysO that govern Lys/Thl affinity.

## Experimental procedures

### Bacterial strains and growth conditions

Bacterial strains used in this study are derivatives of *E. coli* K-12 and their genotypes are described in Table S1. LB and glucose Minimal A media were used as rich and synthetic media respectively (58), and the temperature for bacterial cultivation was 37 °C. Whenever required the two media were supplemented with Isopropyl β-d-1-thiogalactopyranoside (IPTG), L-arabinose (Ara), and antibiotics at concentrations as appropriate. The antibiotics ampicillin and kanamycin were routinely used at appropriate concentrations in growth media, and ampicillin was included in all media for plasmid selection. Strain construction was performed using phage P1 transductions (58). In this study, deletions insertion mutations in *lysO* (Δ*lysO*::Kan), *argO* (Δ*argO*::Kan), *phoA* (Δ*phoA*::Kan), and *mscL* (Δ*mscL*::Kan) were sourced from appropriate strains of the Keio collection (59) and were introduced into other strains using P1 transduction. Whenever required the antibiotic markers in the *lysO*, *argO*, *phoA* lesions were excised following treatment with the plasmid pCP20 (60).

### Plasmid construction

Plasmids used in this study are derivatives of the plasmid pTrc99A and pBAD18. Plasmids and oligonucleotide primers used for their construction are described in Tables S2 and S3 respectively. Standard molecular biology procedures for cloning, PCR, and site-directed mutagenesis were employed for plasmid construction (61), and the veracity of cloned inserts was ascertained with DNA sequencing. An open reading frame (ORF) encoding LysO bearing an N-terminally abutted hemagglutinin (HA) epitope tag, *lysO*_N-HA_, was constructed by PCR and placed under the expression control of the P_*trc*_ promoter of a derivative of the plasmid pTrc99A (Table S2). *lysO*_N-HA_, is designed, such that the initiation codon of *lysO* is linked to 12 codons encoding the HA epitope and the Gly Gly Pro linker followed by rest of the codons of *lysO*, and the encoded protein is designated LysO_N-HA_. The amino acid numbering for LysO (or LysO_N-HA_) indicated throughout this study is the natural numbering and contribution from the amino acids of HA and the linker is ignored. Amino acid substitutions were introduced in *lysO*_N-HA_ *via* overlap extension PCR, leading to the generation of several of its derivatives (Table S2).

Plasmids expressing LysO_N-HA_-PhoA hybrids were constructed in a two-step procedure. In the first step, the *phoA* gene lacking its first 26 codons (encoding its signal sequence) was placed between the SalI and HindIII sites of the plasmid pHYD5537 generating the plasmid pHYD5517 (Table S2). Segments of varying lengths of *lysO*_N-HA_ DNA, all bearing its initiation codon, were placed between the NdeI SalI sites of pHYD5517. Thus, plasmids encoding LysO_N-HA_-PhoA hybrids proteins, each with a fixed N-terminus and C-terminus of varying lengths, linked to signal sequenceless PhoA were generated. In all the encoded hybrid proteins, valine and aspartic acid residues, encoded from codons in the SalI restriction site, are sandwiched between the LysO_N-HA_ and the PhoA moieties. All the aforementioned hybrid proteins are under the expression control of the plasmid borne P_*trc*_ promoter.

### Growth test for LysO function and detection of Lys cross-feeding

The functionality of plasmid encoded LysO and its derivatives was assessed by their ability (or inability) to confer upon a Δ*lysO*::Kan mutant GJ9026 (7), resistance to L-thialysine (*S*-(2-aminoethyl)- L-cysteine, Thl), a toxic analogue of Lys. Overnight cultures of GJ9026, bearing appropriate plasmids and corresponding vector were normalized to an *A*_*600*_ of 2, 10-fold serially diluted, with dilutions ranging from 10^−1^ to 10^−6^, in minimal medium and 5 μl of various dilutions were spotted on glucose Minimal A agar plate with and without Thl (1 μg/ml). The plates were also supplemented with IPTG (at 1 mM). In the instance of assessing the effect of the dipeptide lysylalaninie (Lys-Ala) on bacterial growth rate (for Fig. 5*E*), Lys-Ala was added to glucose Minimal A medium (broth) at 1 mM and IPTG was also included at 1 mM. Growth rates were determined following growth of the indicated strains in a Varioskan Flash microplate reader at 37 °C, and results are presented as mean ± SD of two independent measurements. Lys export capacity of plasmid expressed LysO_N-HA_ and its derivatives was assessed by a Lys cross-feeding assay as described earlier (7).

### Assay of alkaline phosphatase (PhoA)

Logarithmically growing cultures of the strains bearing plasmids expressing LysO_N-HA_-PhoA hybrids under the expression control of the P_*trc*_ promoter were obtained after cultivation in LB broth supplemented IPTG. The cultures were processed for PhoA assay as described in Pathania *et al.* (39). Values of PhoA units represent mean ± SD of two independent measurements performed in duplicate. GJ16281 was used as the host strain for expression of hybrids in which the PhoA moiety was located after amino acid numbers 86, 95, 109, 111, 135, 160, 163, 191, 198, 202, 234, 260, 273, and 298 of LysO_N-HA_ and IPTG (at 10 μM) was present during culture growth. A Δ*pcnB*::Cm derivative of GJ16281, GJ16373, was the host for expressing hybrids bearing fusions of PhoA to amino acids 52 and 61 of LysO_N-HA_. GJ16373 bearing plasmids encoding the aforementioned two hybrid proteins was cultured in LB broth containing 1 mM IPTG.

### Detection of LysO_N-HA_ by immunoblotting

Expression of LysO_N-HA_ and its derivatives was detected by immunoblotting with anti-HA antibody. Mid-exponential phase cultures of strains bearing the appropriate plasmids, cultivated in LB broth with IPTG added as required, were normalized to an *A*_*600*_ of 1.0 (for Figs. 1*C* and 5, *C* and *D*) and centrifuged at 10,000 rpm at room temperature. Bacterial pellets were washed with phosphate buffered saline (PBS, pH-7.4) solubilized in 1× SDS loading buffer, sonicated, and loaded onto 12% SDS-PAGE gels. After electrophoresis, proteins were transferred to PVDF membranes by semidry transfer. Transferred proteins were blocked in buffer C (50 mM Tris-HCl pH 7.5, 150 mM NaCl, 0.01% Tween 20, and 5% fat-free milk) and probed with anti-HA antibody (1:10,000) overnight. Following washes with buffer C lacking fat-free milk, the blots were probed with the appropriate horseradish-peroxidase-conjugated secondary antibody (1:10,000) for 1 h. Immunoblots were developed using the ECL kit (GE Healthcare) and visualized on a UVITEC Cambridge imaging system.

For detection of LysO_N-HA_-PhoA hybrids, cultures obtained from *pcnB*^+^ and Δ*pcnB* host strains were *A*_*600*_ normalized to 1 and 2, respectively. Culture pellets were washed with PBS. Pellets were solubilized in solution A (5% SDS, 100 mM DTT), briefly sonicated, and the solubilized protein was precipitation by the methanol/chloroform extraction method (62). Protein precipitates were solubilized in solution A. Following addition of appropriate volumes of 2× SDS loading buffer, samples were loaded onto 12% SDS-PAGE gels. LysO_N-HA_-PhoA hybrids were detected as described above. Representative images of immunoblots obtained following two independent procedures are displayed throughout this study.

### Substituted cysteine (Cys) accessibility

Two methods were employed to gauge the accessibility of Cys residues introduced into a cysteine-less (Cysless) version of LysO_N-HA_ (LysO_CL_). The first method (method 1) is the classical substituted cysteine accessibility method (SCAM) (17, 18, 19, 20). The procedure for SCAM was adopted from Butler *et al.* (19), with minor modifications. Mid-exponential phase cultures of the strain GJ9026, bearing plasmids encoding Cys substituted derivatives of LysO_CL_ cultivated in LB broth with the appropriate IPTG (but varying) concentrations (Fig. S1), were harvested and washed with PBS. In most cases cell harvests were suspended in 200 μl of PBS, at an *A*_*600*_ of 2 per 200 μl. However, some Cys substituted derivatives of LysO_CL_ were expressed at low levels and in these cases, cells were resuspended at an *A*_*600*_ of 3 per 200 μl. Resuspended cells were distributed in four 50 μl aliquots in four microfuge tubes. Two aliquots were treated separately with the cysteine sulfhydryl blockers *N*-ethylmaleimide (NEM) and sodium (2-sulfonatoethyl) methanethiosulfonate (MTSES), each at a final concentration of 5 mM. The four tubes were placed at room temperature for 60 min in dark and subjected to intermittent mild agitation. Cells were washed twice with PBS and resuspended in 50 μl of lysis buffer (15 mM Tris-HCl, pH 7.4, 1% SDS, 6 M urea). Of the four aliquots, the two blocked samples and one sample not exposed to either NEM or MTSES were labeled with Mal-PEG (methoxypolyethylene glycol maleimide, molecular weight 5000) present at 5 mM, and the last sample was treated with DMSO (solvent control). Samples were kept in dark at room temperature for 60 min and intermittently agitated mildly. Further sample processing was as per Butler *et al.* (19) except that the four samples were sonicated for 5 min, prior to SDS PAGE loading. Following SDS PAGE, the gels were processed for immunoblotting with anti-HA antibody.

SCAM was also performed for some single Cys substituted variants of LysO_CL_ using a second method (method 2) that involves use of the strain UTL2 that has a leaky outer membrane (23, 24). For these studies, a derivative of UTL2, GJ16286 was employed. Method 2 was performed as described earlier (39), with minor modifications that are described in the legend for Figure S2.

### Preparation of inside-out vesicles

Inside-out vesicles were prepared from the strain GJ16375 that lacks the Lys and Arg exporters LysO and ArgO, respectively (Table S1) as described earlier (34) with minor modifications. GJ16375 harboring plasmids that express LysO_N-HA_ and its derivatives bearing the E233Q and D261N substitutions (Table S2) from the Ara inducible P_*ara*_ promoter were cultured in 800 ml of LB broth at 37 °C till an *A*_*600*_ of 0.6. Expression of LysO_N-HA_ and its derivatives was induced by the addition of 0.1% Ara to the cultures, which were incubated further for 8 h at 20 °C. All other procedures for preparation and harvesting inside-out vesicles were identical to those described in reference 34. Aliquots of the inside-out vesicle preparations were frozen in liquid nitrogen and stored at –80 °C for further use. Expression levels of LysO proteins in inside-out vesicles were detected by immunoblotting with anti-HA antibody (Fig. S7).

### Detection of substrate-induced proton release in inside-out membrane vesicles

Kinetic measurements of proton fluxes induced by Lys, Thl, and other amino acids were performed essentially as described earlier with minor modifications (34). Frozen vesicles were thawed at room temperature, and 20 μl of vesicles from vector sample and 40 μl of vesicles from the LysO expressing samples were utilized for assays of proton fluxes. This volume adjustment corresponded to nearly equal amounts of total protein in the various samples as judged by Amido Black staining of the PVDF membrane following detection of overexpressed proteins by anti-HA antibody (Fig. S7). Moreover, the fluorescence counts of ACMA (9-amino-6-chloro-2-methoxyacridine) at time zero in each kinetic measurement ranged from 12,000 to 17,000 in all measurements, indicating that variations in vesicle content from sample to sample were minimal. Appropriate volumes of membrane vesicles were diluted in a 2 ml solution of 50 mM KCl and 10 mM MgSO_4_. ACMA and valinomycin at 10 μM and 0.25 μM respectively were added at the initiation of the kinetic measurement. Fluorescence of ACMA (λ_Ex_ 409 nm, λ_Em_ 474 nm) was recorded over a period of 250 s with samples subjected to continuous stirring. The pH gradient across membrane was generated by the addition of ATP (0.25 mM) after 50 s and measured by quenching of ACMA's fluorescence. The export substrates Lys, Thl, and other L-amino acids were added after 150 s. The change in pH due to substrate-induced proton release reflected in dequenching of ACMA fluorescence till it reached a new steady state was recorded. Following addition of nigericin (4 μM) after 200 s, the measurements were terminated after 250 s.

### Estimate of apparent *K*_M_ for thialysine export

Kinetic measurements for Thl-induced proton release were performed with inside-out vesicles overexpressing LysO_N-HA_ with thialysine present at 0.5, 1, 2, and 5 mM (Fig. S8). Following thialysine addition to ATP energized vesicles at the 150th second of the measurements, seven time points in the interval between the 156th and 162nd second were taken and their corresponding arbitrary units (a.u) of ACMA fluorescence were sourced. Plots of a.u *versus* time were generated, and slopes obtained after linear regression yielded the rates of proton release for the indicated concentration of thialysine. The Lineweaver–Burk plot of the reciprocal of rate (1/V) *versus* reciprocal of substrate concentrations (1/S) yielded an estimate of *K*_M_.

### Cross-linking *in vivo* with disuccinimidyl suberate (DSS)

A modified version of the procedure described in reference 40 was employed to perform *in vivo* cross-linking with DSS. The strains GJ9026 (MC4100 Δ*lysO*::Kan) and GJ16372 (MC4100 Δ*mscL*::Kan) bearing the plasmid pHYD5579, encoding LysO_N-HA_ and the plasmid pHYD2868 (39) encoding MscL_C-HA_ (MscL bearing a C-terminal HA tag) respectively, were cultured in 50 ml of LB with ampicillin and 1 mM IPTG. LysO_N-HA_ and MscL_C-HA_ are expressed from the P_*trc*_ promoter in the aforementioned plasmids. Cells were cultured till mid-log phase, normalized for *A*_*600*_ of 25, pelleted, washed in reaction buffer (30 mM sodium phosphate, pH 7.5 and 100 mM NaCl), and resuspended in 5 ml of the same buffer. Cells were broken using a Constant Systems cell disruptor, at 30 kpsi, and the suspension was centrifuged at 10,000 rpm for 10 min to remove cell debris and unbroken cells. The supernatant was subjected to an ultracentrifugation step, at 48,000*g* for 90 min. The recovered crude membrane pellet was resuspended in 4 ml of reaction buffer. Two milliliter of the membrane suspension was transferred to two tubes each containing 1 ml of the suspension. DSS (at 1 mM) was added into one tube and the other received an equal volume of DMSO. The tubes were rotated for 30 min at room temperature, the reaction was quenched with the addition Tris-HCl pH 8 (100 mM). The membrane suspension was pelleted by ultracentrifugation at 48,000*g* for 30 min and solubilized in 100 μl of SDS loading buffer. Samples were loaded and separated in 15% SDS-PAGE, and protein detection was performed by immunoblotting with anti-HA antibody.

### Sourcing the AlphaFold2 and RoseTTAFold predictions of LysO

Predicted models of LysO were obtained through AlphaFold2 (25) and RoseTTAFold (26) using online resources. For the AlphaFold2 prediction the LysO protein sequence was submitted as the query sequence *via* google colaboratory open access resource, and the first of multiple solutions was used as the template for the structure of LysO. The AlphaFold2 prediction contained side chain information. In order to generate the RoseTTAFold model, the package containing deep learning models and related scripts were downloaded from GitHub repository to run RoseTTAFold. The LysO model was generated using the LysO protein sequence utilizing a three-track neural network. The RoseTTAFold prediction for LysO was a poly-alanine model, to minimize the computational load. Both the predicted solutions had no stereochemical outliers and overlay with each other with an r.m.s.d of 4.3 for all the Cα atoms. 2D topologies for both predictions were inferred by using endpoints of helices to define TMS boundaries of the predicted AF and RF models (Table S4).

## Data availability

All data pertaining to this manuscript are available in the main text and supporting information, and the corresponding author Dr Abhijit A. Sardesai, CDFD, Hyderabad, can be contacted for any queries (email: abhijit@cdfd.org.in)

## Supporting information

This article contains supporting information (7, 23, 25, 26, 39, 44, 59, 62, 63, 64, 65, 66, 67, 68, 69, 70, 71, 72, 73, 74, 75).

## Conflict of interest

The authors declare no conflicts of interest with the contents of this manuscript.
