# Supplementary material for: Topological analyses of the L-lysine exporter LysO reveal a critical role for a conserved pair of intramembrane solvent-exposed acidic residues
Source: J Biol Chem. 2021 Sep 4;297(4):101168. doi: 10.1016/j.jbc.2021.101168 (PMC8498466; doi:10.1016/j.jbc.2021.101168)
Supplement: Supplementary file 1 — Figures S1–S10 and Tables S1–S4 [file mmc1.pdf]

Supporting information for

Topological analyses of the L-lysine exporter LysO reveal a critical role for a conserved pair of intramembrane solvent-exposed acidic residues

Swati Dubey[1, 2], Puja Majumder[3], Aravind Penmatsa[3] and Abhijit A. Sardesai[1, *]

[1]Laboratory of Bacterial Genetics, Centre for DNA Fingerprinting and Diagnostics, Hyderabad, India.

[2]Graduate Studies, Manipal Academy of Higher Education, Manipal, India.

[3]Molecular Biophysics Unit, Indian Institute of Science, Bangalore, India.

Author for correspondence abhijit@cdfd.org.in.

Supporting information includes:

Supplementary Table S1
Supplementary Table S2
Supplementary Table S3
Supplementary Table S4

Supplementary Figure S1
Supplementary Figure S2
Supplementary Figure S3
Supplementary Figure S4
Supplementary Figure S5
Supplementary Figure S6
Supplementary Figure S7
Supplementary Figure S8
Supplementary Figure S9
Supplementary Figure S10
References

Table S1 *E. coli* strains used in this study[a]

Strain	Genotype	Source
MC4100	Δ(*argF-lac*) *U169 rpsL150 relA1 spoT1 araD139 flbB5301 deoC1 ptsF25*	Laboratory stock
LMG194	F^- Δ*lacX74 galE thi rpsL* Δ*phoA* Δ*ara714 leu*::Tn*10*	Laboratory stock
UTL2	F^- *araC14 leuB6 secA206 lacY1 proC14 tsx-67* Δ*(ompT-fepC)266 entA403 glnX44 trpE38 rfbC1 rpsL109 xylA5 mtl-1 thiE1*	Reference 23
GJ9026	MC4100 Δ*lysO*::Kan	Reference 7
GJ9060	MC4100 Δ*lysA* Δ*dppB* Δ*oppB* Δ*tppB*::Kan	Reference 7
GJ16281	MC4100 Δ*lysO* Δ*phoA*::Kan	This study
GJ16286	UTL2 $ptsN^F$::FRT Δ*lysO*::Kan	This study
GJ16372	MC4100 Δ*mscL*::Kan	This study
GJ16373	MC4100 Δ*lysO* Δ*phoA* Δ*pcnB*::Cm	This study
GJ16375	LMG194 Δ*argO* Δ*lysO*::Kan	This study

[a]All strains are derivatives of *E. coli* K-12. The Δ*lysO*::Kan, Δ*argO*::Kan, Δ*phoA*::Kan and the Δ*mscL*::Kan mutations were sourced from strains from the Keio collection (59). The $ptsN^F$ allele encodes PtsN abutted with a C-terminal 3X FLAG tag and its construction is described in reference 63. The Δ*pcnB*::Cm mutation was sourced from the strain GJ18884 (MG1655 Δ*pcnB*::Cm) of the laboratory collection. These mutations were introduced into other strains by P1 transduction for strain construction purposes.

Table S2 Plasmids used in this study

Plasmid	Description
pHYD5001	Derivative of the plasmid pTrc99A, in which an NdeI site in the vector backbone was destroyed by end filing and a new NdeI site was introduced in the MCS (multiple cloning site), in place of an NcoI site.
pHYD5537	Derivative of the plasmid pHYD5001, bearing a 1.4kb segment of DNA, encoding the Tn*10*dCm element, from the lambda phage λNK1324 and is present in the BamH1 site of pHYD5001.
pHYD5579	Derivative of the plasmid pHYD5537, encoding $LysO_{N-HA}$ with the corresponding gene, present between NdeI and HindIII sites.
pHYD5580	Derivative of pHYD5579, encoding a cysteine-less derivative of $LysO_{N-HA}$ ($LysO_{CL}$) bearing the C75A, C112A and C255A substitutions.
pHYD6229	A pHYD5580 derivative encoding $LysO_{CL}$ with the F2C substitution.
pHYD6266	A pHYD5580 derivative encoding $LysO_{CL}$ with the I7C substitution.
pHYD6267	A pHYD5580 derivative encoding $LysO_{CL}$ with the V10C substitution.
pHYD6268	A pHYD5580 derivative encoding $LysO_{CL}$ with the V14C substitution.

pHYD6434	A pHYD5580 derivative encoding LysO$_{CL}$ with the G15C substitution.
pHYD6210	A pHYD5580 derivative encoding LysO$_{CL}$ with the I18C substitution.
pHYD6259	A pHYD5580 derivative encoding LysO$_{CL}$ with the Q22C substitution.
pHYD6422	A pHYD5580 derivative encoding LysO$_{CL}$ with the I29C substitution.
pHYD6211	A pHYD5580 derivative encoding LysO$_{CL}$ with the L32C substitution.
pHYD6457	A pHYD5580 derivative encoding LysO$_{CL}$ with the M36C substitution.
pHYD6212	A pHYD5580 derivative encoding LysO$_{CL}$ with the L39C substitution.
pHYD6213	A pHYD5580 derivative encoding LysO$_{CL}$ with the F43C substitution.
pHYD6435	A pHYD5580 derivative encoding LysO$_{CL}$ with the A49C substitution.
pHYD6230	A pHYD5580 derivative encoding LysO$_{CL}$ with the A55C substitution.
pHYD6214	A pHYD5580 derivative encoding LysOCL with the A60C substitution.
pHYD6215	A pHYD5580 derivative encoding LysO$_{CL}$ with the H63C substitution.

pHYD6260	A pHYD5580 derivative encoding LysO$_{CL}$ with the V71C substitution.
pHYD6458	A pHYD5580 derivative encoding LysO$_{CL}$ with the L74C substitution.
pHYD6405	A pHYD5580 derivative encoding LysO$_{CL}$ with the I77C substitution.
pHYD6261	A pHYD5580 derivative encoding LysO$_{CL}$ with the M81C substitution.
pHYD6423	A pHYD5580 derivative encoding LysO$_{CL}$ with the H92C substitution.
pHYD6424	A pHYD5580 derivative encoding LysO$_{CL}$ with the A105C substitution.
pHYD6425	A pHYD5580 derivative encoding LysO$_{CL}$ with the K110C and 112C substitutions.
pHYD6262	A pHYD5580 derivative encoding LysO$_{CL}$ with the V116C substitution.
pHYD6216	A pHYD5580 derivative encoding LysO$_{CL}$ with the A120C substitution.
pHYD6436	A pHYD5580 derivative encoding LysO$_{CL}$ with the L126C substitution.
pHYD6217	A pHYD5580 derivative encoding LysO$_{CL}$ with the F128C substitution.
pHYD6218	A pHYD5580 derivative encoding LysO$_{CL}$ with the A132C substitution.

pHYD6219	A pHYD5580 derivative encoding $LysO_{CL}$ with the L142C substitution.
pHYD6407	A pHYD5580 derivative encoding $LysO_{CL}$ with the V147C substitution.
pHYD6426	A pHYD5580 derivative encoding $LysO_{CL}$ with the R152C substitution.
pHYD6220	A pHYD5580 derivative encoding $LysO_{CL}$ with the T157C substitution.
pHYD6427	A pHYD5580 derivative encoding $LysO_{CL}$ with the G167C substitution.
pHYD6428	A pHYD5580 derivative encoding $LysO_{CL}$ with the V170C substitution.
pHYD6263	A pHYD5580 derivative encoding $LysO_{CL}$ with the V172C substitution.
pHYD6444	A pHYD5580 derivative encoding $LysO_{CL}$ with the V176C substitution.
pHYD6445	A pHYD5580 derivative encoding $LysO_{CL}$ with the G182C substitution.
pHYD6221	A pHYD5580 derivative encoding $LysO_{CL}$ with the F187C substitution.
pHYD6406	A pHYD5580 derivative encoding $LysO_{CL}$ with the L189C substitution.
pHYD6269	A pHYD5580 derivative encoding $LysO_{CL}$ with the I193C substitution.

pHYD6222	A pHYD5580 derivative encoding $LysO_{CL}$ with the A196C substitution
pHYD6459	A pHYD5580 derivative encoding $LysO_{CL}$ with the A198C substitution.
pHYD6264	A pHYD5580 derivative encoding $LysO_{CL}$ with the A200C substitution.
pHYD6409	A pHYD5580 derivative encoding $LysO_{CL}$ with the G202C substitution.
pHYD6410	A pHYD5580 derivative encoding $LysO_{CL}$ with the W205C substitution.
pHYD6460	A pHYD5580 derivative encoding $LysO_{CL}$ with the S209C substitution.
pHYD6411	A pHYD5580 derivative encoding $LysO_{CL}$ with the I211C substitution.
pHYD6223	A pHYD5580 derivative encoding $LysO_{CL}$ with the T214C substitution.
pHYD6446	A pHYD5580 derivative encoding $LysO_{CL}$ with the S216C substitution.
pHYD6447	A pHYD5580 derivative encoding $LysO_{CL}$ with the P219C substitution.
pHYD6265	A pHYD5580 derivative encoding $LysO_{CL}$ with the I221C substitution.
pHYD6437	A pHYD5580 derivative encoding $LysO_{CL}$ with the F226C substitution.

pHYD6412	A pHYD5580 derivative encoding $LysO_{CL}$ with the D229C substitution.
pHYD6224	A pHYD5580 derivative encoding $LysO_{CL}$ with the A231C substitution.
pHYD6461	A pHYD5580 derivative encoding $LysO_{CL}$ with the I235C substitution.
pHYD6448	A pHYD5580 derivative encoding $LysO_{CL}$ with the I237C substitution.
pHYD6430	A pHYD5580 derivative encoding $LysO_{CL}$ with the G242C substitution.
pHYD6408	A pHYD5580 derivative encoding $LysO_{CL}$ with the R246C substitution.
pHYD6438	A pHYD5580 derivative encoding $LysO_{CL}$ with the R248C substitution.
pHYD6449	A pHYD5580 derivative encoding $LysO_{CL}$ with the T250C substitution.
pHYD6431	A pHYD5580 derivative encoding $LysO_{CL}$ with the G253C substitution.
pHYD6432	A pHYD5580 derivative encoding $LysO_{CL}$ with the L254C and 255C substitutions.
pHYD6415	A pHYD5580 derivative encoding $LysO_{CL}$ with the T258C substitution.
pHYD6416	A pHYD5580 derivative encoding $LysO_{CL}$ with the V266C substitution.

pHYD6270	A pHYD5580 derivative encoding $LysO_{CL}$ with the D274C substitution.
pHYD6417	A pHYD5580 derivative encoding $LysO_{CL}$ with the I285C substitution.
pHYD6433	A pHYD5580 derivative encoding $LysO_{CL}$ with the L286C substitution.
pHYD6413	A pHYD5580 derivative encoding $LysO_{CL}$ with the L288C substitution.
pHYD6231	A pHYD5580 derivative encoding $LysO_{CL}$ with the V290C substitution.
pHYD6439	A pHYD5580 derivative encoding $LysO_{CL}$ with the L293C substitution.
pHYD6226	A pHYD5580 derivative encoding $LysO_{CL}$ with the A295C substitution.
pHYD5517	Derivative of the plasmid pHYD5537, bearing the *phoA* gene encoding alkaline phosphatase (PhoA) lacking its signal sequence (lacking the N-terminal 26 amino acids). The corresponding gene is present between the SalI and HindIII sites.
pHYD6282	Derivative of the plasmid pHYD5517, encoding the 52F $LysO_{N\text{-}HA}$-PhoA hybrid with the PhoA moiety present after amino acid 52 of LysO.
pHYD6283	Derivative of the plasmid pHYD5517, encoding the 61F $LysO_{N\text{-}HA}$-PhoA hybrid with the PhoA moiety present after amino acid 61 of LysO.

pHYD6284	Derivative of the plasmid pHYD5517, encoding the 86F LysO$_{\text{N-HA}}$-PhoA hybrid with the PhoA moiety present after amino acid 86 of LysO.
pHYD6285	Derivative of the plasmid pHYD5517, encoding the 95F LysO$_{\text{N-HA}}$-PhoA hybrid with the PhoA moiety present after amino acid 95 of LysO.
pHYD6286	Derivative of the plasmid pHYD5517, encoding the 109F LysO$_{\text{N-HA}}$-PhoA hybrid with the PhoA moiety present after amino acid 109 of LysO.
pHYD6287	Derivative of the plasmid pHYD5517, encoding the 111F LysO$_{\text{N-HA}}$-PhoA hybrid with the PhoA moiety present after amino acid 111 of LysO.
pHYD6288	Derivative of the plasmid pHYD5517, encoding the 135F LysO$_{\text{N-HA}}$-PhoA hybrid with the PhoA moiety present after amino acid 135 of LysO.
pHYD6289	Derivative of the plasmid pHYD5517, encoding the 160F LysO$_{\text{N-HA}}$-PhoA hybrid with the PhoA moiety present after amino acid 160 of LysO.
pHYD6290	Derivative of the plasmid pHYD5517, encoding the 163F LysO$_{\text{N-HA}}$-PhoA hybrid with the PhoA moiety present after amino acid 163 of LysO.
pHYD6291	Derivative of the plasmid pHYD5517, encoding the 191F LysO$_{\text{N-HA}}$-PhoA hybrid with the PhoA moiety present after amino acid 191 of LysO.
pHYD6292	Derivative of the plasmid pHYD5517, encoding the 198F

	$LysO_{N-HA}$-PhoA hybrid with the PhoA moiety present after amino acid 198 of LysO.
pHYD6293	Derivative of the plasmid pHYD5517, encoding the 202F $LysO_{N-HA}$-PhoA hybrid with the PhoA moiety present after amino acid 202 of LysO.
pHYD6294	Derivative of the plasmid pHYD5517, encoding the 234F $LysO_{N-HA}$-PhoA hybrid with the PhoA moiety present after amino acid 234 of LysO.
pHYD6295	Derivative of the plasmid pHYD5517, encoding the 260F $LysO_{N-HA}$-PhoA hybrid with the PhoA moiety present after amino acid 260 of LysO.
pHYD6296	Derivative of the plasmid pHYD5517, encoding the 273F $LysO_{N-HA}$-PhoA hybrid with the PhoA moiety present after amino acid 273 of LysO.
pHYD6297	Derivative of the plasmid pHYD5517, encoding the 298F $LysO_{N-HA}$-PhoA hybrid with the PhoA moiety present after amino acid 298 of LysO.
pHYD6246	A pHYD5579 derivative encoding LysO with the E84Q substitution.
pHYD6247	A pHYD5579 derivative encoding LysO with the E96Q substitution.
pHYD6248	A pHYD5579 derivative encoding LysO with the E107Q substitution.
pHYD6249	A pHYD5579 derivative encoding LysO with the E134Q substitution.

pHYD6250	A pHYD5579 derivative encoding LysO with the E137Q substitution.
pHYD6251	A pHYD5579 derivative encoding LysO with the E215Q substitution.
pHYD6252	A pHYD5579 derivative encoding LysO with the E233Q substitution.
pHYD6253	A pHYD5579 derivative encoding LysO with the D52N substitution.
pHYD6254	A pHYD5579 derivative encoding LysO with the D190N substitution.
pHYD6255	A pHYD5579 derivative encoding LysO with the D229N substitution.
pHYD6256	A pHYD5579 derivative encoding LysO with the D261N substitution.
pHYD6257	A pHYD5579 derivative encoding LysO with the D274N substitution.
pBAD18	Plasmid for L-arabinose mediated expression of gene cloned under the transcriptional control of P_{ara} promoter.
pHYD3011	A modified version of pBAD18, in which a pre-existing NdeI restriction site in the vector backbone eliminated by end filling and the multiple cloning site (MCS) of pET21b was placed downstream of the L-arabinose inducible P_{ara} promoter. In addition, pHYD3011 contains Tn*10*dCm element, from the lambda phage λNK1324 present in the BamHI site of its MCS.

pHYD6240	Derivative of the plasmid pHYD3011, encoding $LysO_{N\text{-}HA}$ with the corresponding gene, cloned in NdeI and HindIII sites.
pHYD6401	Derivative of the plasmid pHYD6240, encoding LysO containing the D261N substitution.
pHYD6402	Derivative of the plasmid pHYD6240, encoding LysO containing the E233Q substitution.
PHYD2836	Derivative of pTrc99A encoding LysO expressed from the P_{trc} promoter, *lysO* is present in the NdeI and HindIII sites.
pHYD2868	Derivative of pTrc99A, encoding $MscL_{C\text{-}HA}$ expressed from the P_{trc} promoter, the corresponding gene, is present in the NdeI and SalI sites.

The plasmids pHYD2836, pTrc99A, pHYD5001, pBAD18, and pHYD2868 are described in references 7, 64, 65, 67 and 39 respectively. The lambda phage λNK1324 is described in reference 66.

Table S3 Oligonucleotide primers used in this study

Primer ID	Sequence (5'-3')	Destination plasmid
JGAALYSONHAFP	GAATTCCATATGTACCCATACGATGTTC CTGACTATGCGGGCGGCCCATTTTCTG GGCTGTT	pHYD5579
JGAALYSOCYS1FP	ATTACCGTTATTTTACTGGCCAATATTG CCGCCCTGATG	pHYD5580
JGAALYSOCYS1RP	CATCAGGGCGGCAATATTGGCCAGTAA AATAACGGTAAT	pHYD5580

JGAALYSOCYS2FP	CTGGAGTCGCTAAAACTGGCCGGCGTA GTAGTGATTGGT	pHYD5580
JGAALYSOCYS2RP	ACCAATCACTACTACGCCGGCCAGTTT TAGCGACTCCAG	pHYD5580
JGAALYSOCYS3FP	TCTACTGCACTGGGCTTAGCCGGTGCC ACATCAATGGAT	pHYD5580
JGAALYSOCYS3RP	ATCCATTGATGTGGCACCGGCTAAGCC CAGTGCAGTAGA	pHYD5580
JGAAF2CFP	GACTATGCGGGCGGCCCATGCTCTGG GCTGTTAATCATTC	pHYD6229
JGAAF2CRP	GAATGATTAACAGCCCAGAGCATGGGC CGCCCGCATAGTC	pHYD6229
JGAALY7CSFPn	GAATTCCATATGTTTTCTGGGCTGTTAT GCATTCTGGTTCCCCTG	pHYD6266
JGAALY10CSFP1	GCTGTTAATCATTCTGTGCCCCCTGATT GTGG	pHYD6267
JGAALY10CSFP2	CCACAATCAGGGGGCACAGAATGATTA ACAGC	pHYD6267
JGAALYV14CFP	CTGGTTCCCCTGATTTGCGGTTACCTC ATTCCG	pHYD6268
JGAALYV14RP	CGGAATGAGGTAACCGCAAATCAGGG GAACCAG	pHYD6268
JGAALysOG15CFP	GGTTCCCCTGATTGTGTGCTACCTCATT CCGCTTC	pHYD6434

JGAALysOG15CRP	GAAGCGGAATGAGGTAGCACACAATCA GGGGAACC	pHYD6434
JGAAI18CFP	GATTGTGGGTTACCTCTGCCCGCTTCG CCAACAAG	pHYD6210
JGAAI18CRP	CTTGTTGGCGAAGCGGGCAGAGGTAA CCCACAATC	pHYD6210
JGAAQ22CFP	CCTCATTCCGCTTCGCTGCCAAGCTGC GTTAAAAG	pHYD6259
JGAAQ22CRP	CTTTTAACGCAGCTTGGCAGCGAAGCG GAATGAGG	pHYD6259
JGAAI29CFP	CAACAAGCTGCGTTAAAAGTTTGCAATC AGCTATTAAGCTGGATG	pHYD6422
JGAAI29CRP	CATCCAGCTTAATAGCTGATTGCAAACT TTTAACGCAGCTTGTTG	pHYD6422
JGAAL32CFP	GTTATTAATCAGTGCTTAAGCTGGATG	pHYD6211
JGAAL32CRP	CATCCAGCTTAAGCACTGATTAATAAC	pHYD6211
JGAALysOM36CFP	CAGCTATTAAGCTGGTGCGTTTACCTTA TTCTCTTTTTTATGGG	pHYD6457
JGAALysOM36CRP	CCCATAAAAAAGAGAATAAGGTAAACG CACCAGCTTAATAGCTG	pHYD6457
JGAALYL39CFP	AGCTGGATGGTTTACTGCATTCTCTTTT TTATG	pHYD6212
JGAALYL39CRP	CATAAAAAAGAGAATGCAGTAAACCATC CAGCT	pHYD6212

JGAAF43CFP	CCTTATTCTCTTTTGCATGGGTATCAGTC	pHYD6213
JGAAF43CRP	GACTGATACCCATGCAAAAGAGAATAAGG	pHYD6213
JGAALysOA49CFP	TTATGGGTATCAGTCTGTGCTTTCTCGATAACCTCGCC	pHYD6435
JGAALysOA49CRP	GGCGAGGTTATCGAGAAAGCACAGACTGATACCCATAA	pHYD6435
JGAAA55CFP	GTTTCTCGATAACCTCTGCAGTAACCTGTTGGCGATT	pHYD6230
JGAAA55CRP	AATCGCCAACAGGTTACTGCAGAGGTTATCGAGAAAC	pHYD6230
JGAAA60CFP	CAGTAACCTGTTGTGCATTCTGCATTATTC	pHYD6214
JGAAA60CRP	GAATAATGCAGAATGCACAACAGGTTACTG	pHYD6214
JGAAH63CFP	CTGTTGGCGATTCTGTGCTATTCTGCCGTCAGTATT	pHYD6215
JGAAH63CRP	AATACTGACGGCAGAATAGCACAGAATCGCCAACAG	pHYD6215
JGAAV71CFP	GTCAGTATTACCTGCATTTTACTGTGT	pHYD6260
JGAAV71CRP	ACACAGTAAAATGCAGGTAATACTGAC	pHYD6260
JGAALysOL74CFP	GTCAGTATTACCGTTATTTTATGCGCCAATATTGCCGCCCTGATG	pHYD6458

JGAALysOL74CRP	CATCAGGGCGGCAATATTGGCGCATAA AATAACGGTAATACTGAC	pHYD6458
JGAAI77CFP	GTTATTTTACTGGCCAATTGCGCCGCC CTGATGTGGCTG	pHYD6405
JGAAI77CRP	CAGCCACATCAGGGCGGCGCAATTGG CCAGTAAAATAAC	pHYD6405
JGAAM81CFP	ATTGCCGCCCTGTGCTGGCTGGAGCG	pHYD6261
JGAAM81CRP	CGCTCCAGCCAGCACAGGGCGGCAAT	pHYD6261
JGAAH92CFP	CCTGCCGTGGCGCAACTGCCATCAGCA AGAAAAAC	pHYD6423
JGAAH92CRP	GTTTTTCTTGCTGATGGCAGTTGCGCC ACGGCAGG	pHYD6423
JGAAA105CFP	GTCGCGTATTGCGATGTGCCTGGAGTC GCTAAAAC	pHYD6424
JGAAA105CRP	GTTTTAGCGACTCCAGGCACATCGCAA TACGCGAC	pHYD6424
JGAAK110CFP	ATGGCGCTGGAGTCGCTATGCCTGTGC GGCGTAGTAGTGA	pHYD6425
JGAAK110CRP	TCACTACTACGCCGCACAGGCATAGCG ACTCCAGCGCCAT	pHYD6425
JGAAV116CFP	GCGGCGTAGTATGCATTGGTTTTGC	pHYD6262
JGAAV116CRP	GCAAAACCAATGCATACTACGCCGC	pHYD6262
JGAAA120CFP	GTAGTGATTGGTTTTTGCATTGGTCTAA GTGG	pHYD6216

JGAAA120CRP	CCACTTAGACCAATGCAAAAACCAATCA CTAC	pHYD6216
JGAALysOL126CFP	CCATTGGTCTAAGTGGATGCGCTTTCTT ACAACACGC	pHYD6436
JGAALysOL126CRP	GCGTGTTGTAAGAAAGCGCATCCACTT AGACCAATGG	pHYD6436
JGAAF128CFP	GTGGACTGGCTTGCTTACAACACGC	pHYD6217
JGAAF128CRP	GCGTGTTGTAAGCAAGCCAGTCCAC	pHYD6217
JGAAA132CFP	CTGGCTTTCTTACAACACTGCACCGAA GCCAGTGAAT	pHYD6218
JGAAA132CRP	ATTCACTGGCTTCGGTGCAGTGTTGTA AGAAAGCCAG	pHYD6218
JGAAL142CFP	CACGTTAATTTGCCTACTTTTCCTC	pHYD6219
JGAAL142CRP	GAGGAAAAGTAGGCAAATTAACGTG	pHYD6219
JGAAV147CFP	GCTACTTTTCCTCTGCGGTATTCAGTTG C	pHYD6407
JGAAV147CRP	GCAACTGAATACCGCAGAGGAAAAGTA GC	pHYD6407
JGAAR152CFP	CGTTGGTATTCAGTTGTGCAATAATGG CATGAC	pHYD6426
JGAAR152CRP	GTCATGCCATTATTGCACAACTGAATAC CAACG	pHYD6426
JGAAT157CFP	GCAATAATGGCATGTGCTTAAAGCAGA TTG	pHYD6220

JGAAT157CRP	CAATCTGCTTTAAGCACATGCCATTATTGC	pHYD6220
JGAAG167CFP	GTCCTTAATCGCCGGTGCATGATTGTCGCCGTG	pHYD6427
JGAAG167CRP	CACGGCGACAATCATGCACCGGCGATTAAGGAC	pHYD6427
JGAAV170CFP	CTTAATCGCCGGGGAATGATTTGCGCCGTGGTGGTGGTTGTCAG	pHYD6428
JGAAV170CRP	CTGACAACCACCACCACGGCGCAAATCATTCCCCGGCGATTAAG	pHYD6428
JGAAV172CFP	GAATGATTGTCGCCTGCGTGGTGGTTGTCAG	pHYD6263
JGAAV172CRP	CTGACAACCACCACGCAGGCGACAATCATTC	pHYD6263
JGAAV176CFP	CGCCGTGGTGGTGGTTTGCAGTTCATTAATTGG	pHYD6444
JGAAV176CRP	CCAATTAATGAACTGCAAACCACCACCACGGCG	pHYD6444
JGAAG182CFP	CATTAATTGGTTGCTTAATTAACGC	pHYD6445
JGAAG182CRP	GCGTTAATTAAGCAACCAATTAATG	pHYD6445
JGAAF187CFP	GTTTAATTAACGCCTGCATTCTTGATCTCCC	pHYD6221
JGAAF187CRP	GGGAGATCAAGAATGCAGGCGTTAATTAAAC	pHYD6221

JGAAL189CFP	GTTTAATTAACGCCTTTATTTGCGATCT CCCCATCAATACCGC	pHYD6406
JGAAL189CRP	GCGGTATTGATGGGGAGATCGCAAATA AAGGCGTTAATTAAAC	pHYD6406
JGAAI193CFP	ATTCTTGATCTCCCCTGCAATACCGCG CTGGCAATG	pHYD6269
JGAAI193CRP	CATTGCCAGCGCGGTATTGCAGGGGA GATCAAGAAT	pHYD6269
JGAAA196CFP	CATCAATACCTGCCTGGCAATGG	pHYD6222
JGAAA196CRP	CCATTGCCAGGCAGGTATTGATG	pHYD6222
JGAALysOA198CFP	CATCAATACCGCGCTGTGCATGGCCTC CGGTTTC	pHYD6459
JGAALysOA198CRP	GAAACCGGAGGCCATGCACAGCGCGG TATTGATG	pHYD6459
JGAAA200CFP	AATACCGCGCTGGCAATGTGCTCCGGT TTCGGCTGGT	pHYD6264
JGAAA200CRP	ACCAGCCGAAACCGGAGCACATTGCCA GCGCGGTATT	pHYD6264
JGAAG202CFP	CTGGCAATGGCCTCCTGCTTCGGCTGG TATTC	pHYD6409
JGAAG202CRP	GAATACCAGCCGAAGCAGGAGGCCATT GCCAG	pHYD6409
JGAAW205CFP	CCGGTTTCGGCTGCTATTCTCTTTCCG	pHYD6410
JGAAW205CRP	CGGAAAGAGAATAGCAGCCGAAACCG G	pHYD6410

JGAALysOS209CFP	GGCTGGTATTCTCTTTGCGGTATTTTAT TGACC	pHYD6460
JGAALysOS209CRP	GGTCAATAAAATACCGCAAAGAGAATA CCAGCC	pHYD6460
JGAAI211CFP	CTGGTATTCTCTTTCCGGTTGCTTATTG ACCGAATCTTTTGG	pHYD6411
JGAAI211CRP	CCAAAAGATTCGGTCAATAAGCAACCG GAAAGAGAATACCAG	pHYD6411
JGAAT214CFP	CGGTATTTTATTGTGCGAATCTTTTGGT C	pHYD6223
JGAAT214CRP	GACCAAAAGATTCGCACAATAAAATACC G	pHYD6223
JGAAS216CFP	GTATTTTATTGACCGAATGCTTTGGTCC GGTAATCGG	pHYD6446
JGAAS216CRP	CCGATTACCGGACCAAAGCATTCGGTC AATAAAATAC	pHYD6446
JGAAP219CFP	GACCGAATCTTTTGGTTGCGTAATCGG GAGCGCG	pHYD6447
JGAAP219CRP	CGCGCTCCCGATTACGCAACCAAAAGA TTCGGTC	pHYD6447
JGAAI221CFP	CTTTTGGTCCGGTATGCGGGAGCGCG GCG	pHYD6265
JGAAI221CRP	CGCCGCGCTCCCGCATACCGGACCAA AAG	pHYD6265

JGAALysOF226CFP	GTAATCGGGAGCGCGGCGTGCTTTAAT GATCTGGCCCGTG	pHYD6437
JGAALysOF226CRP	CACGGGCCAGATCATTAAAGCACGCCG CGCTCCCGATTAC	pHYD6437
JGAAD229CFP	CGCGGCGTTTTTTAATTGCCTGGCCCG TGAACTG	pHYD6412
JGAAD229CRP	CAGTTCACGGGCCAGGCAATTAAAAAA CGCCGCG	pHYD6412
JGAAA231CFP	GGCGTTTTTTAATGATCTGTGCCGTGAA CTGATTGCT	pHYD6224
JGAAA231CRP	AGCAATCAGTTCACGGCACAGATCATT AAAAAACGCC	pHYD6224
JGAALysOI235CFP	GATCTGGCCCGTGAACTGTGCGCTATT ATGTTGATCCC	pHYD6461
JGAALysOI235CRP	GGGATCAACATAATAGCGCACAGTTCA CGGGCCAGATC	pHYD6461
JGAAI237CFP	CCGTGAACTGATTGCTTGCATGTTGAT CCCTGGGC	pHYD6448
JGAAI237CRP	GCCCAGGGATCAACATGCAAGCAATCA GTTCACGG	pHYD6448
JGAAG242CFP	CTATTATGTTGATCCCTTGCCTGATTCG CCGCAGCC	pHYD6430
JGAAG242CRP	GGCTGCGGCGAATCAGGCAAGGGATC AACATAATAG	pHYD6430

JGAAR246CFP	CTGGGCTGATTCGCTGCAGCCGCTCTA CTGCAC	pHYD6408
JGAAR246CRP	GTGCAGTAGAGCGGCTGCAGCGAATC AGCCCAG	pHYD6408
JGAALysOR248CFP	CTGATTCGCCGCAGCTGCTCTACTGCA CTG	pHYD6438
JGAALysOR248CRP	CAGTGCAGTAGAGCAGCTGCGGCGAA TCAG	pHYD6438
JGAAT250CFP	GCTGATTCGCCGCAGCCGCTCTTGCGC ACTGGGCTTAGCC	pHYD6449
JGAAT250CRP	GGCTAAGCCCAGTGCGCAAGAGCGGC TGCGGCGAATCAGC	pHYD6449
JGAAG253CFP	CTCTACTGCACTGTGCTTAGCCGGTG	pHYD6431
JGAAG253CRP	CACCGGCTAAGCACAGTGCAGTAGAG	pHYD6431
JGAAL254CFP	CTCTACTGCACTGGGCTGCTGCGGTGC CACATCAATG	pHYD6432
JGAAL254CRP	CATTGATGTGGCACCGCAGCAGCCCAG TGCAGTAGAG	pHYD6432
JGAAT258CFP	GGCTTAGCCGGTGCCTGCTCAATGGAT TTCACC	pHYD6415
JGAAT258CRP	GGTGAAATCCATTGAGCAGGCACCGGC TAAGCC	pHYD6415
JGAAV266CFP	GATTTCACCCTGCCCTGCCTTCAACGT ACTGGC	pHYD6416

JGAAV266CRP	GCCAGTACGTTGAAGGCAGGGCAGGG TGAAATC	pHYD6416
JGAALYD274CFP	CGTACTGGCGGGCTGTGCATGGTCCC GGCGGCA	pHYD6270
JGAALYD274CRP	TGCCGCCGGGACCATGCACAGCCCGC CAGTACG	pHYD6270
JGAAI285CFP	CAATTGTTCACGGTTTTTGCCTTAGCCT GTTAGTGCCG	pHYD6417
JGAAI285CRP	CGGCACTAACAGGCTAAGGCAAAAACC GTGAACAATTG	pHYD6417
JGAAL286CFP	CAATTGTTCACGGTTTTATTTGCAGCCT GTTAGTGCCGATCC	pHYD6433
JGAAL286CRP	GGATCGGCACTAACAGGCTGCAAATAA AACCGTGAACAATTG	pHYD6433
JGAAL288CFP	CACGGTTTTATTCTTAGCTGCTTAGTGC CGATCCTCATC	pHYD6413
JGAAL288CRP	GATGAGGATCGGCACTAAGCAGCTAAG AATAAAACCGTG	pHYD6413
JGAAV290CFP	CTTAGCCTGTTATGCCCGATCCTCATC	pHYD6231
JGAAV290VRP	GATGAGGATCGGGCATAACAGGCTAAG	pHYD6231
JGAALysOL293CFP	CCTGTTAGTGCCGATCTGCATCGCCTT TTTCTC	pHYD6439
JGAALysOL293CRP	GAGAAAAAGGCGATGCAGATCGGCACT AACAGG	pHYD6439

JGAALY295CSRP	TAACCCAAGCTTCTACGCAGAGAAAAA GCAGATGAGGATCGGCAC	pHYD6226
JGAAPhosF	ATCCTCTAGAGTCGACCCTGTTCTGGA AAACCGGGC	pHYD5517
JGAAPhohR	CAAAACAGCCAAGCTTTTATTTCAGCCC CAGAGCGG	pHYD5517
JGAA52LysOSFP	CTGGCGTTTCTCGATGTCGACAACCTC GCCAGTAAC	pHYD6282
JGAA52LysOSRP	GTTACTGGCGAGGTTGTCGACATCGAG AAACGCCAG	pHYD6282
JGAALY61sRP	GAGGATGTCGACAATCGCCAACAGGTT ACTGGCGAG	pHYD6283
JGAALY86sRP	GAGGATGTCGACGCCTCGCTCCAGCC ACATCAGGGC	pHYD6284
JGAALY95sRPn	GAGGATGTCGACTTGCTGATGGTGGTT GCGCCACGG	pHYD6285
JGAALY109sRP	GAGGATGTCGACTAGCGACTCCAGCG CCATCGCAAT	pHYD6286
JGAALY111STRP	GAGGATGTCGACCAGTTTTAGCGACTC CAGCGCCAT	pHYD6287
JGAALY135sRP	GAGGATGTCGACGGCTTCGGTCGCGT GTTGTAAGAA	pHYD6288
JGAALY160sRP	GAGGATGTCGACCTGCTTTAAGGTCAT GCCATTATT	pHYD6289

JGAALY163sRP	GAGGATGTCGACAAGGACAATCTGCTTTAAGGTCAT	pHYD6290
JGAA191LYRn	GAGGATGTCGACGAGATCAAGAATAAAGGCG	pHYD6291
JGAALY198sRP	GAGGATGTCGACTGCCAGCGCGGTATTGATGGGGAG	pHYD6292
JGAA202LYRn	GAGGATGTCGACACCGGAGGCCATTGCCAGCGCGGT	pHYD6293
JGAALY234sRP	GAGGATGTCGACCAGTTCACGGGCCAGATCATTAAA	pHYD6294
JGAALY260sRP	GAGGATGTCGACCATTGATGTGGCACCGCATAAGCC	pHYD6295
JGAALY273sRP	GAGGATGTCGACCAGCCCGCCAGTACGTTGAAGAAC	pHYD6296
JGAALY298STRP	GAGGATGTCGACAGAGAAAAAGGCGATGAGGATC	pHYD6297
JGAAD52NFP	GTCTGGCGTTTCTCAACAACCTCGCCAG	pHYD6253
JGAAD52NRP	CTGGCGAGGTTGTTGAGAAACGCCAGAC	pHYD6253
JGAAD190NFP	GCCTTTATTCTTAACCTCCCCATC	pHYD6254
JGAAD190NRP	GATGGGGAGGTTAAGAATAAAGGC	pHYD6254
JGAAD229NFP	CGGCGTTTTTTAATAACCTGGCCCGTGAAC	pHYD6255

JGAAD229NRP	GTTCACGGGCCAGGTTATTAAAAAACGCCG	pHYD6255
JGAAD261NFP	GTGCCACATCAATGAACTTCACCCTGCCC	pHYD6256
JGAAD261NRP	GGGCAGGGTGAAGTTCATTGATGTGGCAC	pHYD6256
JGAAD274NFP	GTACTGGCGGGCTGAACATGGTCCCGGCGGC	pHYD6257
JGAAD274NRP	GCCGCCGGGACCATGTTCAGCCCGCCAGTAC	pHYD6257
JGAAE84QFP	GCCCTGATGTGGCTGCAGCGAGGCCTGCCGTGG	pHYD6246
JGAAE84QRP	CCACGGCAGGCCTCGCTGCAGCCACATCAGGGC	pHYD6246
JGAAE96QFP	GCGCAACCACCATCAGCAACAGAAACTCCCGTCGCGTATTG	pHYD6247
JGAAE96QRP	CAATACGCGACGGGAGTTTCTGTTGCTGATGGTGGTTGCGC	pHYD6247
JGAAE107QFP	GCGATGGCGCTGCAGTCGCTAAAAC	pHYD6248
JGAAE107QRP	GTTTTAGCGACTGCAGCGCCATCGC	pHYD6248
JGAAE134QFP	CTTACAACACGCGACCCAAGCCAGTGAATACACG	pHYD6249
JGAAE134QRP	CGTGTATTCACTGGCTTGGGTCGCGTGTTGTAAG	pHYD6249

JGAAE137QFP	CACGCGACCGAAGCCAGTCAATACACG TTAATTTTGCTAC	pHYD6250
JGAAE137QRP	GTAGCAAAATTAACGTGTATTGACTGG CTTCGGTCGCGTG	pHYD6250
JGAAE215QFP	CTTTCCGGTATTTTATTGACCCAATCTT TTGGTCCGGTAATCGG	pHYD6251
JGAAE215QRP	CCGATTACCGGACCAAAAGATTGGGTC AATAAAATACCGGAAAG	pHYD6251
JGAAE233QFP	GATCTGGCCCGTCAACTGATTGCTAT	pHYD6252
JGAAE233QRP	ATAGCAATCAGTTGACGGGCCAGATC	pHYD6252
JGSQPTRCFP[a]	CGACATCATAACGGTTCTGG	
JGSQPTRCRP[a]	TGGGACCACCGCGCTA	
JGSQPBADFP[b]	ATGCCATAGCATTTTTATCC	
JGSQPBADRP[b]	GATTTAATCTGTATCAGG	

Used for DNA sequencing of inserts placed under expression control of the P_{trc} promoter[a] and the P_{ara} promoter[b].

Table S4 Predicted transmembrane segments (TMS) and overall topology of LysO deduced from this study and its comparison with outputs from the indicated prediction tools.

Tools	TMS1	TMS2	TMS3	TMS4	TMS5	TMS6	6/7	TMS7	7/8	TMS8	TMS9	Overall Topology
TMHMM	4-20	32-51	61-83	111-133	138-155	167-189	-	204-226	247-269	276-298	-	N_{out}-C_{out}
SCAMPI	2-21	32-51	63-82	109-128	132-151	169-189	-	229-248	-	278-297	-	N_{out}-C_{out}
PHOBIUS	6-23	35-55	61-83	104-129	135-155	167-188	-	208-228	-	249-267	279-297	N_{out}-C_{in}
OCTOPUS	2-22	31-51	59-79	107-127	135-155	169-189	-	278-298	-	-	-	N_{out}-C_{in}
CCTOP	4-20	32-51	62-81	111-129	135-151	169-188	-	211-227	-	251-267	278-297	N_{out}-C_{in}
HMMTOP	2-20	33-51	65-83	111-129	140-158	169-187	-	209-227	-	249-267	278-296	N_{out}-C_{in}
MINNOU	1-11	25-50	54-85	105-128	135-152	165-188	-	193-203	-	221-240	270-299	N_{out}-C_{in}
MEMSAT-SVM	4-19	30-48	60-83	110-126	137-152	167-189	-	221-244	247-267	276-296	-	N_{out}-C_{out}
AlphaFold2	2-18	23-50	54-86	101-124	127-154	165-189	193-217	219-246	248-271	273-298	-	N_{out}-C_{out}
RoseTTA Fold	2-18	23-50	54-86	101-124	127-154	165-189	193-217	219-246	248-271	273-298	-	N_{out}-C_{out}
This study	2-20	30-52	63-83	100-124	131-153	168-189	193-217	219-246	247-268	276-299	-	N_{out}-C_{out}

TMHMM, SCAMPI, PHOBIUS, OCTOPUS, CCTOP, HMMTOP, MINNOU and MEMSAT-SVM are described in references 68, 44, 69, 70, 71, 72, 73 and 74 respectively. The topology of TMS7 and the two intramembrane segments in LysO (underlined) between TMS 6/7 (IR1) and TMS 7/8 (IR2) were assigned using the AlphaFold2 (25) and the RoseTTAFold (26) predictions.

Figure S1.

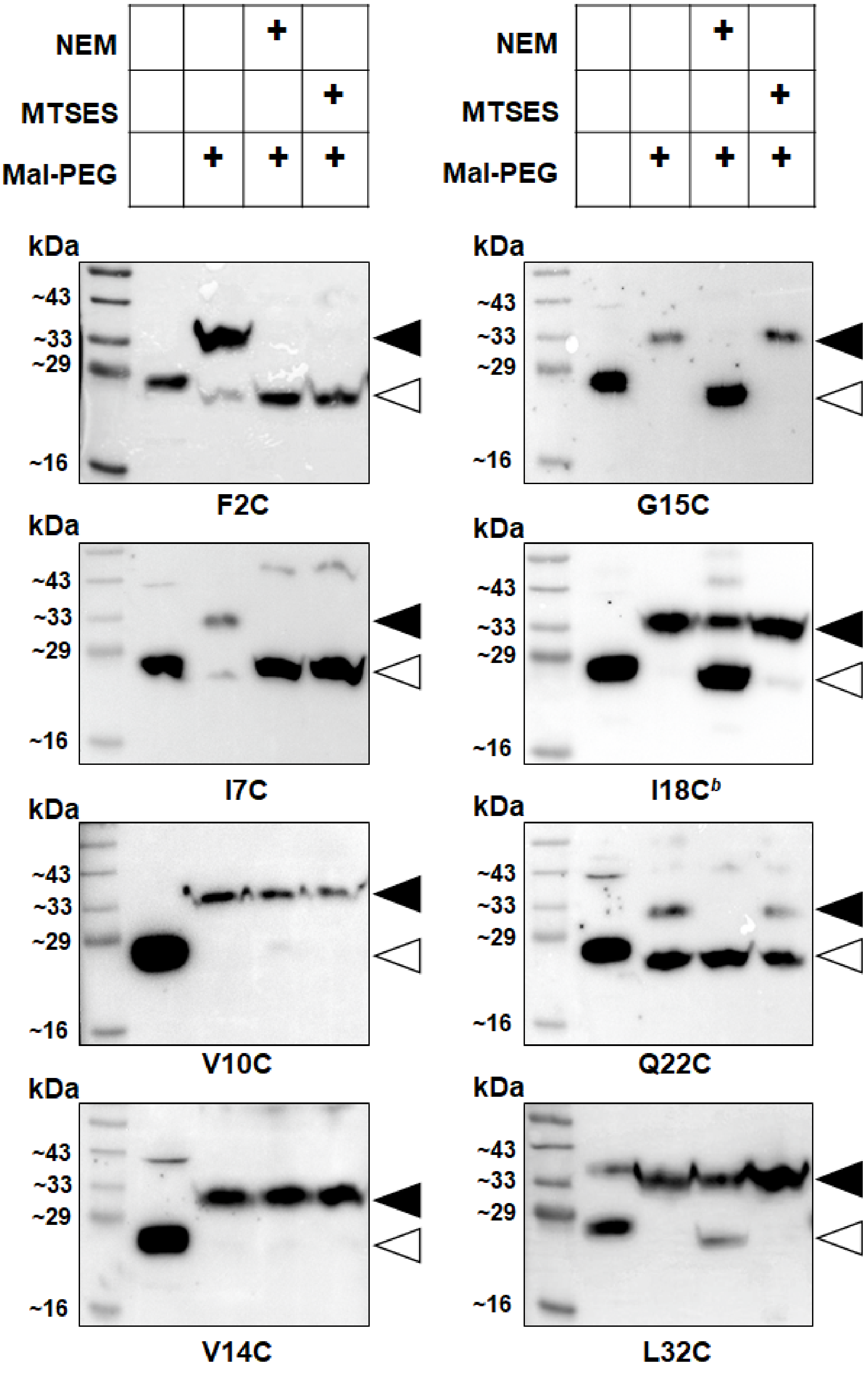
NEM
MTSES
Mal-PEG
+
+
+
+
+
kDa
~43
~33
~29
~16
F2C
G15C
I7C
I18C[b]
V10C
Q22C
V14C
L32C

Figure S1 (cont.)

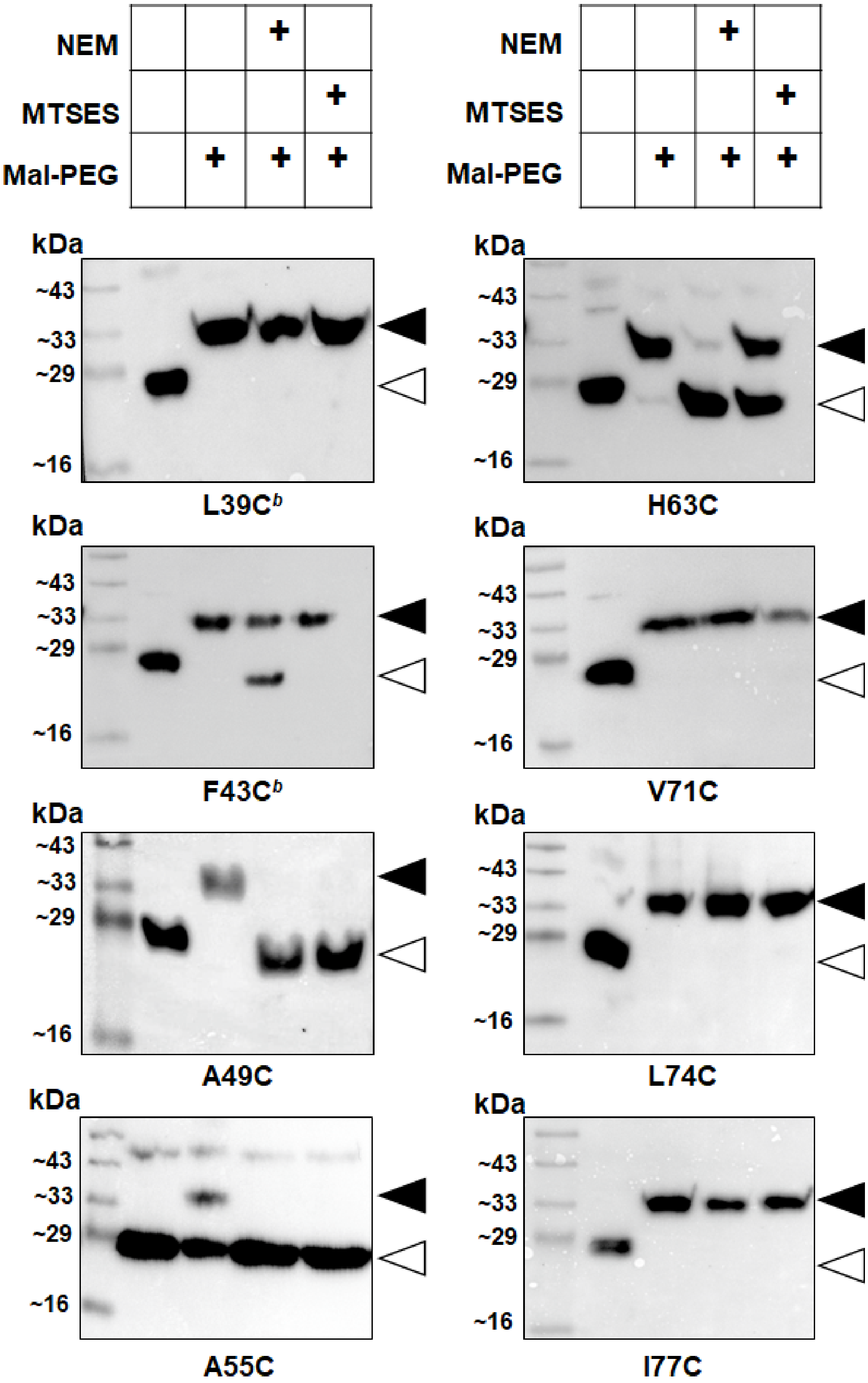

Figure S1 (cont.)

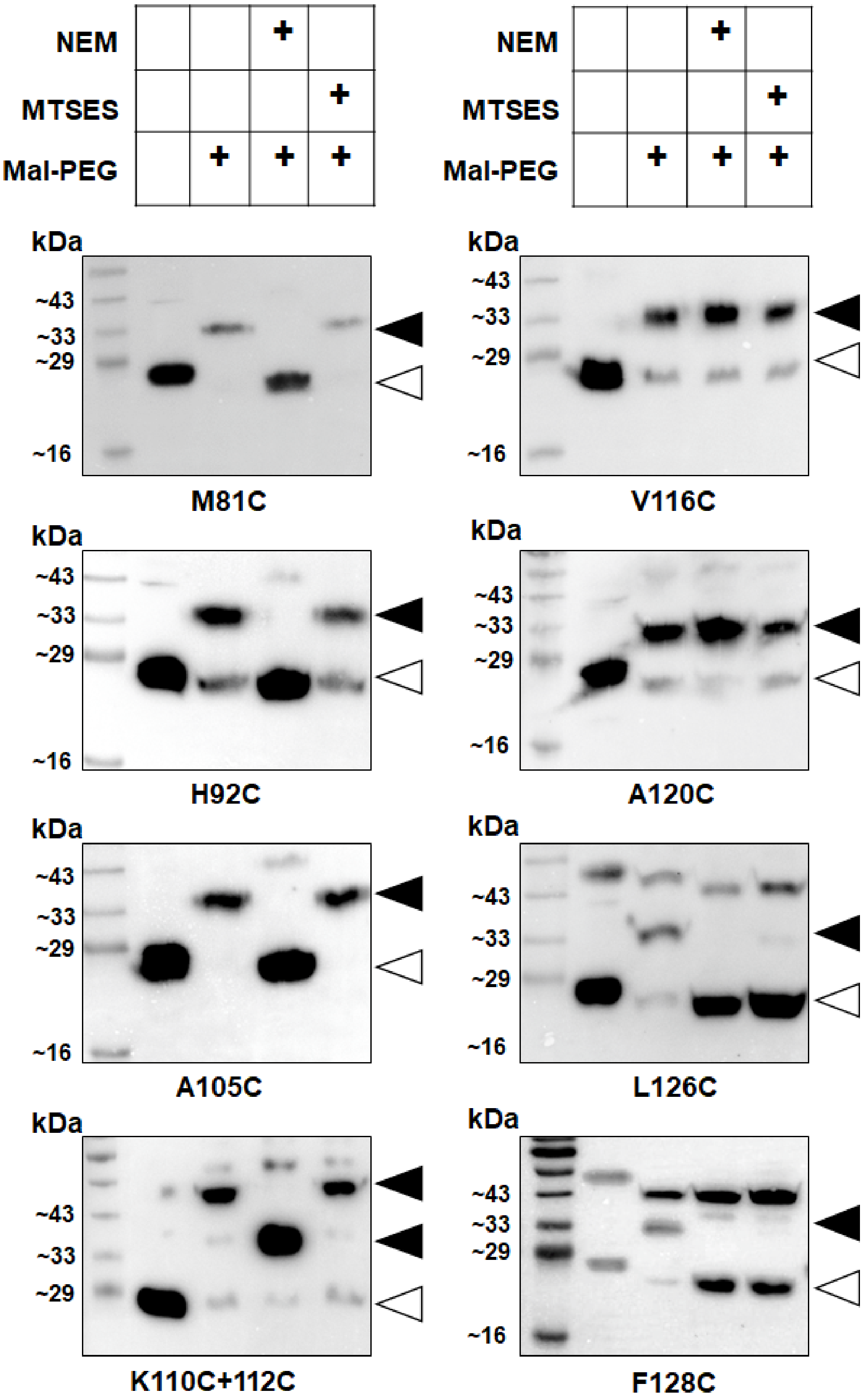

Figure S1 (cont.)

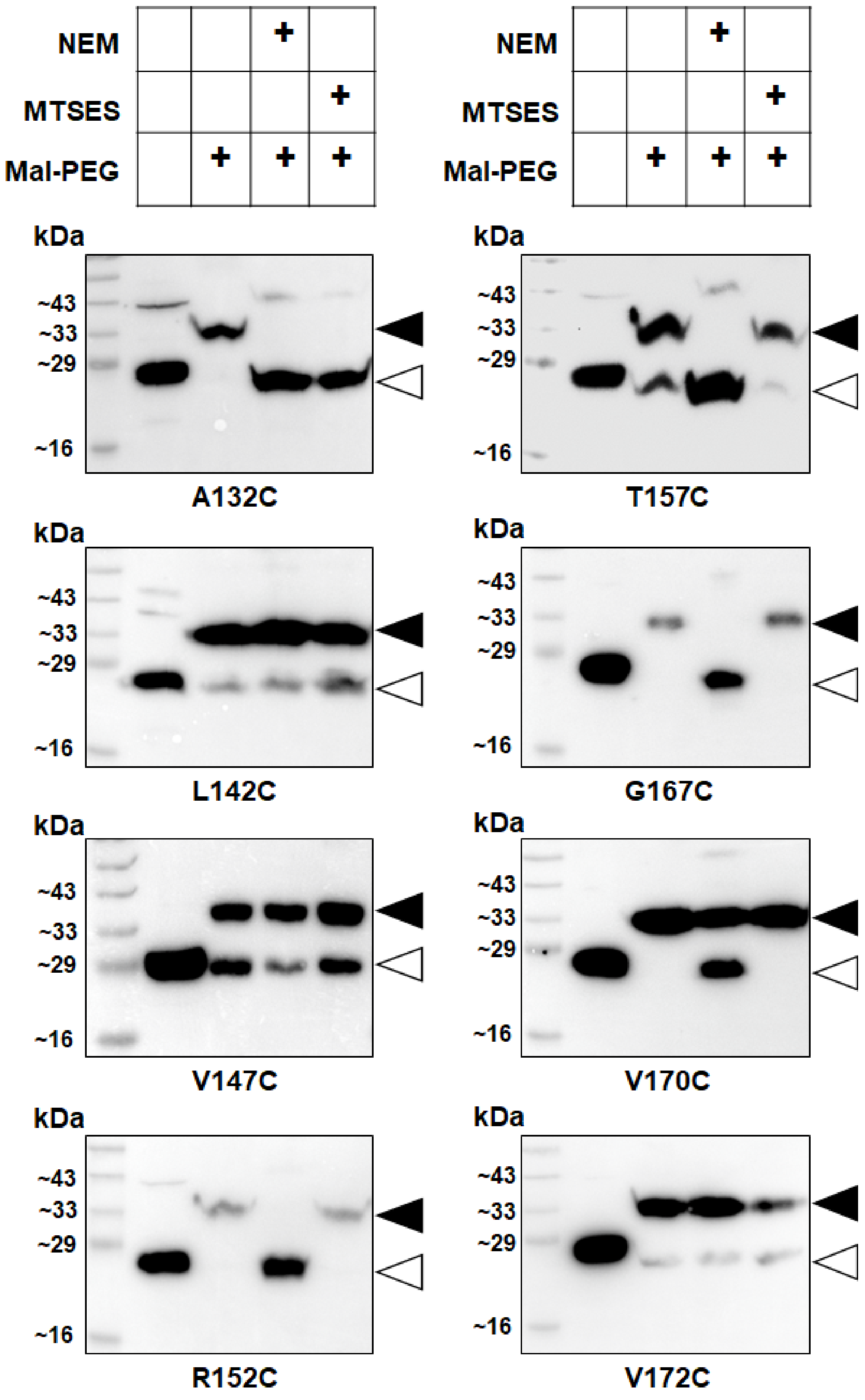

Figure S1 (cont.)

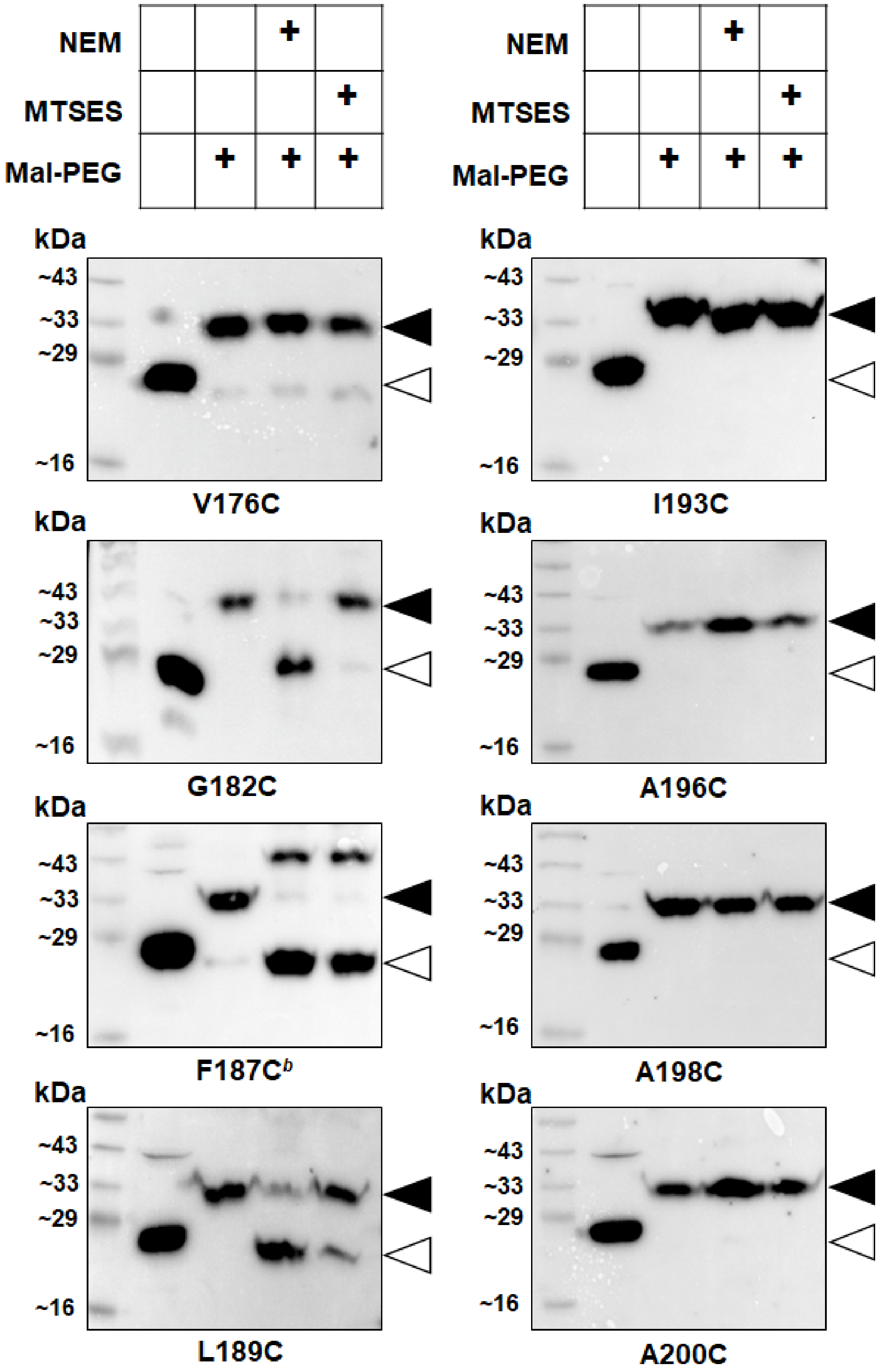

Figure S1 (cont.)

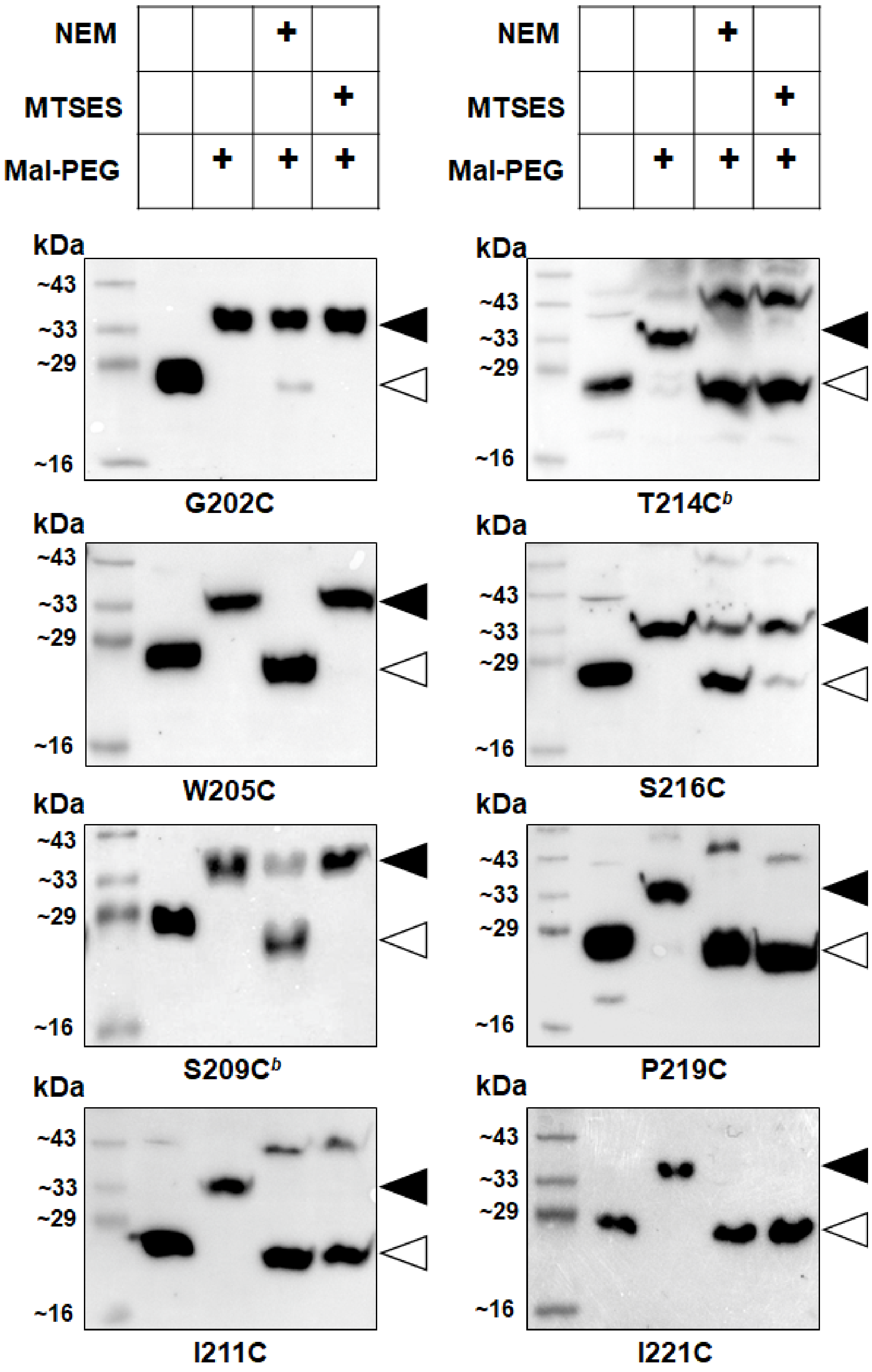

Figure S1 (cont.)

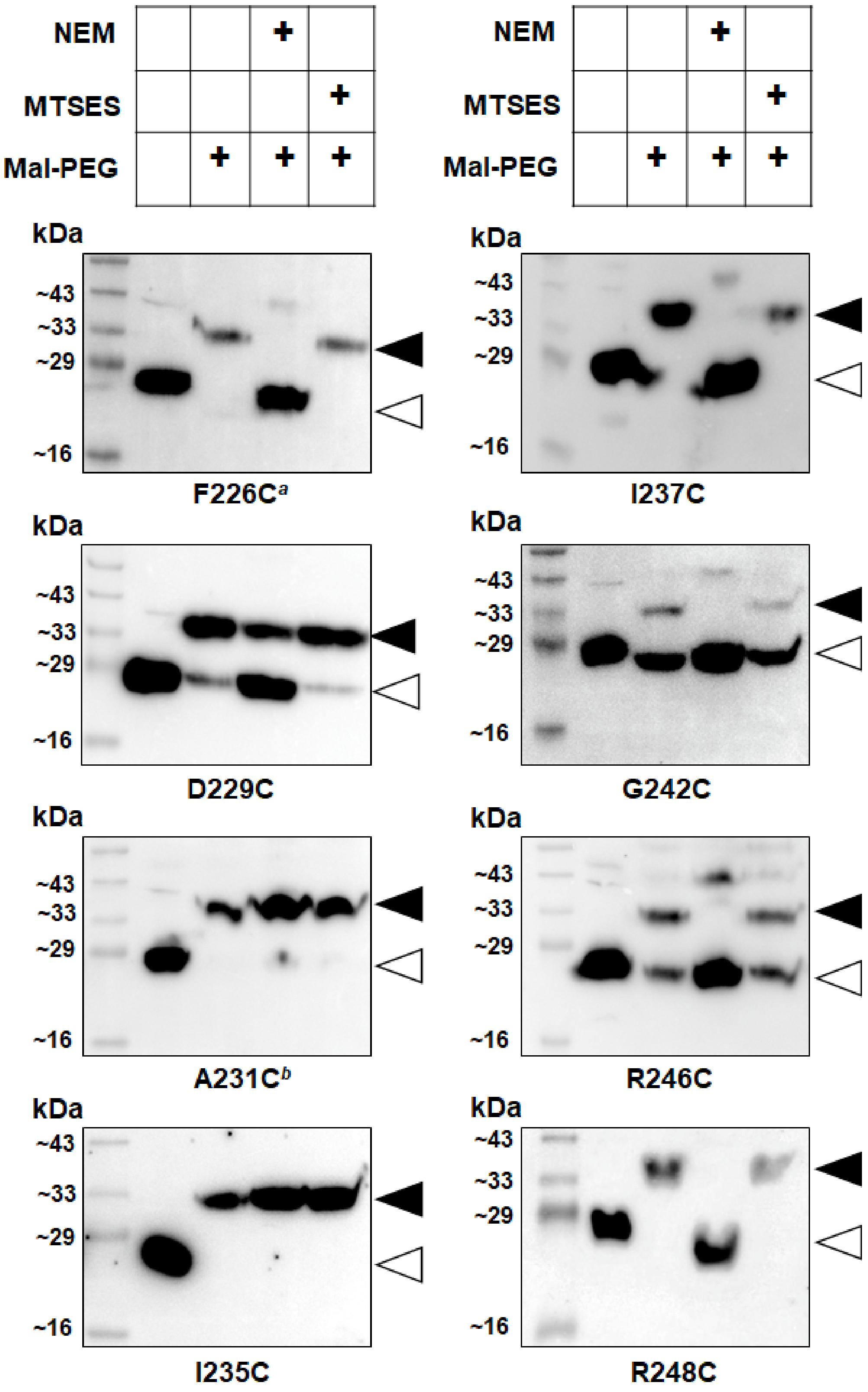

Figure S1 (cont.)

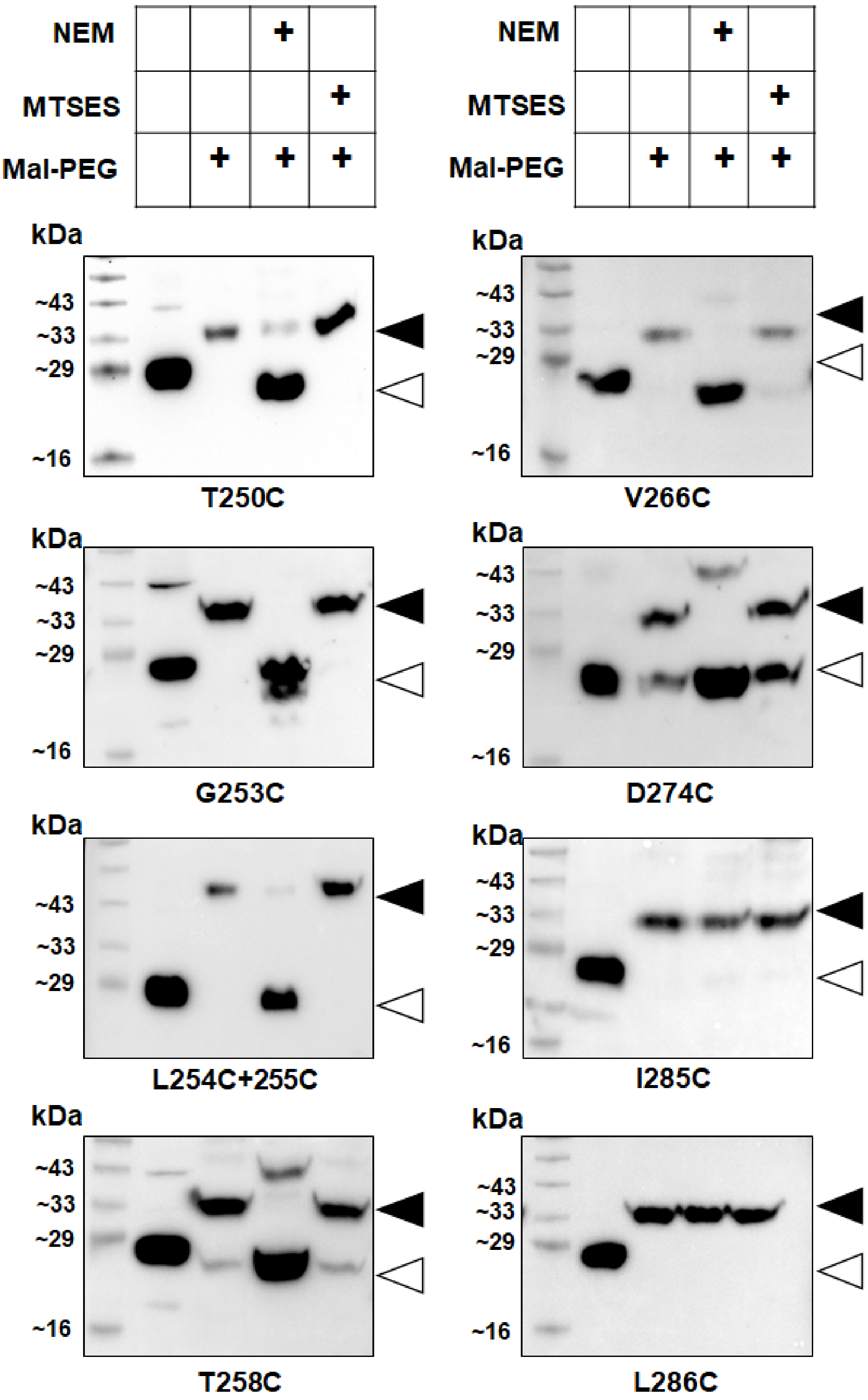

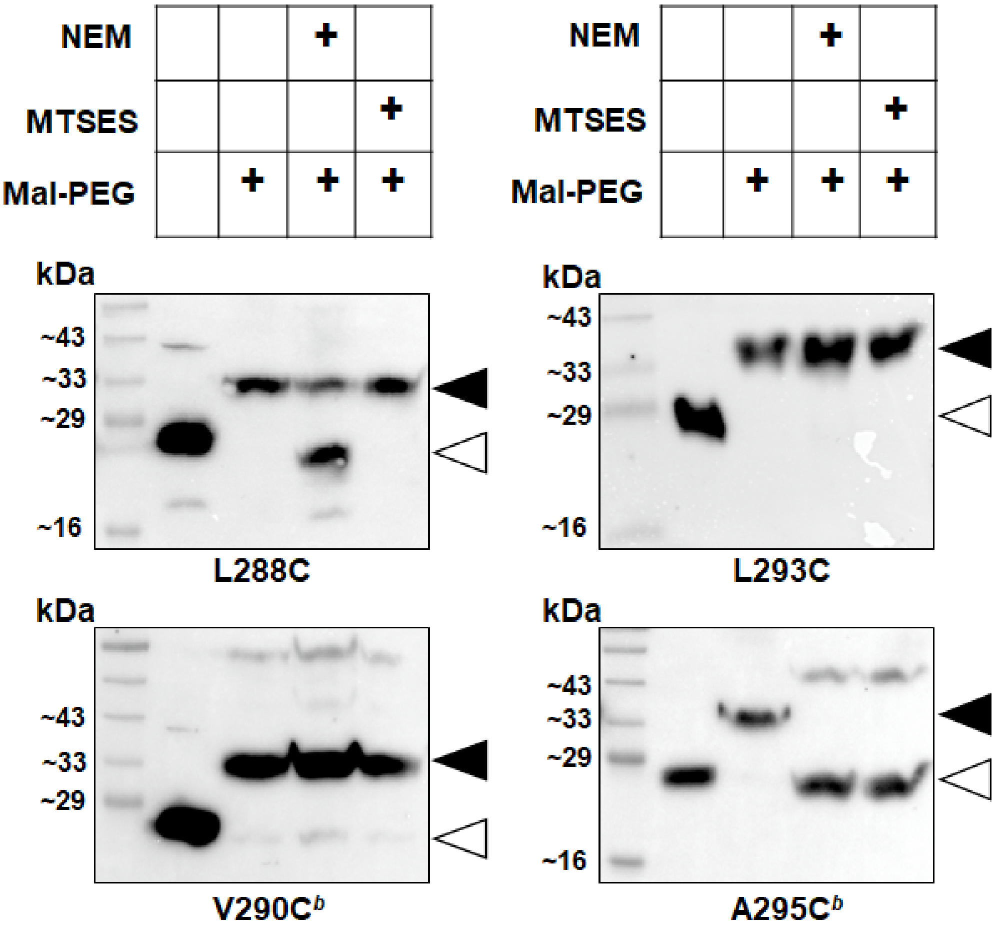

Figure S1. Anti-HA immunoblots depicting the results of the accessibility of the indicated cysteine substituted derivatives of the Cysless LysO$_{N\text{-}HA}$ (LysO$_{CL}$) to NEM and MTSES (method 1). Mid-log phase cultures of the strain GJ9026 bearing plasmids expressing the indicated monocysteine and two dicysteine derivatives (K110C, 112C and L254C, 255C) of LysO$_{CL}$, obtained after cultivation with 1 mM (or [a]10 µM, [b]100 µM) IPTG, were harvested and processed as described in experimental procedures. Sample treatments with NEM, MTSES and Mal-PEG are indicated. The positions of the free and the LysO:Mal-PEG adducts are indicated with open and filled triangles respectively. The indicated LysO proteins are expressed from the plasmid borne P$_{trc}$ promoter and plasmids for their expression are listed in Table S2.

Figure S2

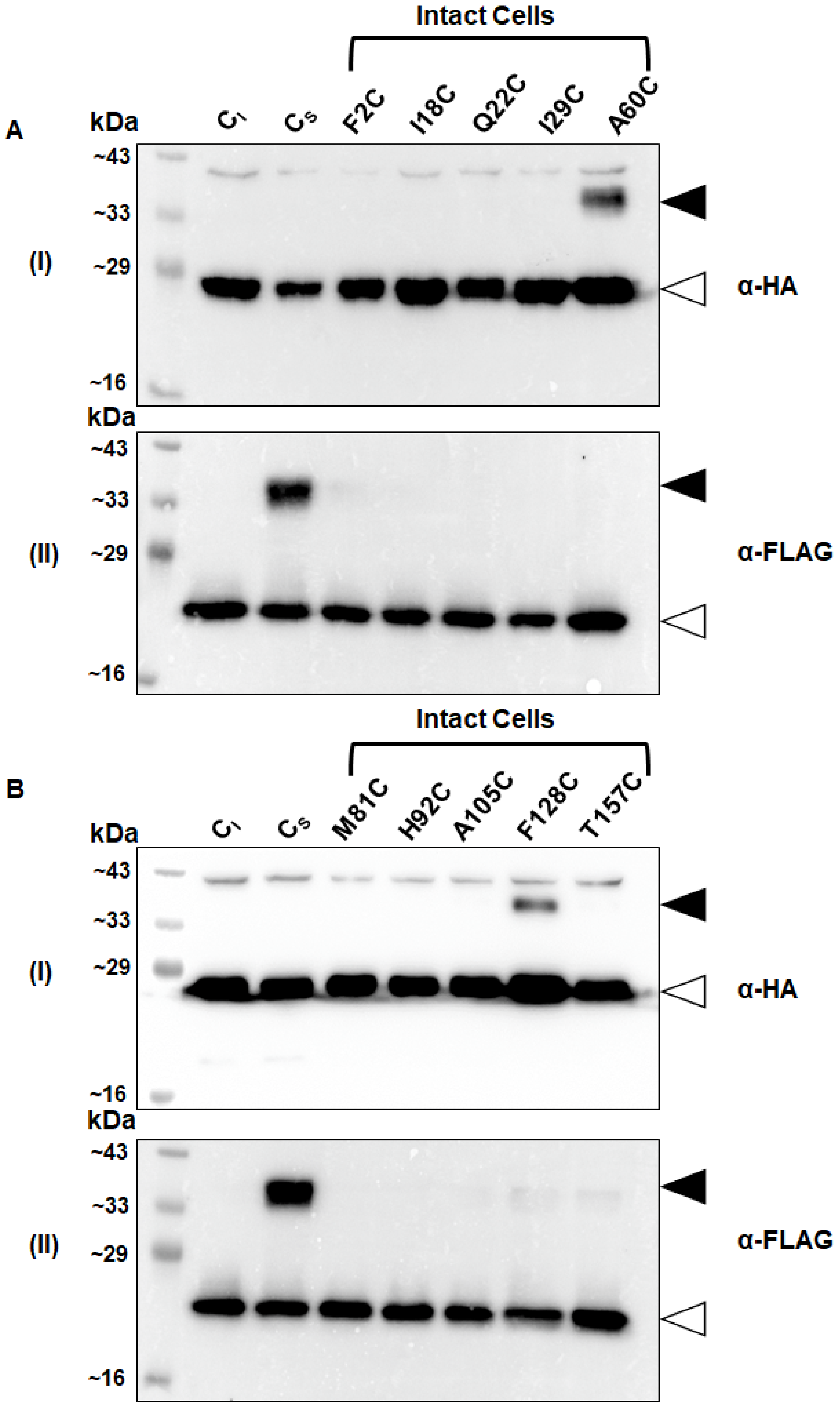
Intact Cells
A
kDa
C_I
C_S
F2C
I18C
Q22C
I29C
A60C
~43
~33
~29
~16
(I)
α-HA
(II)
α-FLAG
Intact Cells
B
M81C
H92C
A105C
F128C
T157C

Figure S2 (cont.)

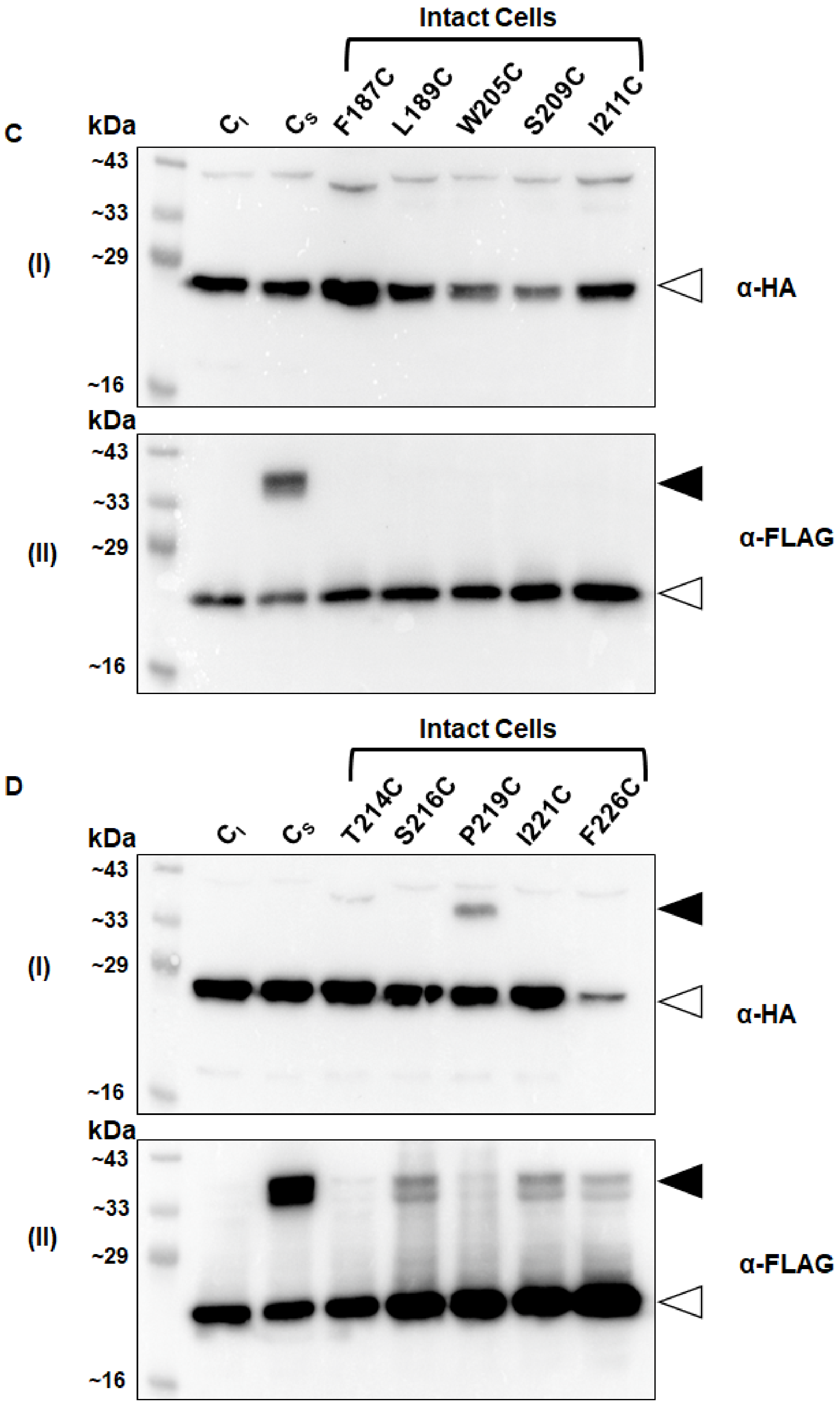

Figure S2 (cont.)

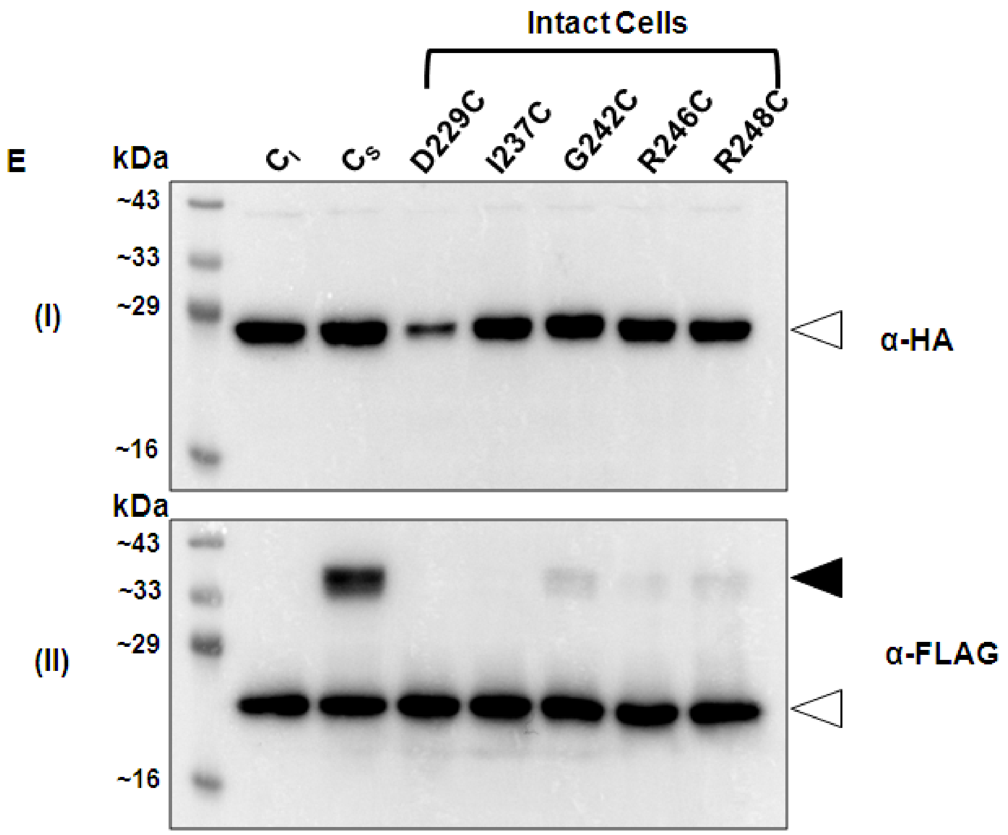

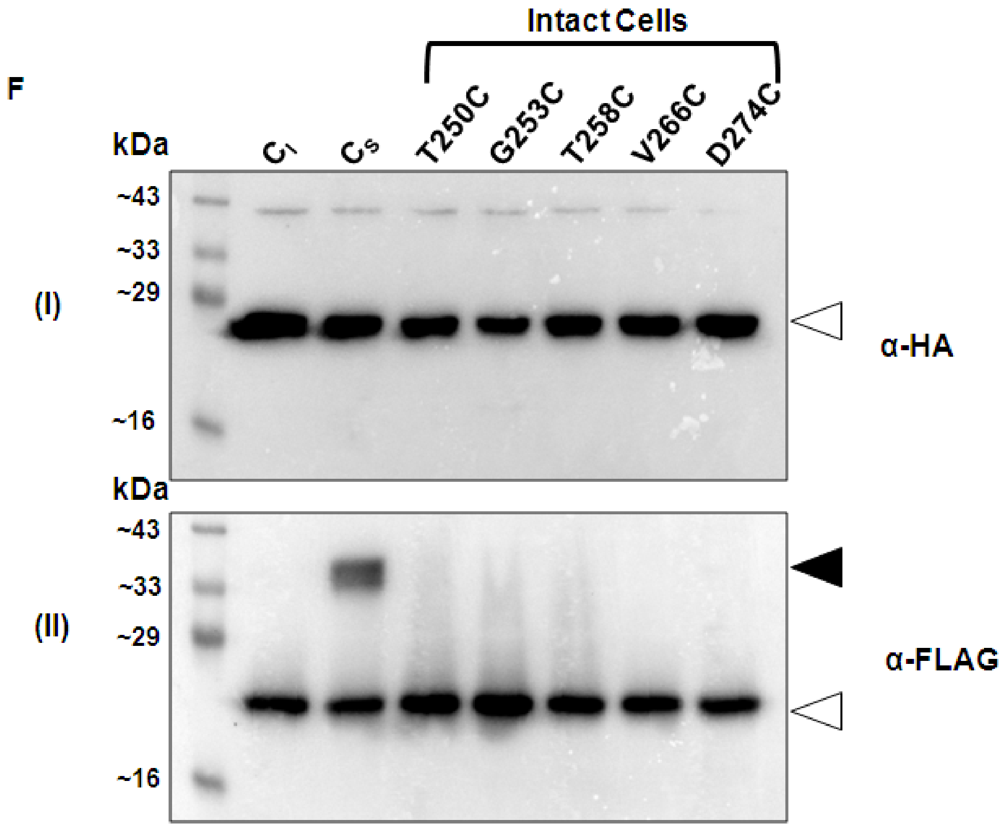

Figure S2. Immunoblots depicting results of cysteine accessibility *via* method 2 (panels A-F). Mid-exponential phase cultures of GJ16286 bearing plasmids encoding the indicated mono cysteine substituted derivatives of $LysO_{CL}$, were obtained after culturing in LB broth containing IPTG (IPTG concentration as per Figure S1 for a given sample). Cell densities equivalent to A_{600} of 1-2, were harvested by centrifugation, washed with 50 mM HEPES buffer (pH-6.5) and resuspended in 100 µl of the same buffer. Cell suspensions were treated with Mal-PEG (5 mM) and were rotated at low speed at room temperature in dark for 2 hours. Mal-PEG cross-linking was quenched by the addition of DTT (100 mM). Samples were solubilized in solution B (2% SDS 100 mM DTT) and briefly sonicated. Total protein of each sample was precipitated by the chloroform-methanol extraction method (62). Protein precipitates were solubilized in 60 µl of solution B. Following SDS sample buffer addition, equal volumes of protein samples were loaded into two 12% SDS-PAGE gels. The two transferred blots were individually probed with anti-HA (I) and anti-FLAG (II) antibodies. The positions of the free and Mal-PEG adducts of LysO and $PtsN^F$ (PtsN bearing a C-terminally abutted 3X FLAG tag) in both blots are indicated with open and filled triangles respectively. Two lanes in each panel labelled as C_I and C_S represent signals obtained from cells expressing the $LysO_{CL}$ (and $PtsN^F$) that were not sonicated (C_I) and sonicated (C_S), prior to labelling with Mal-PEG. Plasmids for Figure S2 are listed in Table S2.

Figure S3.

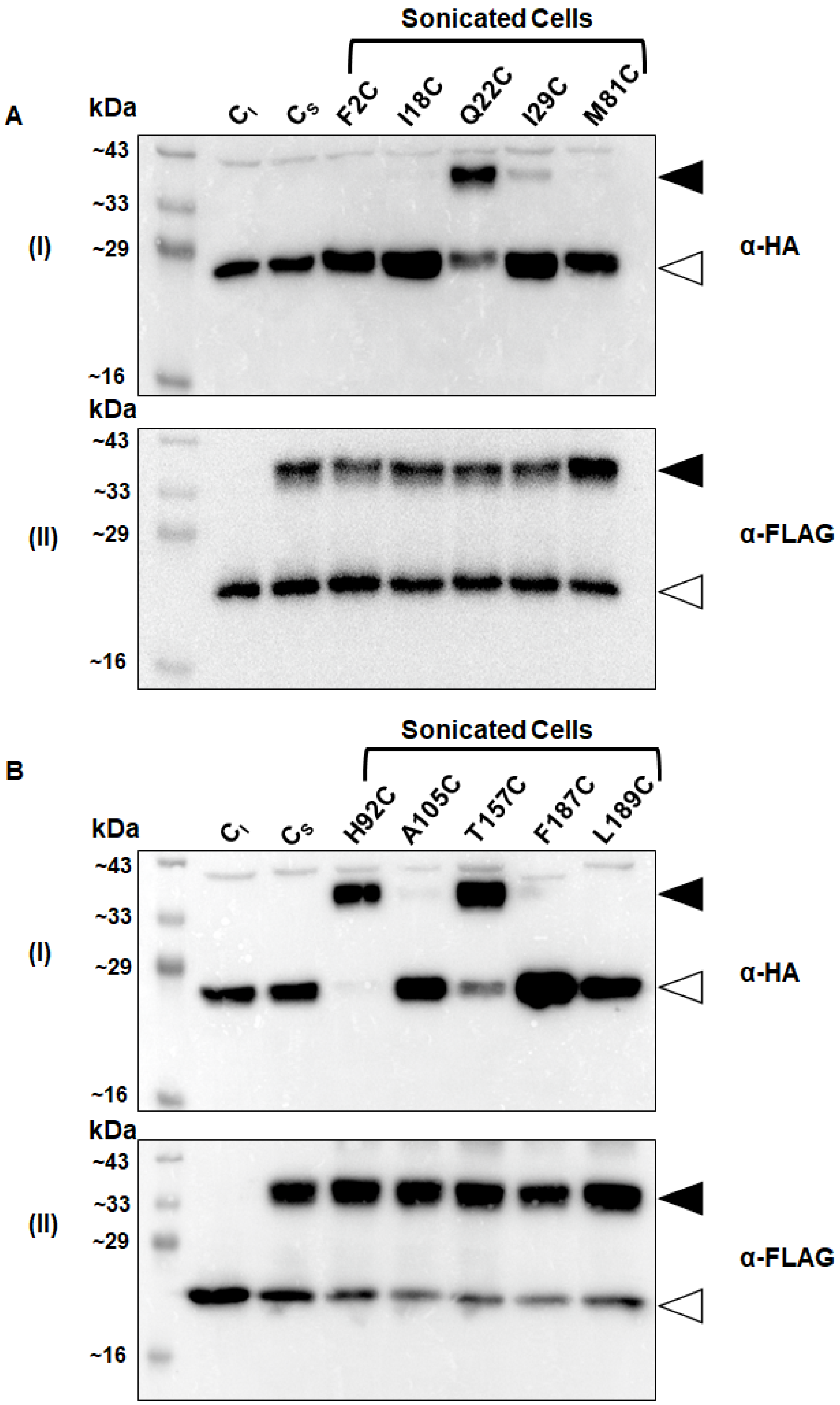
A
Sonicated Cells
kDa
C
C
F2C
I18C
Q22C
I29C
M81C
~43
~33
~29
~16
(I)
α-HA
kDa
~43
~33
~29
~16
(II)
α-FLAG
B
Sonicated Cells
kDa
C
C
H92C
A105C
T157C
F187C
L189C
~43
~33
~29
~16
(I)
α-HA
kDa
~43
~33
~29
~16
(II)
α-FLAG

Figure S3. (cont.)

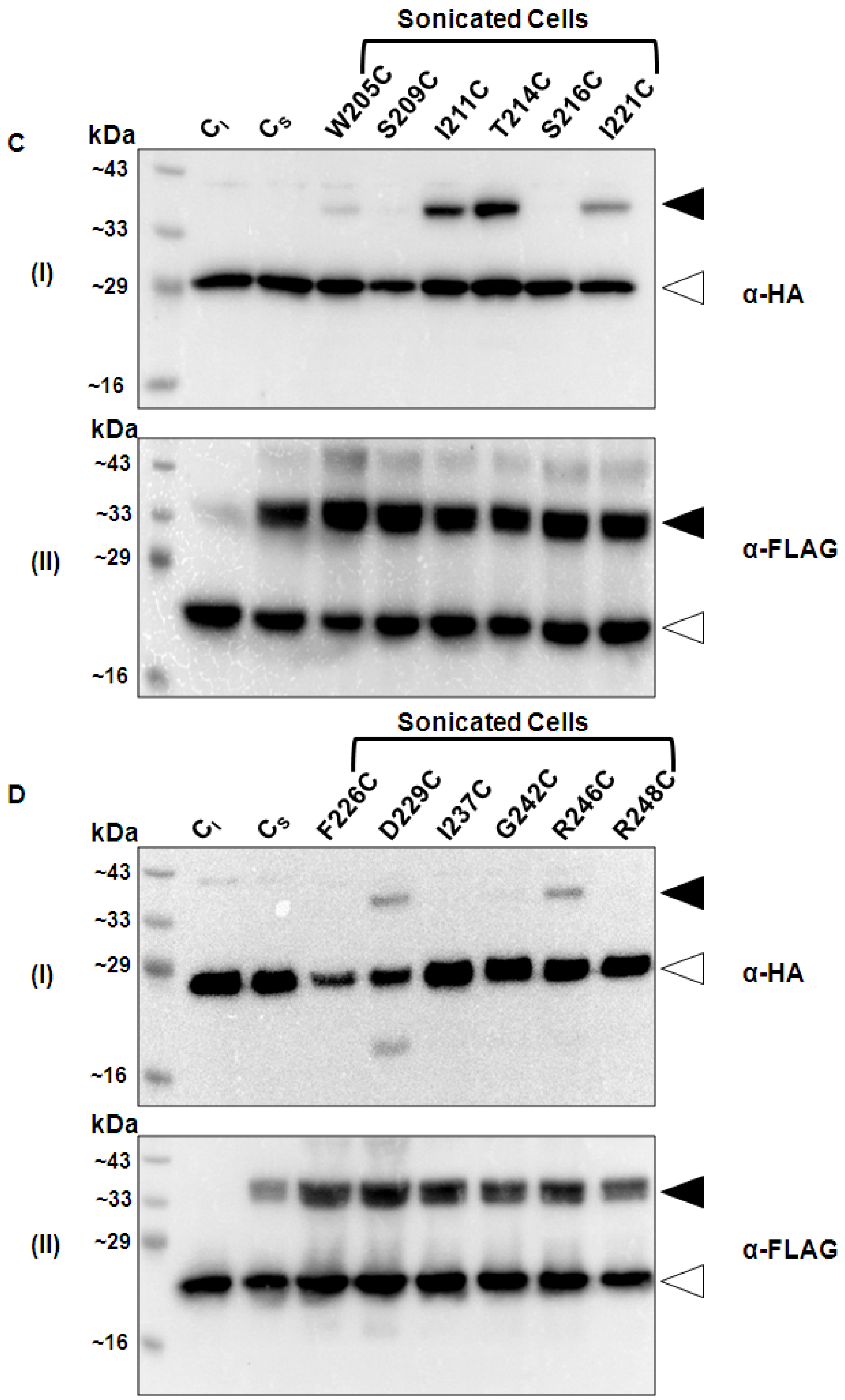

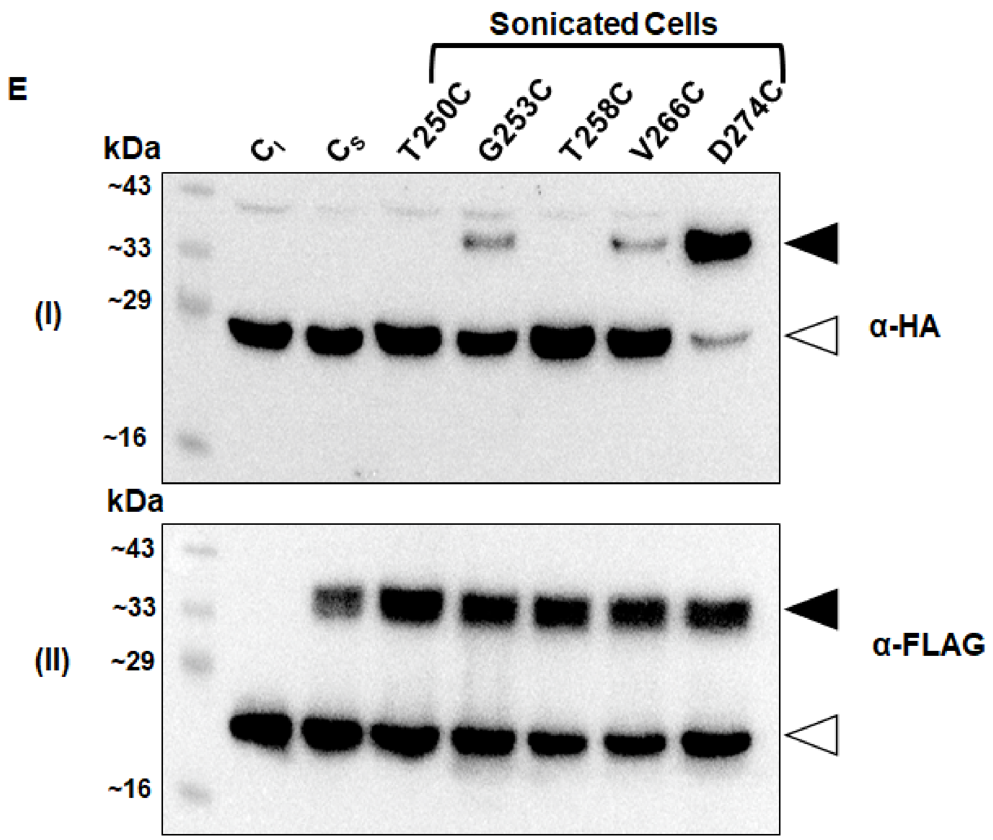

Figure S3. Immunoblots depicting results of Cys accessibility via method 2 (panels A-E). Growth conditions and other procedures were as described in Figure S2, except that cell suspensions were sonicated prior to labelling with Mal-PEG. Blots were individually probed with anti-HA (I) and anti-FLAG (II) antibodies. The positions of the free and the Mal-PEG adducts of LysO and PtsN^{F} are indicated with open and filled triangles respectively. Two lanes in each panel labelled as C_I and C_S represent signals obtained from cells expressing the LysO$_{CL}$ (and PtsN^{F}) that were not sonicated (C_I) and sonicated (C_S), prior to labelling with Mal-PEG.

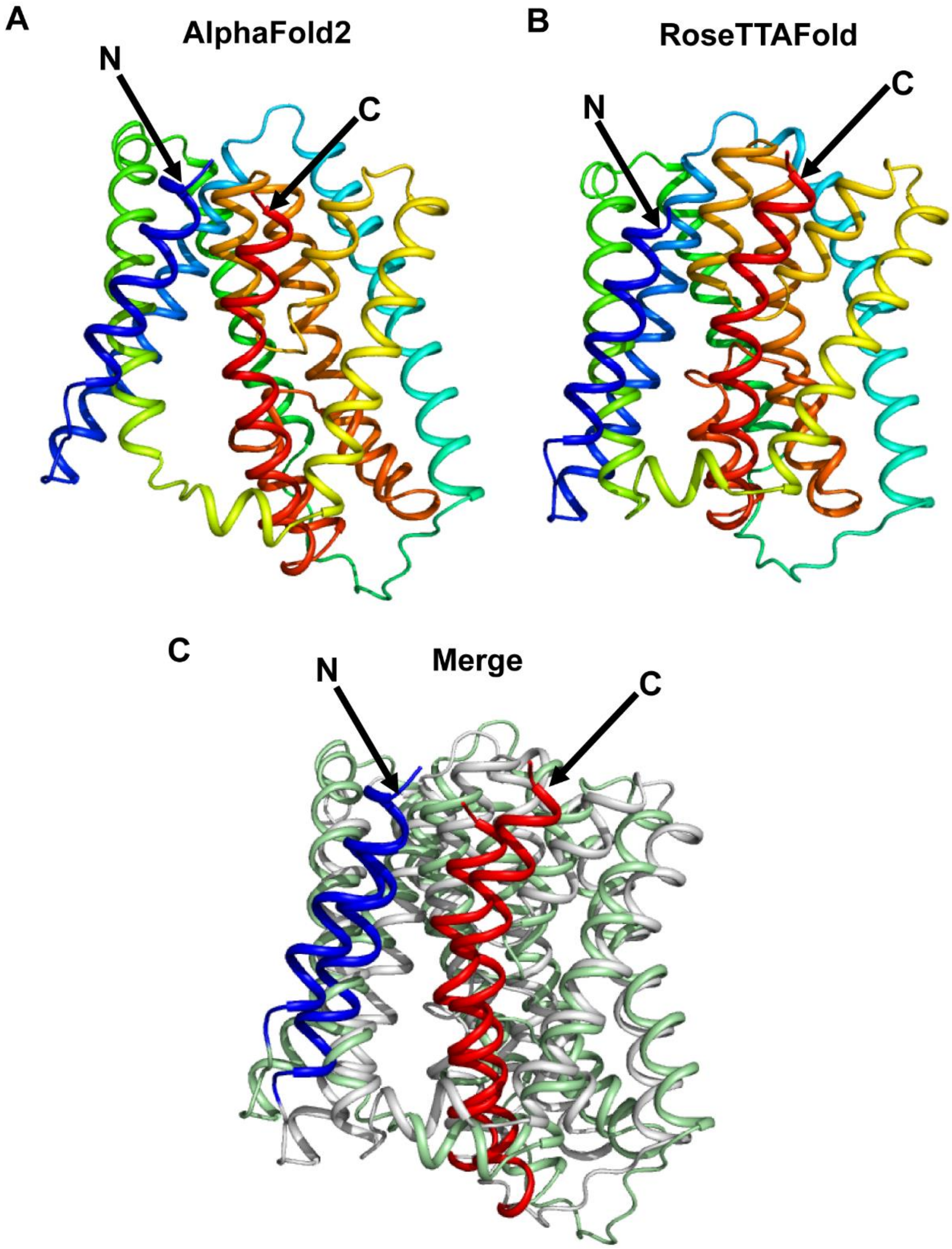

Figure S4. (A) AlphaFold2 (25) and (B) RoseTTAFold (26) predictions of LysO. Each transmembrane segment (TMS) is coloured differently using spectrum colour representation (blue to red) in PyMOL (Schrödinger, Inc). (C) Merge of the AlphaFold2 (light green) and RoseTTAFold (grey) models. The two models overlay with each other with an r.m.s.d of 4.3 for all the Cα atoms. Merging was visualized in PyMOL. TMS1 and TMS8 are coloured blue and red respectively and the N and C termini of LysO in TMS1 and TMS8 respectively are marked, in A, B and C.

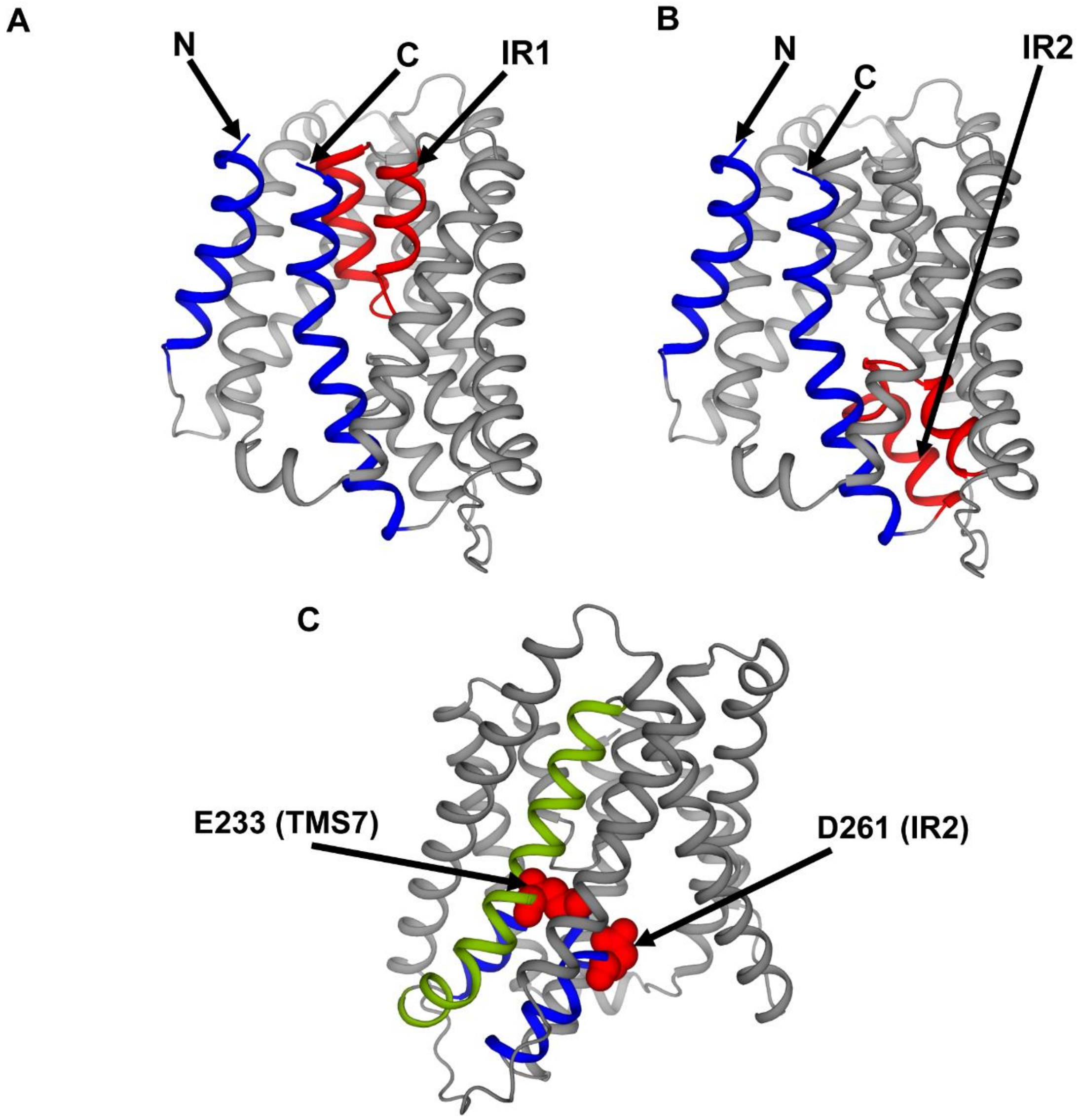

Figure S5. Depiction of the intramembrane regions IR1 (A) and IR2 (B) in the AlphaFold2 model of LysO. The two intramembrane regions are highlighted in red in A and B. The N and C-termini of LysO are marked. TMS1 and TMS are coloured blue, and the rest of the molecule is coloured grey. (C) The two acidic residues E233 in TMS7 (green) and D261 in IR2 (blue) are marked and represented in the model as spheres representated in red. Rest of the molecule is coloured grey.

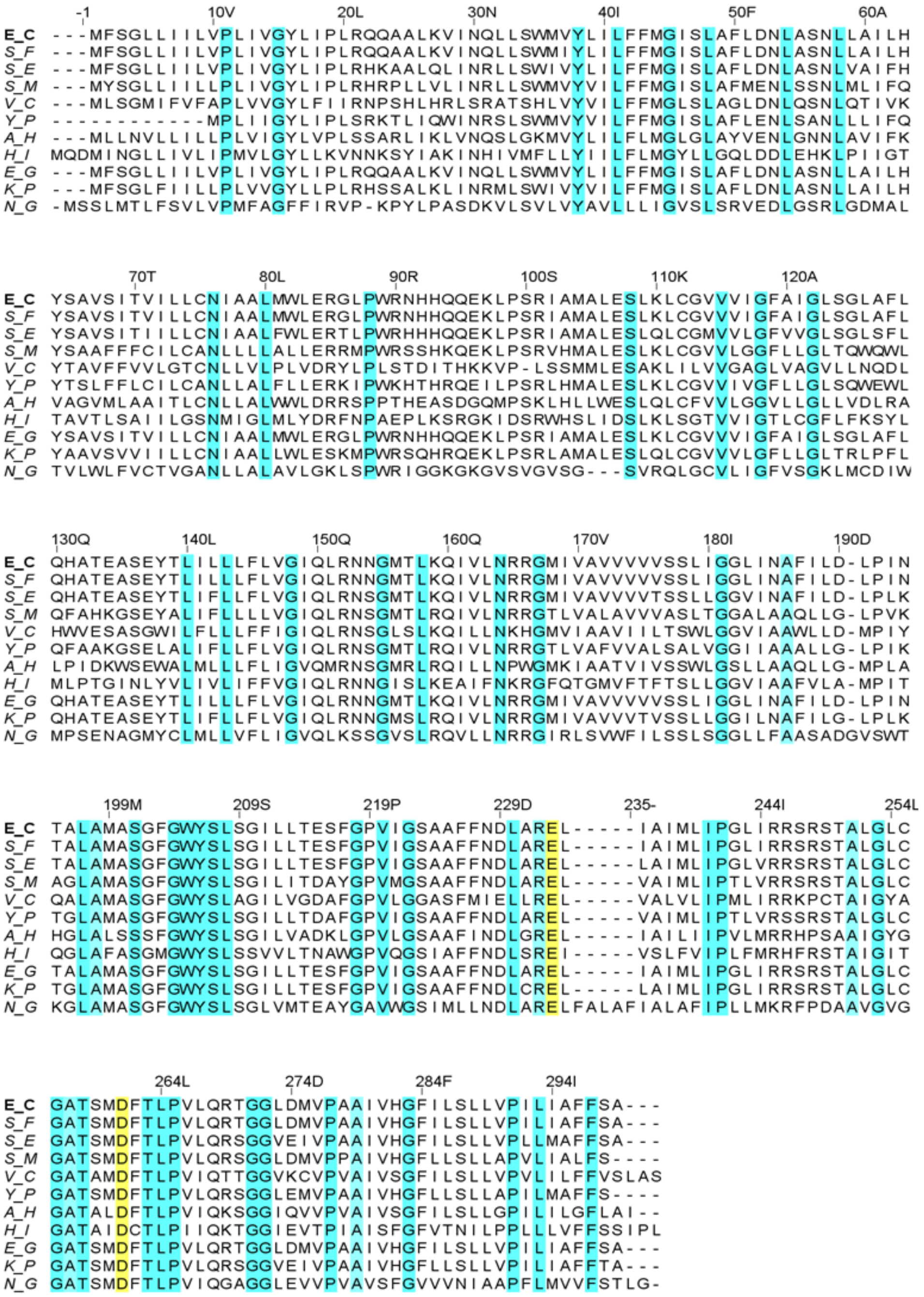

Figure S6. Multiple sequence alignment of orthologs of LysO. The sequence alignment, visualization and editing were performed in Jalview (75) version 2.11.1.4, using Clustal W and Clustal X. Conserved residues are highlighted in cyan and the conservation of two negatively charged residues is highlighted in yellow. E_C, S_F, S_E, S_M, V_C, Y_P, A_H, H_I, E_G, K_P, N_G stand for *E. coli*, *S. flexneri*, *S. enterica*, *S. marcescens*, *V. cholerae*, *Y. pestis*, *A. hydrophila*, *H. influenzae*, *E. gallinarum*, *K. pneumoniae* and *N. gonorrhoeae* respectively.

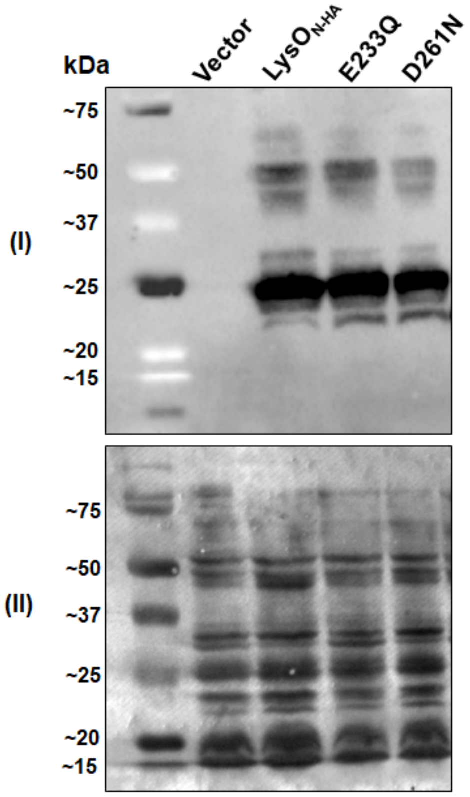

Figure S7. Expression level of LysO$_{N\text{-}HA}$, and its D261N and E233Q substituted derivatives in inside-out vesicles. Samples from preparations of inside-out vesicles of the strain GJ16375 bearing the vector (pBAD18) and plasmids expressing LysO$_{N\text{-}HA}$, and its D261N and E233Q substituted derivatives, were mixed with SDS loading buffer, subjected to SDS-PAGE and the transferred blot was probed with anti-HA antibodies (I). The same blot was stained with Amido Black (II). A 20 µl aliquot of inside-out vesicles from the vector sample and 40 µl aliquots of vesicles from other sample, were mixed with SDS loading buffer, the volume was adjusted to 100 µl, and equal volumes from all samples were loaded. LysO$_{N\text{-}HA}$ and its E233Q and D261N substituted derivatives are expressed from the plasmids pHYD6240, pHYD6402 and pHYD6401 respectively.

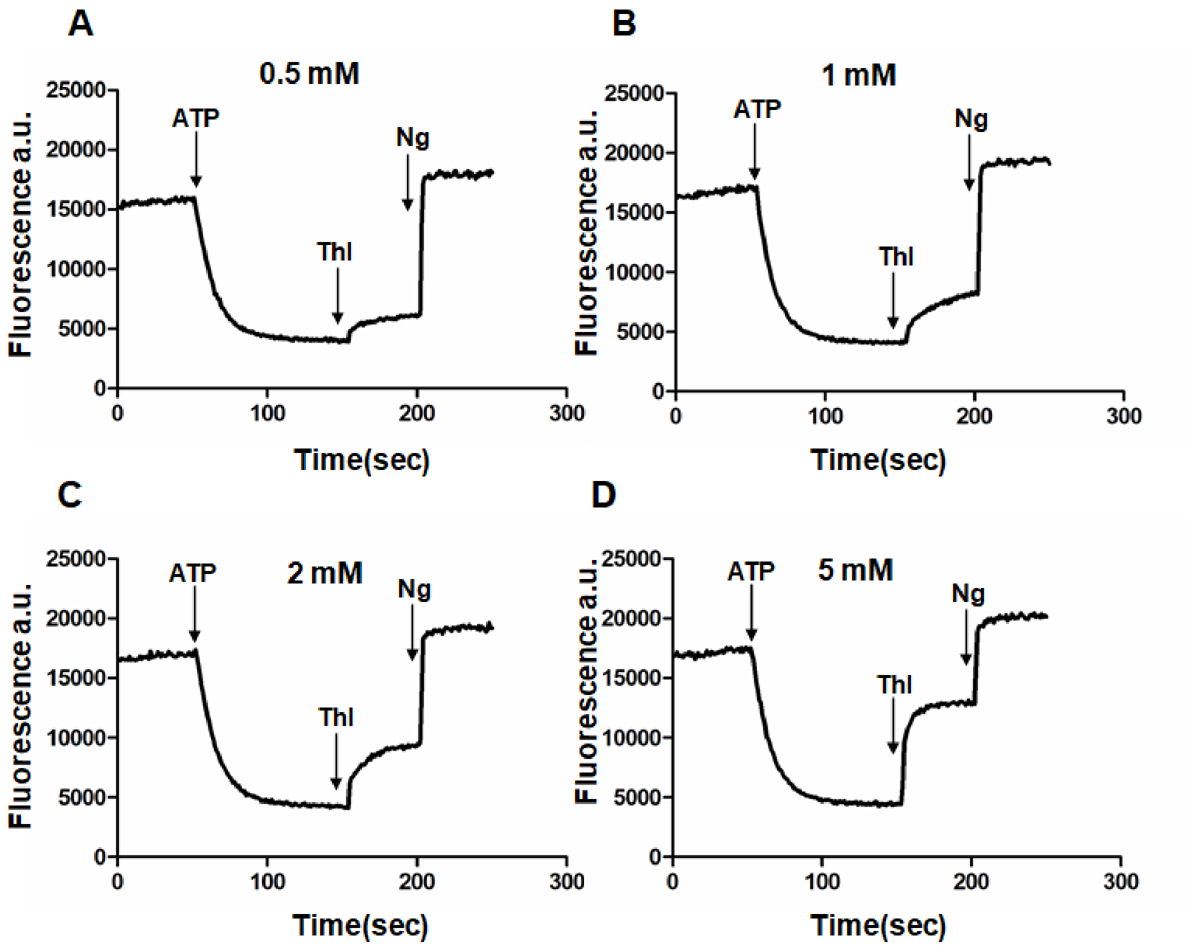

Figure S8. Dose dependent alterations in L- thialysine (Thl) induced proton fluxes mediated by LysO. Inside-out vesicles of the strain GJ16375 bearing overexpressed LysO$_{N\text{-}HA}$ were exposed to the pH sensitive fluorescent dye ACMA. Quenching of ACMA fluorescence following addition of ATP, its recovery following Thl addition (0.5, 1, 2 and 5 mM; A-D) and its eventual recovery after addition of nigericin (Ng), was recorded. ATP, Thl and Ng were added to vesicle preparations at the 50th, 150th and 200th second respectively. The experiment was performed twice with a single batch of inside-out vesicles and traces from one trial are shown. The plasmid used for overexpression of LysO$_{N\text{-}HA}$ is pHYD6240.

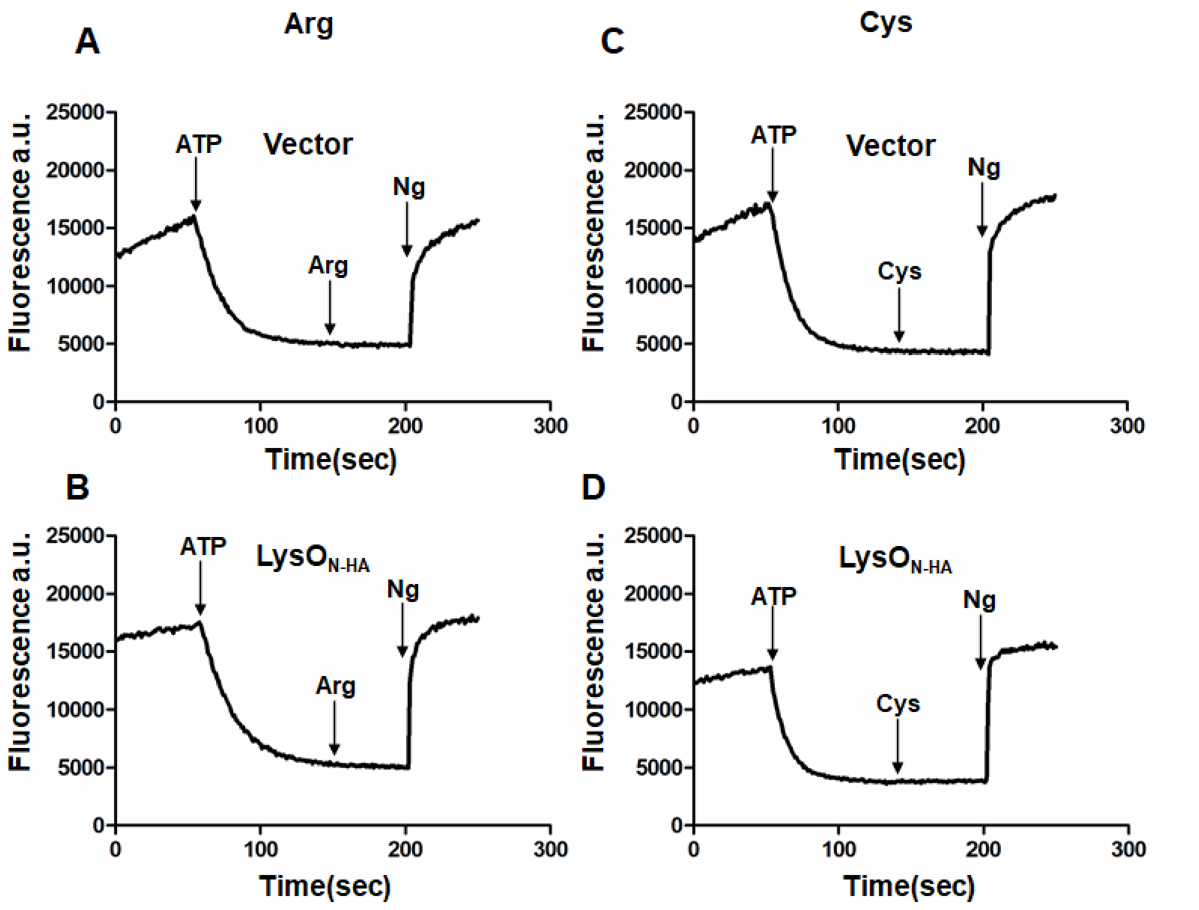

Figure S9. Absence of LysO mediated proton release by L-arginine (Arg) and L-cysteine (Cys) in inside-out vesicles. Inside-out vesicles were prepared from the strain GJ16375 bearing the plasmid pBAD18 (vector) and GJ16375 bearing $LysO_{N-HA}$ overexpressed from the plasmid pHYD6240. Pairs of vesicle preparations from the vector and the $LysO_{N-HA}$ overexpression strain, were treated with ACMA, ATP, the amino acids Arg (5 mM; A, B) or Cys (5 mM; C, D) and nigericin (Ng), and changes in ACMA fluorescence recorded as described in figure S8. The experiment was performed twice with a single batch of inside-out vesicles bearing vector and $LysO_{N-HA}$ and traces from one trial are shown.

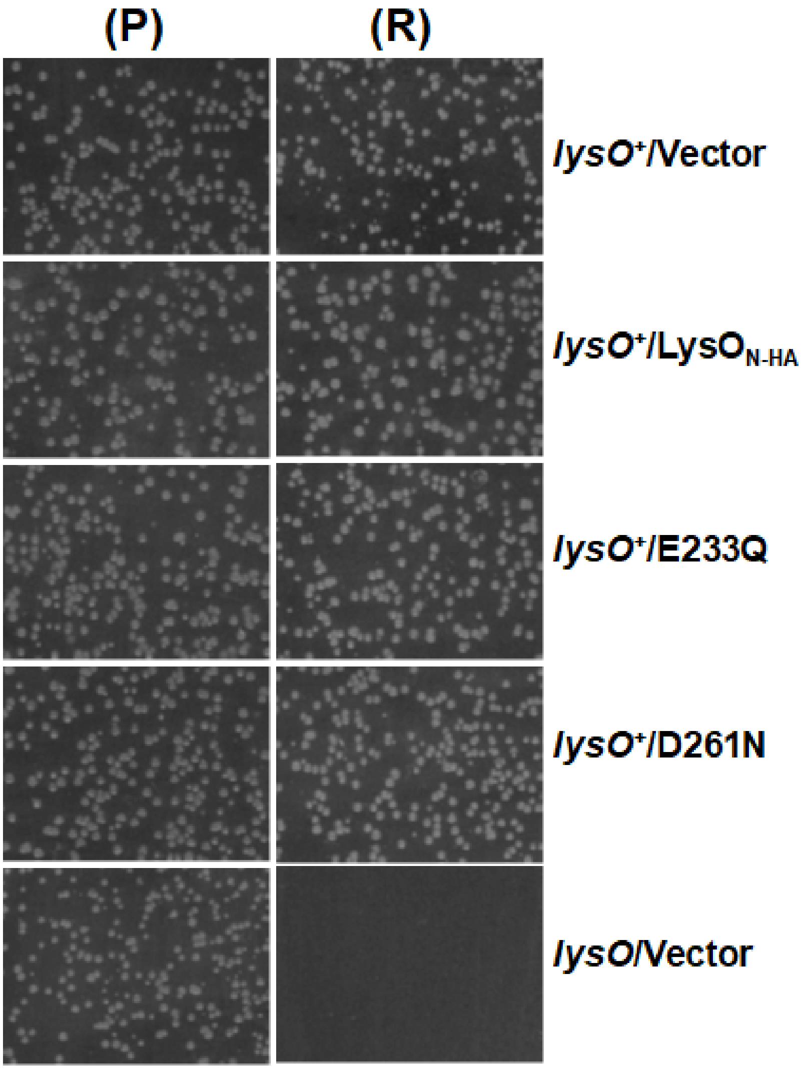

Figure S10. Overexpression of $LysO_{N\text{-}HA}$ bearing the E233Q and D261N substitutions does not impair the function of chromosomally encoded LysO. A_{600} normalized cultures of MC4100 (*lysO*$^{+}$) bearing the vector (pHYD5001) and its derivatives expressing $LysO_{N\text{-}HA}$ and $LysO_{N\text{-}HA}$ bearing the E233Q and the D261N substitutions, were washed, diluted to $3X10^{-6}$ in glucose minimal A broth and the diluted cultures (100 µl) were spread on the permissive (P) and restrictive (R) growth media that are glucose Minimal A agar and glucose Minimal A agar containing thialysine at 1 µg/ml respectively. The two plates also contained IPTG at 1 mM. A culture of the Δ*lysO*::Kan mutant (*lysO*) bearing the vector was also processed as above. The various LysO proteins are under the expression control of the P_{trc} promoter and plasmids encoding $LysO_{N\text{-}HA}$ and its E233Q and D261N derivatives are pHYD5579, pHYD6252 and pHYD6256 respectively. Sections of the scanned agar plates following growth of the bacterial colonies, are displayed.

References

7. Pathania, A., and Sardesai, A. A. (2015) Distinct paths for basic amino acid export in *Escherichia coli*: YbjE (LysO) mediates export of L-lysine. *J. Bacteriol.* **197**, 2036–2047

23. Beja, O., Bibi, E., and Kaback, H. R. (1996) Functional expression of mouse MdrI in an outer membrane permeability mutant of *Escherichia coli*. **93**, 5969–5974

25. Jumper, J., Evans, R., Pritzel, A., Green, T., Figurnov, M., Ronneberger, O., Tunyasuvunakool, K., Bates, R., Žídek, A., Potapenko, A., Bridgland, A., Meyer, C., Kohl, A. A., Ballard, A. J., Cowie, A., Romera-Paredes, B., Nikolov, S., Jain, R., Adler, J., Back, T., Petersen, S., Reiman, D., Clancy, E., Zielinski, M., Steinegger, M., Pacholska, M., Berghammer, T., Bodenstein, S., Silver, D., Vinyals, O., Senior, A. W., Kavukcuoglu, K., Kohli, P., and Hassabis, D. (2021) Highly accurate protein structure prediction with AlphaFold. *Nature*. 10.1038/s41586-021-03819-2

26. Baek, M., DiMaio, F., Anishchenko, I., Dauparas, J., Ovchinnikov, S., Lee, G. R., Wang, J., Cong, Q., Kinch, L. N., Schaeffer, R. D., Millán, C., Park, H., Adams, C., Glassman, C. R., DeGiovanni, A., Pereira, J. H., Rodrigues, A. V., Dijk, A. A. van, Ebrecht, A. C., Opperman, D. J., Sagmeister, T., Buhlheller, C., Pavkov-Keller, T., Rathinaswamy, M. K., Dalwadi, U., Yip, C. K., Burke, J. E., Garcia, K. C., Grishin, N. V., Adams, P. D., Read, R. J., and Baker, D. (2021) Accurate prediction of protein structures and interactions using a three-track neural network. *Science.* 10. 1126/science.abj8754

39. Pathania, A., Gupta, K., Dubey, S., Gopal, B., and Sardesai, A. (2016) The Topology of the L-Arginine Exporter ArgO Conforms to an N in -C out Configuration in *Escherichia coli*: Requirement for the Cytoplasmic N-Terminal Domain, Functional Helical Interactions, and an Aspartate Pair for ArgO Function. *J. Bacteriol.* **198**, 3186–3199

44. Bernsel, A., Viklund, H., Falk, J., Lindahl, E., von Heijne, G., and Elofsson, A. (2008) Prediction of membrane-protein topology from first principles. *Proc. Natl. Acad. Sci. U. S. A*. **105**, 7177–7181

59. Baba, T., Ara, T., Hasegawa, M., Takai, Y., Okumura, Y., Baba, M., Datsenko, K. A., Tomita, M., Wanner, B. L., and Mori, H. (2006) Construction of *Escherichia coli* K-12 in-frame, single-gene knockout mutants: the Keio collection. *Mol. Syst. Biol.* **2**, 2006.0008

62. Wessel, D., and Flügge, U. I. (1984) A method for the quantitative recovery of protein in dilute solution in the presence of detergents and lipids. *Anal. Biochem.* **138**, 141–143

63. Sharma, R. (2019) *Roles of the PtsP-PtsO-PtsN phosphorelay in K^+ metabolism in Escherichia coli*. PhD thesis, Manipal Academy of Higher Education

64. Amann, E., Ochs, B., and Abel, K. J. (1988) Tightly regulated *tac* promoter vectors useful for the expression of unfused and fused proteins in *Escherichia coli*. *Gene*. **69**, 301–15

65. Sharma, R., Shimada, T., Mishra, V. K., Upreti, S., and Sardesai, A. A. (2016) Growth inhibition by external potassium of *Escherichia coli* lacking PtsN ($EIIA_{Ntr}$) is caused by potassium limitation mediated by YcgO. *J. Bacteriol.* **198**, 1868–1882

66. Kleckner, N., Bender, J., and Gottesman, S. (1991) Uses of transposons with emphasis on Tn*10*. *Methods Enzymol*. **204**, 139–180

67. Guzman, L. M., Belin, D., Carson, M. J., and Beckwith, J. (1995) Tight regulation, modulation, and high-level expression by vectors containing the arabinose P_{BAD} promoter. *J. Bacteriol.* **177**, 4121–4130

68. Krogh, A., Larsson, B., von Heijne, G., and Sonnhammer, E. L. L. (2001) Predicting transmembrane protein topology with a hidden Markov model: Application to complete genomes. *J. Mol. Biol.* **305**, 567–580

69. Käll, L., Krogh, A., and Sonnhammer, E. L. L. (2004) A combined transmembrane topology and signal peptide prediction method. *J. Mol. Biol.* **338**, 1027–1036

70. Viklund, H., and Elofsson, A. (2008) OCTOPUS: improving topology prediction by two-track ANN-based preference scores and an extended topological grammar. *Bioinformatics*. **24**, 1662–1668

71. Dobson, L., Reményi, I., and Tusnády, G. E. (2015) CCTOP: A Consensus Constrained TOPology prediction web server. *Nucleic Acids Res.* **43**, W408–W412

72. Tusnady, G. E., and Simon, I. (2001) The HMMTOP transmembrane topology prediction server. *Bioinformatics*. **17**, 849–850

73. Cao, B., Porollo, A., Adamczak, R., Jarrell, M., and Meller, J. (2006) Enhanced recognition of protein transmembrane domains with prediction-based structural profiles. *Bioinformatics*. **22**, 303–309

74. Nugent, T., and Jones, D. T. (2009) Transmembrane protein topology prediction using support vector machines. *BMC Bioinformatics*. **10**, 159

75. Waterhouse, A. M., Procter, J. B., Martin, D. M. A., Clamp, M., and Barton, G. J. (2009) Jalview Version 2-A multiple sequence alignment editor and analysis workbench. *Bioinformatics*. **25**, 1189–1191
